# Combination of multicomponent KA^2^ and Pauson–Khand reactions: short synthesis of spirocyclic pyrrolocyclopentenones

**DOI:** 10.3762/bjoc.16.23

**Published:** 2020-02-12

**Authors:** Riccardo Innocenti, Elena Lenci, Gloria Menchi, Andrea Trabocchi

**Affiliations:** 1 Department of Chemistry “Ugo Schiff”, University of Florence, Via della Lastruccia 13, 50019 Sesto Fiorentino, Florence, Italy,; 2 Interdepartmental Center for Preclinical Development of Molecular Imaging (CISPIM), University of Florence, Viale Morgagni 85, 50134 Florence, Italy

**Keywords:** chemical libraries, chemoinformatics, Cu-catalysis, cycloadditions, molecular scaffolds, multicomponent reactions

## Abstract

The Cu-catalyzed multicomponent ketone–amine–alkyne (KA^2^) reaction was combined with a Pauson–Khand cycloaddition to give access of unprecedented constrained spirocyclic pyrrolocyclopentenone derivatives following a DOS couple-pair approach. The polyfunctional molecular scaffolds were tested on the cyclopentenone reactivity to further expand the skeletal diversity, demonstrating the utility of this combined approach in generating novel spiro compounds as starting material for the generation of chemical libraries. The chemoinformatics characterization of the newly-synthesized molecules gave evidence about structural and physicochemical properties with respect to a set of blockbuster drugs, and showed that such scaffolds are drug-like but more spherical and three-dimensional in character than the drugs.

## Introduction

**Figure 1:**
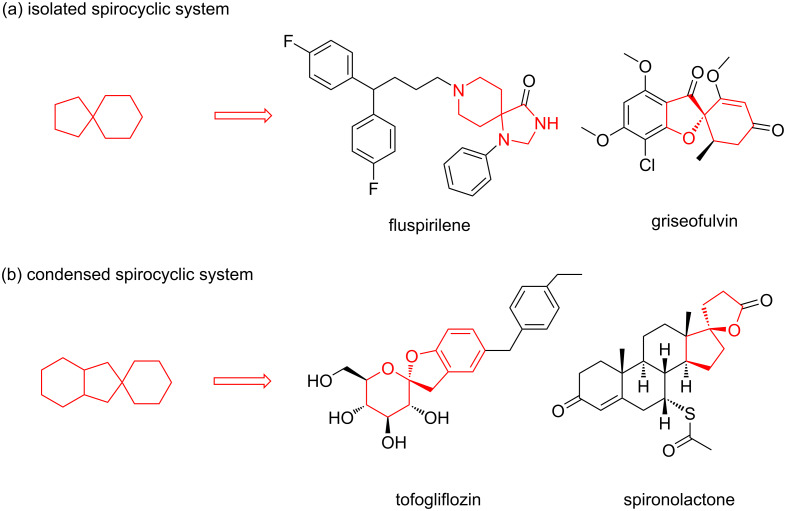
Chemical structure of representative approved drugs containing a spirocyclic moiety.

The screening of small molecule libraries is a well-established approach in early-stage drug discovery to identify hit candidates for the development of drug leads. The application of unconventional molecular scaffolds to develop chemical libraries can increase the chance of finding compounds able to address the so-called “undruggable” targets, such as protein–protein interactions [1]. In this context, molecules containing one or more rings are of primary interest, as they will suffer a reduced conformational entropy penalty upon binding to a protein target, and the approach of constraining the ligand conformation with a ring is widely used in drug design [2]. Accordingly, with increasing interest for sp^3^-rich molecules, spirocyclic compounds are being considered valuable as molecular platforms for the generation of high-quality small molecule collections, taking advantage of the stereochemical diversity, and of their three-dimensional shape and structural bias to develop lead compounds, specifically in the field of protein–protein interactions [3–6]. Spiranic rings such as spiroketals are present in numerous natural products [7–9], a wide array of spirocyclic compounds are being studied in drug discovery and their chemical space have been systematically charted and characterized recently by Bajorath and co-workers (Figure 1) [10]. This study revealed that spirocycles are found only in few approved drugs [11] and that there is a significant potential to explore the chemical space of spirocyclic scaffolds, especially in the case of the condensed ones. Thus, new synthetic routes towards the synthesis of building blocks containing spiranic rings have increasingly appeared in the recent literature [12].

**Scheme 1:**
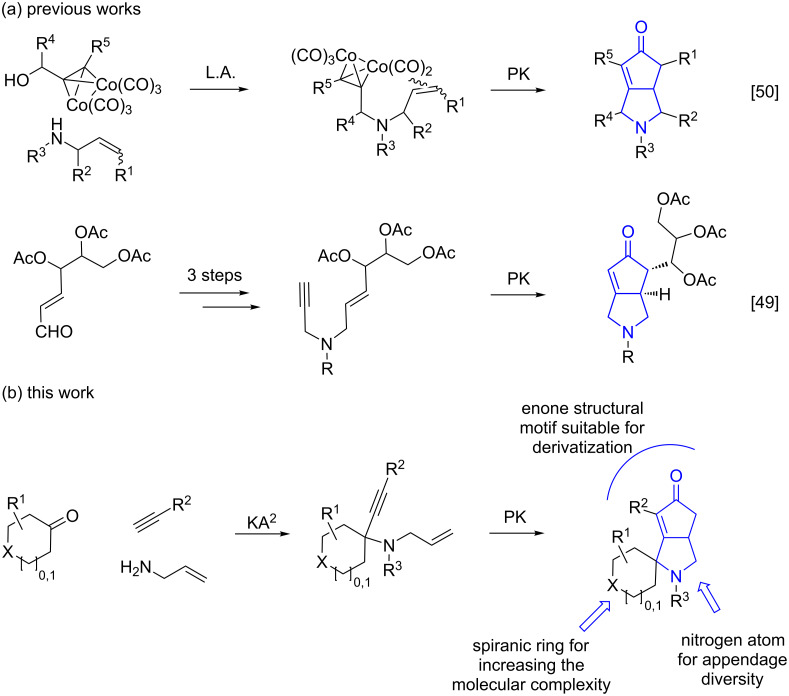
Synthetic strategies for accessing pyrrolocyclopentenone derivatives, including the novel couple/pair approach that combines the KA^2^ and PK multicomponent reactions. L.A. = Lewis acid; PK = Pauson–Khand.

Among the synthetic approaches to improve the quality and quantity of small molecules members of chemical libraries, diversity-oriented synthesis (DOS) [13–16], has been proposed as a paradigm for developing large collections of structurally diverse small molecules in a way to generate the maximum diversity and complexity from simple starting materials applying divergent synthetic strategies, such as the use of complexity-generating reactions and the build/couple/pair approach [17–18]. The application of multicomponent approaches has proven to be very useful as starting points in DOS [19–22], such as the exploitation of the Petasis three-component [23–29] and the Ugi four-component reactions [30–33], showing interesting properties for the generation of compounds characterized by high stereochemical and skeletal diversity. Although not fully exploited so far, some contributions on the diversity-oriented synthesis of spirocyclic compounds have appeared in the literature recently, also employing multicomponent approaches to give the spirocyclic adduct after a cyclization step [34–36]. We recently focused our interest to the cyclopentenone ring [37], as this heterocycle is a powerful synthon for the synthesis of a variety of bioactive target molecules, due to the broad diversity of chemical modifications available for the enone structural motif [38]. The most common approach to access such chemotype is the Pauson–Khand (PK) reaction [39–40], consisting of a [2 + 2 + 1] cycloaddition between an olefin, alkyne, and carbon monoxide. This reaction has been also applied in cascade approaches [41], and in combination with RCM [42], Diels–Alder [43] and Staudinger [44] reactions to produce novel structurally complex chemical entities. Following our interest to DOS as a synthetic strategy for the generation of molecular scaffolds according to a couple/pair approach [45–47], we reasoned to combine the copper-catalyzed ketone–amine–alkyne (KA^2^) multicomponent coupling reaction [48] with the Pauson–Khand cycloaddition as the pairing reaction to achieve spirocyclic pyrrolocyclopentenone derivatives. Specifically, the KA^2^ reaction was envisaged taking into account cyclic ketones, to install a quaternary carbon atom carrying the required 1,6-enyne moiety for the subsequent Pauson–Khand reaction, thus achieving the corresponding tricylic structure in three single steps (Scheme 1b). This unprecedent molecular scaffold represents a valuable template for medicinal chemistry purpose, as the pyrrolocyclopentenone core is contained in a variety of bioactive molecules [49–50], and can serve as an advanced intermediate for the synthesis of different compounds, such as (−)-kainic acid [51–52]. Previous similar approaches reported only planar pyrrolocyclopentenones starting from propargyl alcohol–cobalt complexes and allyl amides [50], or carbohydrate-derived allylpropargylamine [49] (Scheme 1a).

## Results and Discussion

**Scheme 2:**
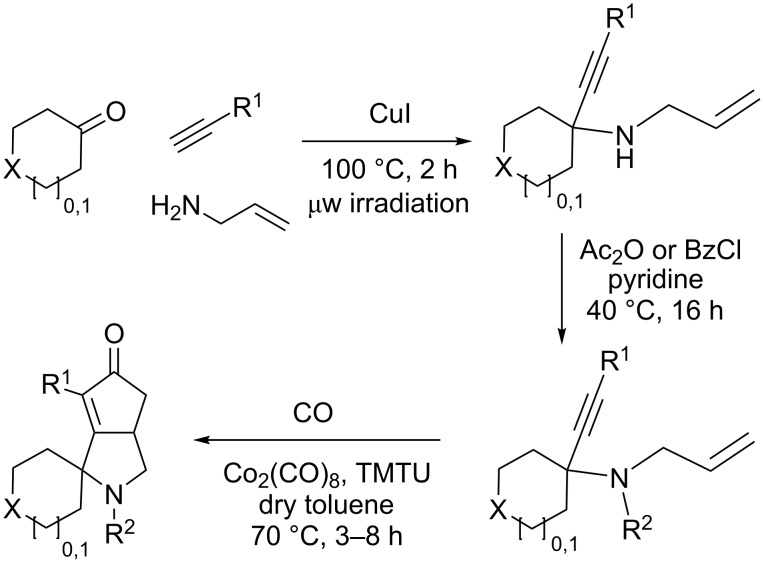
Couple/pair approach using combined KA^2^ and Pauson–Khand multicomponent reactions.

Cyclohexanone (**1**) and phenylacetylene (**2**) were taken into account for the optimization of the KA^2^ reaction conditions with allylamine, in order to attain a quaternary carbon atom containing suitable alkenyl and alkynyl appendages for subsequent Pauson–Khand intramolecular cycloaddition (Scheme 2).

The KA^2^ reaction was assayed following the reported method [48] employing copper catalysis, and tested on our starting material upon variation of copper salts, solvents and temperature, resulting in the neat reaction under CuI catalysis being optimal when carried out for 2 h at 100 °C under microwave irradiation (see Supporting Information File 1), as it can promote metal-catalyzed reactions [53]. The scope of the combined approach employing KA^2^ and Pauson–Khand reactions was studied by varying the alkyne and ketone components, along with the acylating moiety being installed before the Pauson–Khand reaction (Scheme 2 and Table 1). The acylation of the amino group was found necessary to allow for the cobalt-catalyzed reaction to proceed under a CO atmosphere. This step was also carried out in one pot after the KA^2^ reaction by diluting with pyridine and adding the acylating reagent, to achieve the corresponding product in slightly lower yield. Attempts to carry out the Pauson–Khand reaction directly on the amino group before the acylation step did not work, nor using a modified approach using ammonium chloride and 1.5 equivalents of Co_2_(CO)_8_ under an inert atmosphere, as reported for similar reactions in the presence of basic nitrogen atoms [28].

**Table 1:** Scope of the combined KA^2^ and Pauson–Khand multicomponent processes.^a^

entry	ketone	alkyne	yield, %	

			KA^2^ product	PK product

1	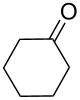 **1**	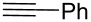 **2**	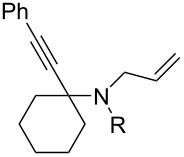 **3**: R = H, 82% **4**: R = Ac, 61%	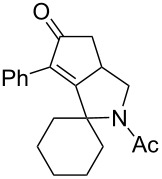 **5**, 73%
2	**1**	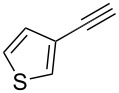 **6**	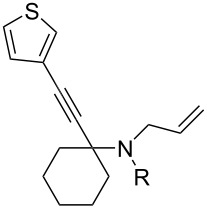 **7**: R = H, 74% **8**: R = Ac, 78%	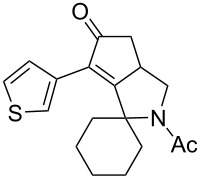 **9**, 68%
3	**1**	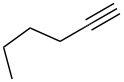 **10**	–	–
4	**1**	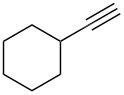 **11**	–	–
5	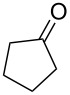 **12**	**2**	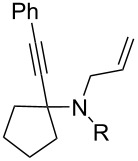 **13**: R = H, 61% **14**: R = Ac, 56%	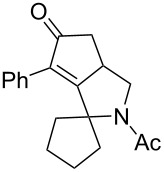 **15**, 72%
6	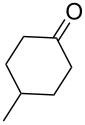 **16**	**2**	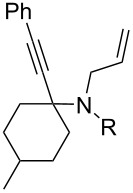 **17**: R = H, 82% **18**: R = Ac, 68%	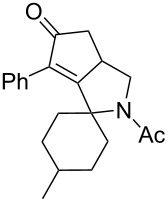 **19**, 72%
7	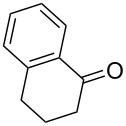 **20**	**2**	–	–
8	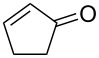 **21**	**2**		
9	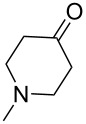 **22**	**2**	–	–
10	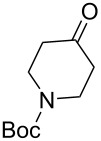 **23**	**2**	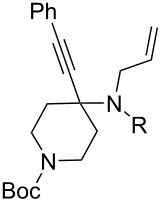 **24**: R = H, 83% **25**: R = Ac, 63%	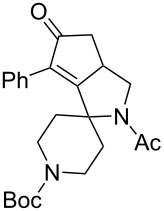 **26**, 79%
11	**23**	**6**	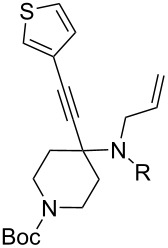 **27**: R = H, 62% **28**: R = Ac, 63%	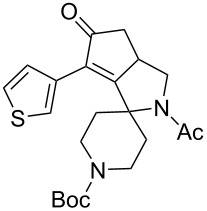 **29**, 68%
12	**23**	**2**	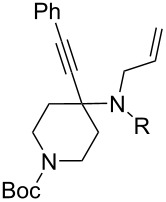 **24**: R = H, 83% **30**: R = Bz, 68%	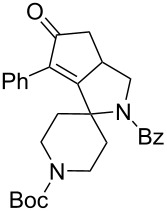 **31**, 78%
13	**23**	**2**	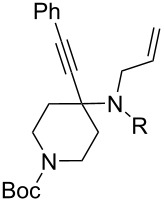 **24**: R = H, 83% **32**: R = Ts, 63%	–
14	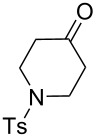 **33**	**2**	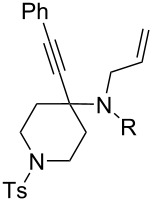 **34**: R = H, 71% **35**: R = Ac, 52%	–

^a^Reaction conditions. KA^2^ reaction: ketone (1 equiv), alkyne (1.2 equiv) and amine (1.2 equiv), CuI (0.2 equiv), 100 °C, 2 h, microwave irradiation. Amine protection: pyridine (2 mL/mmol), acetic anhydride (4 mL/mmol), 40 °C, 16 h. Pauson–Khand reaction: enyne (1 equiv), Co_2_(CO)_8_ (0.1 equiv), *N,N,N’,N’*-tetramethylthiourea (0.6 equiv), toluene (20 mL/mmol), CO atmosphere, 70 °C, 3–8 h.

The variation of the alkyne component proved to give the KA^2^ coupling adduct when aromatic terminal alkynes were used, as shown in Table 1, entries 1 and 2 for those containing phenyl and thienyl moieties, resulting in 82% and 74% yield for the KA^2^ step. Subsequent acylation and pairing steps proved to proceed in good yield, thus furnishing the corresponding spirocyclopentenone derivatives **5** and **9** with an aromatic appendage at the carbonyl alpha carbon. On the contrary, when aliphatic alkynes were applied in the KA^2^ process, no reaction with allylamine and cyclohexanone was achieved, suggesting a role of the aromatic ring in activating the alkyne towards the copper-catalyzed process (Table 1, entries 3 and 4), as previously reported in other works [54]. Use of cyclopentanone, thus varying the ring size of the cyclic ketone, resulted in the conversion to the title spirocyclopentenone derivative, although in slightly lower yield as compared for the homologous ketone (Table 1, entry 5). No conversion to the KA^2^ adduct was achieved by using unsaturated or aromatic ketones (Table 1, entries 7 and 8, respectively), confirming an important role of the electronic content of the components in the outcome of the multicomponent coupling reaction. Similarly, the use of piperidone as the ketone component proved to work only when the amino group was protected as Boc, whereas the *N*-methyl derivative did not proceed to the coupling product (Table 1, entries 10 and 9, respectively). Indeed, the Boc-piperidone furnished the corresponding spirocyclopentenone derivatives upon changing both the aromatic alkyne or the acylating agent (Table 1, entries 10–12). When the Boc group was replaced with the tosyl one as the *N*-substituent, such chemical moiety proved to impair the subsequent Pauson–Khand reaction (Table 1, entry 13), possibly due to a coordinating effect towards the cobalt catalyst. Such an effect was confirmed when the *N*-tosylpiperidone was used as the ketone component, as also in this case the presence of the tosyl group impaired the acetylated KA^2^ adduct from reacting under Pauson–Khand conditions (Table 1,entry 14).

**Scheme 3:**
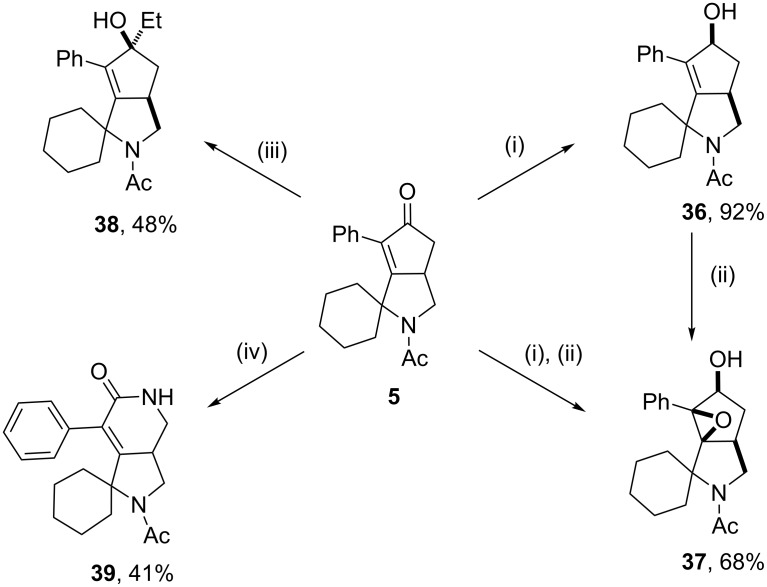
Follow-up chemistry on compound **5** taking advantage of the enone chemistry. Reaction conditions. (i) NaBH_4_ (2 equiv), CeCl_3_^.^7H_2_O (2 equiv), DMC/MeOH 1:1 (20 mL/mmol), 25 °C, 1 h; (ii) *m*-CPBA (1 equiv), DCM (6.5 mL/mmol), 0 °C, 4 h; (iii) EtMgBr 3 M in Et_2_O (5 equiv), CeCl_3_ (1 equiv), THF (6 mL/mmol), 0 °C, 30 min; (iv) NaN_3_ (1.8 equiv), TFA (5 mL/mmol), reflux, 16 h.

The synthetic utility of the spiro derivatives resulting from the combined KA^2^/Pauson–Khand process to generate second-generation molecular scaffolds was tested on compound **5** by applying representative reactions on the enone structural motif (Scheme 3).

The chemoselective carbonyl reduction to obtain the corresponding allylic alcohol derivative **36** was achieved in 92% under Luche reduction conditions employing NaBH_4_/CeCl_3_ in MeOH/DCM, resulting in the selective synthesis of the *syn*-alcohol, as a consequence of the formation of the equatorial alcohol favored by reduced gauche interactions [55]. Subsequent epoxidation at the double bond directed by the hydroxy group and using *m*-chloroperbenzoic acid allowed to install two additional stereocenters with complete control of the relative stereochemistry in 68% yield. Such two-step synthesis proved to proceed also in one-pot, resulting in the generation of the stereochemically dense epoxyalcohol **37** in 68% overall yield. The treatment of compound **5** with EtMgBr as a Grignard reagent in the presence of CeCl_3_ gave the corresponding tertiary alcohol **38** with similar stereochemical features as of **36** in the formation of the equatorial alcohol, although in lower yield. The use of CeCl_3_ together with EtMgBr was found particularly effective to suppress conjugate additions, with similar yield as reported for analogous substrates [56]. Subsequent acid-catalyzed displacement of the hydroxy moiety with aniline in the presence of camphorsulfonic acid did not give the desired amine, supporting the hypothesis of steric hindrance at such position [57]. Similarly, Simmons–Smith cyclopropanation reaction [58] did not work, and so as for the cycloaddition reaction with Danishefsky’s diene, possibly due to steric hindrance imposed by the adjacent phenyl and cyclohexyl rings [59]. The treatment of compound **5** under Schmidt reaction conditions with sodium azide in TFA [60] resulted in the conversion to the corresponding six-membered ring lactam **39** in 41% yield, demonstrating the reactivity of the enone **5** at the carbonyl group and showing stability towards harsh acidic conditions.

**Figure 2:**
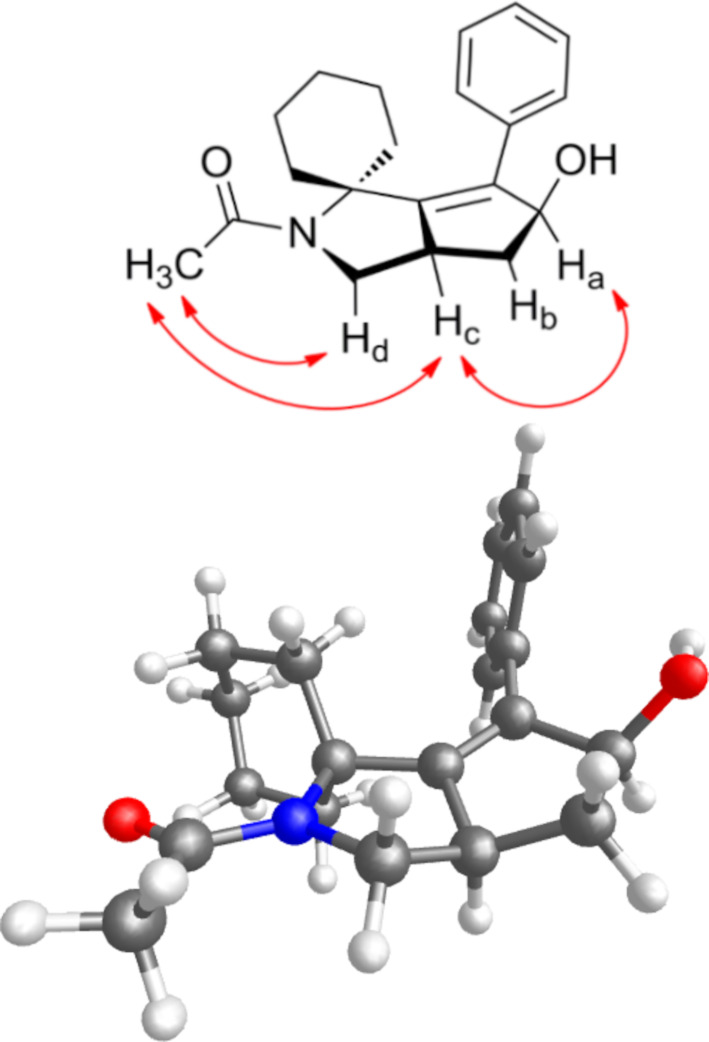
Top: Selected NOE contacts from NOESY 1D spectra of compound **36**; bottom: low energy conformer of **36**, Z rotamer, using ab initio calculation at the HF/3-21G* level.

The structural assignment of compound **36** was assessed by detailed 1D and 2D NMR studies, and corroborated with molecular modeling calculations. NOESY-1D experiments carried out with a mixing time of 500 ms allowed to identify the unique rotamer possessing a Z geometry, as evinced by a NOE interaction between H_d_ and the methyl group. The *cis* relationship between the OH group and the pyrrolidine ring, resulting from the chemo- and stereoselective *syn* reduction of the carbonyl group, was evinced by NOESY-1D experiments showing intense NOE effects between H_c_ and H_a_ protons, as also shown in NOESY 2D spectrum (see Figure 2 and Supporting Information File 1). A similar analysis allowed the structural assignment for **38**.

### Chemoinformatic analysis

**Figure 3:**
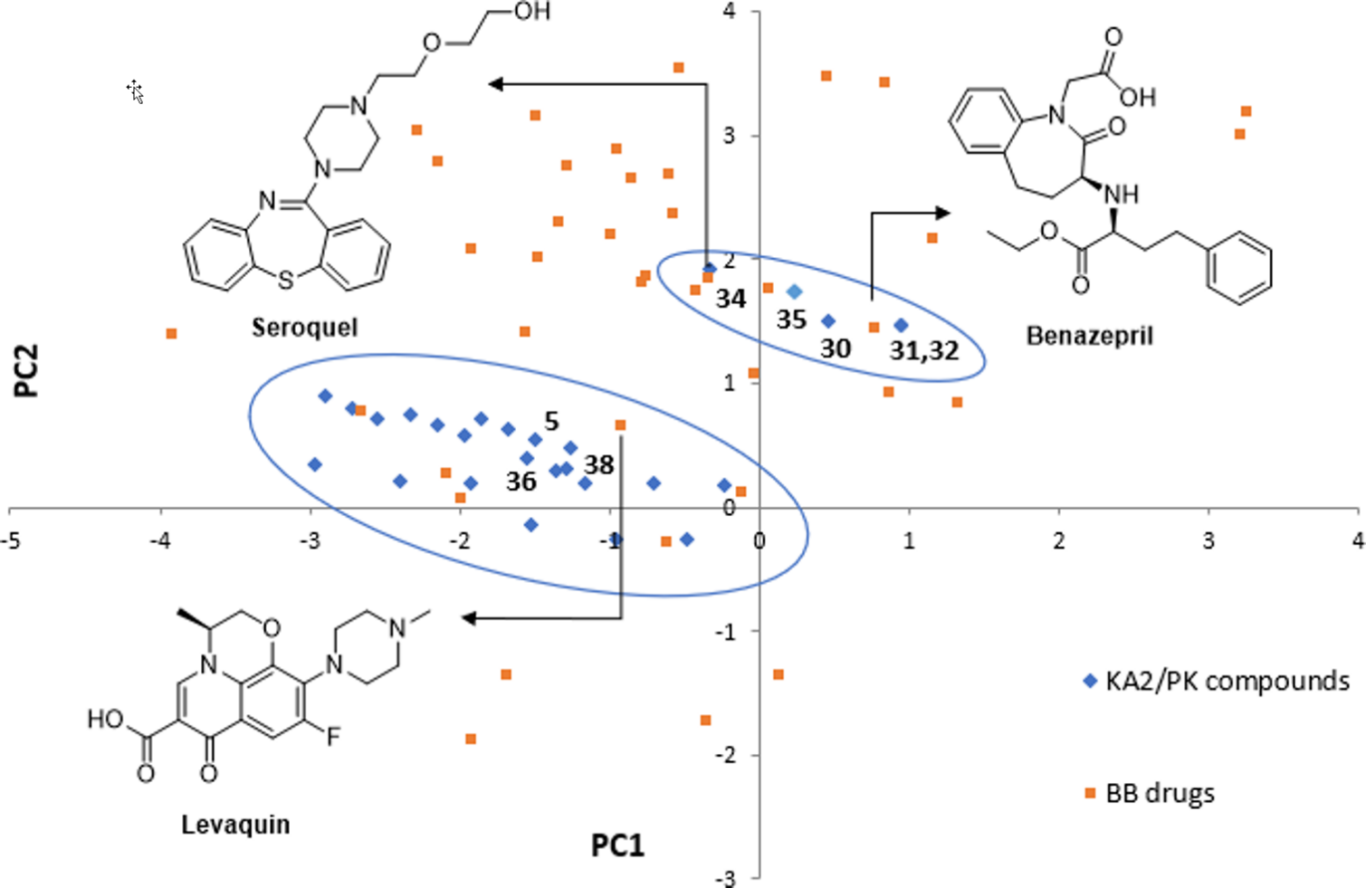
PCA plot resulting from the correlation between PC1 vs PC2, showing the positioning in the chemical space of compounds **3**–**39** (blue diamonds) with respect to the reference set of brand-name blockbuster drugs (orange squares).

The structural features of the compounds so obtained and representative functionalized molecular scaffolds were analyzed in terms of chemical properties and shape analysis in the context of the chemical space [61] using principal component analysis (PCA) and principal moments of inertia (PMI) analysis. PCA is a statistical tool to condense multidimensional chemical properties (i.e., molecular weight, logP, ring complexity) into single dimensional numerical values (principal components), to simplify the comparison with different sets of compounds. ChemGPS-NP [62–64] was chosen for the PCA analysis, providing a comprehensive exploration of the chemical space in terms of global mapping onto a consistent 8-dimensional map of structural characteristics [65]. In particular, the first and the second dimensions (PC1 and PC2) are the most interesting ones, being associated respectively with size, shape, and polarizability and with aromatic and conjugation related properties. The analysis of PC1 vs PC2 of compounds **3–39**, in comparison with a reference set of 40 brand-name blockbuster drugs [66–67] (Figure 3), showed the different distribution of compounds **3**–**39** in two different clusters. Most of the compounds reside in the first cluster, positioned in the negative direction of x axis, in a region that shows good overlap with drugs like levaquin, which is characterized by a complex tricyclic skeleton. The addition of a second aromatic ring, as the benzoyl or tosyl group of compounds **30**–**32**, **34** and **35**, increased the aromatic- and conjugation-related character of the structure, thus resulting in shifting those compounds to a second cluster being positioned in the positive direction for both axes, together with drugs possessing large aromatic content, as benazepril and seroquel.

**Figure 4:**
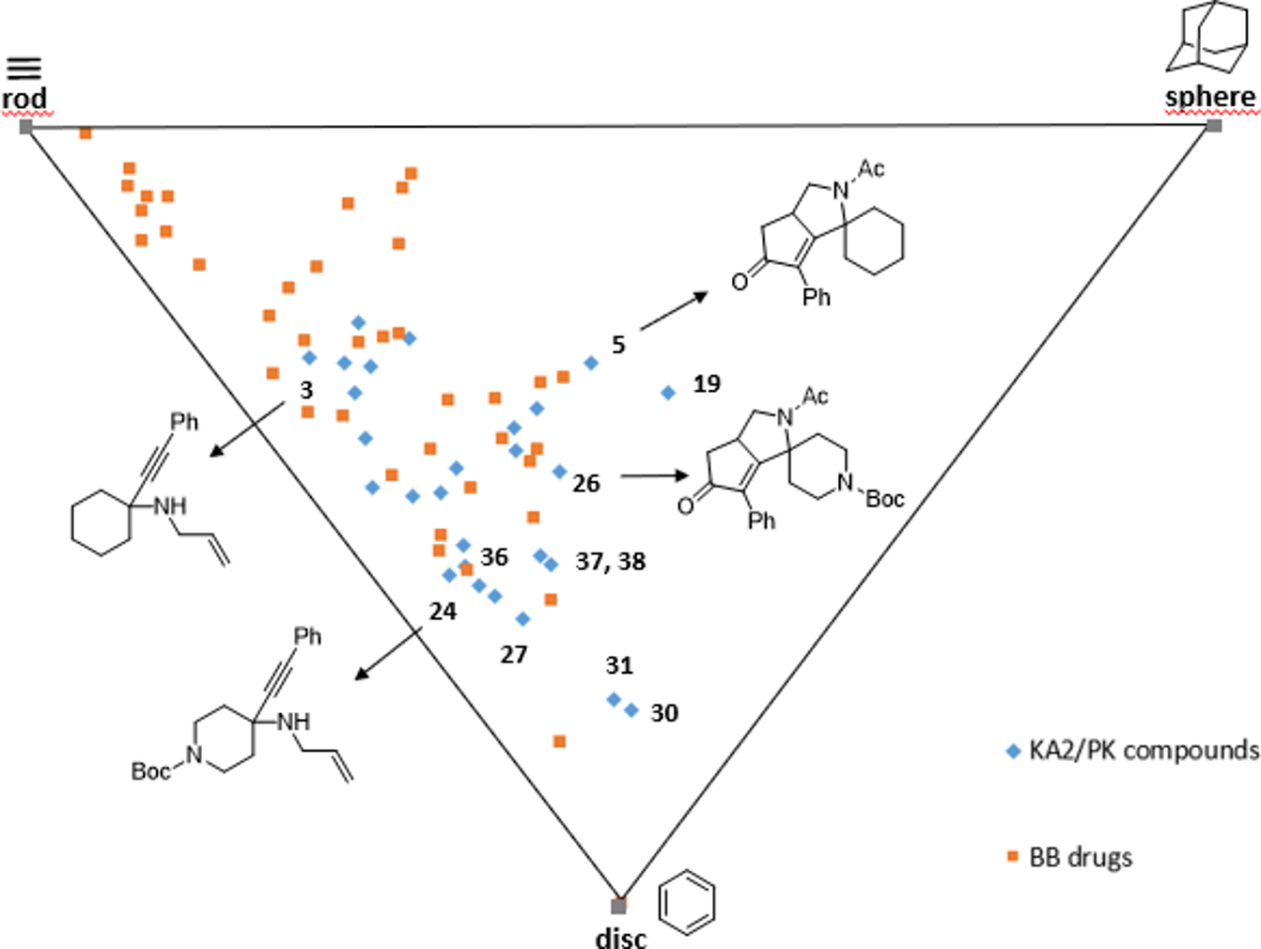
PMI plot showing the skeletal diversity of compounds **3**–**39** (blue diamonds) with respect to the reference set of brand-name blockbuster drugs (orange squares).

The principal moments of inertia (PMI) analysis was also taken into account for the three-dimensional shape analysis of compounds **3**–**39** in the context of chemical space, again with reference to a set of BB drugs. The three principal moments of inertia (*I*_xx_, *I*_yy_, *I*_zz_) and the corresponding normalized principal moments of inertia were determined according to Sauer and Schwarz [68] for the lowest energy conformation of all the compounds and the reference drugs. Then, the normalized PMI ratios were plotted on a triangular graph where the vertices (0,1), (0.5,0.5), and (1,1) represent a perfect rod (i.e., 2-butyne), disc (i.e., benzene), and sphere (i.e., adamantane), respectively (Figure 4). This analysis showed that all compounds **3**–**39** possess lower tendency to stay in the rod side of the triangle, as compared to BB drugs, suggesting for these compounds a higher shape complexity, as due to the presence of quaternary carbon atoms introduced by the KA^2^ coupling reaction. The intramolecular Pauson–Khand cyclization proved to be even more efficient in increasing the three-dimensional character of these compounds, as spiro tricyclic products were found to be more shifted towards the sphere-disc region of this chemical space, especially if compared to their corresponding starting materials (see Figure 4, compounds **5** and **26** with respect to **3** and **24**, respectively). This feature is promising in view of expanding the array of molecular scaffolds of this nature for drug discovery purpose, as a higher scaffold complexity is generally associated with a more successful outcome in drug discovery and development [69–71]. On the other hand, the reduction of the carbonyl group into an alcohol was not significant in increasing the three-dimensional character of the structure, as compounds **36**–**38** were found to be more shifted towards the rod-sphere axes as compared to the parent compound **5**.

## Conclusion

Spirocyclic compounds are valuable molecular platforms for the generation of high-quality small molecule collections, taking advantage of their three-dimensional shape and structural bias to develop lead compounds. The combination of multicomponent KA^2^ and Pauson–Khand reactions using representative cyclic ketones, allylamine and phenylacetylene gave access to highly constrained spirocyclopentenone derivatives following a DOS couple-pair approach. A representative spirocyclopentenone derivative was applied to follow-up chemistry employing the enone reactivity to further expand the skeletal diversity, resulting in additional chemotypes useful as starting compounds for appendage diversity in the generation of chemical libraries. The chemoinformatics characterization of the newly-synthesized molecules gave evidence about structural and physicochemical properties with respect to a set of blockbuster drugs, and showed that such scaffolds are drug-like but more spherical and three-dimensional in character than the drugs. These combined approaches are being applied in chemistry as more efficient synthetic approaches to expand the array of polyfunctional sp^3^-rich molecular scaffolds in the effort of increasing the synthetically-accessible chemical space.

## Experimental

**General procedure (A) for the KA^2^ coupling reaction.** CuI (0.2 equiv) was added in a dry sealed vial for microwave synthesis under a nitrogen flow. Then, ketone (1 equiv), alkyne (1.2 equiv) and amine (1.2 equiv) were successively added under a nitrogen flow, and the mixture was heated under microwave irradiation to 100 °C for 2 h. Then, EtOAc was added and the organic phase was washed with 5% NH_4_OH (3 × 20 mL) and brine. The organic phase was dried with Na_2_SO_4_ and concentrated under reduced pressure. The crude product was purified by flash chromatography using the indicated solvent mixture as eluent.

**General procedure (B) for the amine protection.** The KA^2^ product was dissolved in pyridine (2 mL/mmol) and acetic anhydride (4 mL/mmol) was added dropwise to the reaction mixture at 0 °C. Then, the reaction mixture was heated to 40 °C for 16 h, followed by EtOAc addition. The organic phase was washed with 1 M HCl (3 × 20 mL), satd. Na_2_CO_3_ (3 × 20 mL) and brine. The organic phase was dried over Na_2_SO_4_ and concentrated under reduced pressure. The crude product was purified by flash chromatography using the indicated solvent mixture as eluent.

**General procedure for the Pauson–Khand (C) reaction.** In a dry round bottom flask under a nitrogen flow Co_2_(CO)_8_ (0.1 equiv), *N*,*N*,*N*’,*N*’-tetramethylthiourea (0.6 equiv) and a solution of the enyne compound (1 equiv) were successively added in dry toluene (20 mL/mmol). Then, the reaction mixture was kept under a CO atmosphere and stirred at 70 °C until disappearance of the starting material as monitored by TLC. Then, the mixture was filtered on Celite and concentrated under reduced pressure. The crude product was purified by flash chromatography using the indicated solvent mixture as eluent.

**Molecular modelling.** Calculations were performed using SPARTAN Version 5.11. Conformational searches of **36** were carried out using Monte Carlo method within MMFF94 force field, and the AM1 semiempirical method [72] was used to optimize the global minimum conformer. The geometry of the most abundant minimum energy conformer was successively subjected to ab initio single point energy calculation at the 3-21G*/ HF level of quantum chemical theory.

**PCA analysis.** The web-based public tool ChemGPS-NP was used for PCA analysis of compounds **3**–**39**, to compare their chemical properties with those of blockbuster drugs. ChemGPS-NP can be applied for comprehensive chemical space navigation and exploration in terms of global mapping on to a consistent 8-dimensional map of structural characteristics. The first four dimensions of the ChemGPS-NP map capture 77% of data variance. Chemical compounds were positioned onto this map using interpolation in terms of PCA score prediction. SMILES codes for all compounds were retrieved using ChemBioDraw Ultra 12.0 and submitted to ChemGPS-NP for achieving the corresponding PC scores (see Supporting Informations). The PCA data were then used for the construction of PC1 vs PC2.

**PMI analysis.** Principal moments of inertia analysis was carried out by calculation of the lowest energy conformation of compounds **3**–**39** and block buster drugs. The conformation calculation was performed using the built-in AMMP molecular mechanics algorithm with default parameters of the VEGA ZZ molecular modelling software package v.3.0.1. Once the lowest energy conformer was calculated, the three principal moments of inertia (*I*_xx_, *I*_yy_, *I*_zz_) and normalized principal moments of inertia, npr1 (*I*_xx_/*I*_zz_) and npr2 (*I*_yy_/*I*_zz_) were determined and plotted on a triangular graph with the vertices (0,1), (0.5,0.5) and (1,1) representing a perfect rod, disc and sphere, respectively.

## Supporting Information

File 1 Table of reaction conditions for KA^2^; experimental procedures, characterization data and copies of ^1^H and ^13^C NMR spectra for all new compounds; copies of NOESY-1D, gCOSY, NOESY and cartesian coordinates of compound **36**; Smiles codes, PCA and PMI data for compounds **3**–**39**.
