# Supplementary material for: Combination of multicomponent KA2 and Pauson–Khand reactions: short synthesis of spirocyclic pyrrolocyclopentenones
Source: Beilstein J Org Chem. 2020 Feb 12;16:200–11. doi: 10.3762/bjoc.16.23 (PMC7034245; doi:10.3762/bjoc.16.23)
Supplement: File 1 — Table of reaction conditions for KA2; experimental procedures, characterization data and copies of 1H and 13C NMR spectra for all new compounds; copies of NOESY-1D, gCOSY, NOESY and cartesian coordinates of compound 36; Smiles codes, PCA and PMI data for compounds 3–39. [file Beilstein_J_Org_Chem-16-200-s001.pdf]

BEILSTEIN JOURNAL OF ORGANIC CHEMISTRY

Supporting Information

for

Combination of multicomponent KA^2 and Pauson–Khand reactions: short synthesis of spirocyclic pyrrolocyclopentenones

Riccardo Innocenti, Elena Lenci, Gloria Menchi and Andrea Trabocchi

Beilstein J. Org. Chem. **2020,** *16,* 200–211. doi:10.3762/bjoc.16.23

Table of reaction conditions for KA^2; experimental procedures, characterization data and copies of ^{1}H and ^{13}C NMR spectra for all new compounds; copies of NOESY-1D, gCOSY, NOESY and cartesian coordinates of compound 36; Smiles codes, PCA and PMI data for compounds 3–39

License and Terms: This is a supporting information file under the terms of the Creative Commons Attribution License (https://creativecommons.org/licenses/by/4.0). Please note that the reuse, redistribution and reproduction in particular requires that the authors and source are credited.
The license is subject to the *Beilstein Journal of Organic Chemistry* terms and conditions: (https://www.beilstein-journals.org/bjoc)

Table S1. Reaction conditions for KA[2]	S2
Experimental procedures and characterization data of compounds **3–5**, **7–9**, **13–15**, **17–19**, **24–32**, **34–39**	S3–S17
^{1}H and ^{13}C NMR spectra of compounds **3–5**, **7–9**, **13–15**, **17–19**, **24–32**, **34–39**	S18–S44
Selected NOE contacts from NOESY1D spectra of compound **36**	S45
gCOSY and NOESY spectra of compound **36**	S46
Table S2. Cartesian coordinates of compound **36** as in the PDB structure	S47–S48
Table S3. SMILES codes of newly-synthesized compounds **3**–**39**	S49
Table S4. PCA results table for newly-synthesized compounds **3**–**39**	S50
Table S5. PMI ratio calculated for newly-synthesized compounds **3**–**39**	S51

Table S1. Reaction conditions for KA[2]

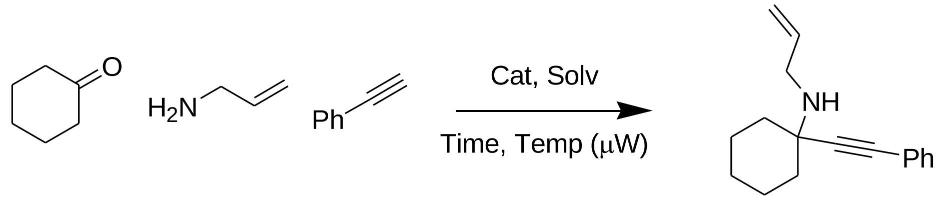

Entry	Catalyst	Solvent	Time	Temperature, °C	Yield, %*
**1**	**CuI**	**-**	**2 h**	**100**	**79 (82)****
**2**	CuI	toluene	2 h	100	50
**3**	CuI	MeOH	2 h	100	62
**4**	CuI	THF	2 h	100	43
**5**	CuBr	–	2 h	100	72
**6**	$CuBr_2$	–	2 h	100	35
**7**	AgOTf	–	2 h	100	8
**8**	CuI	–	1 h	100	73
**9**	CuI	–	0.5 h	150	11
**10**	CuI	–	4 h	60	28

* Measured by HPLC using a calibration curve (see Figure) and linear fitting of absorbance at λ = 223 nm vs concentration in the range 0.2–1.25 mg/mL ($y = 122.2x - 0.017$, $R^2 = 0.996$)

** In parentheses, the isolated yield

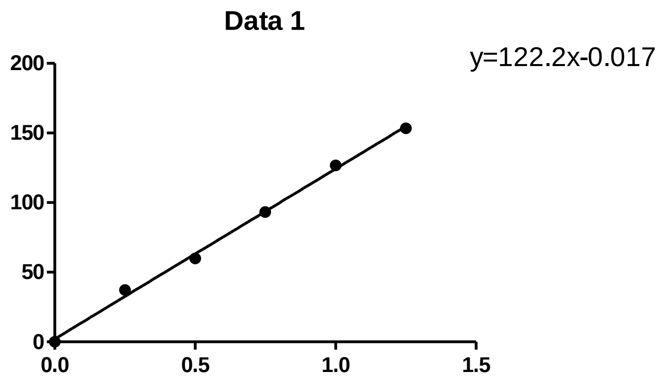

Reverse phase HPLC analyses were performed on an Alltima C18 column, 3 um, 50 mm x 4.6 mm, using water/acetonitrile (MeCN) eluent buffered with 0.1% (v/v) TFA with gradient 25 to 95% MeCN/5’, then 95% MeCN/2’; RT of the product at 2.26 min.

General. Melting points are uncorrected. NMR spectra were recorded on a Varian Mercury 400 (400 MHz, ^{1}H; 100 MHz, ^{13}C), Varian Inova (400 MHz, ^{1}H; 100 MHz, ^{13}C) or on a Varian Gemini 200 (50 MHz, ^{13}C). The chemical shift values (δ) and coupling constants (J) are expressed in parts per million (ppm) and hertz (Hz), respectively. Chemical shifts were referenced to residual non deuterated solvent ($CHCl_3$ ^{1}H: δ = 7.26 ppm, ^{13}C: δ = 77.0 ppm). Flash column chromatography (FCC) purifications were performed manually using glass columns with Merck silica gel (0.040–0.063 mm). TLC analyses were performed on Merck silica gel 60 F254 plates. Elemental analyses were performed on a Perkin Elmer C, H, N analyzer. ESI–MS spectra were recorded on a Thermo Scientific LCQ fleet ion-trap double quadrupole mass spectrometer using electrospray (ES^{+}) ionization techniques. Microwave reactions were carried out on an Automated single-mode microwave synthesizer (Initiator^{TM} Sixty, Biotage AB) using sealed reaction vessels and built-in internal pressure and temperature sensors. All commercially available reagents and solvents were used as received, unless otherwise specified.

General Procedure (A) for the KA^2 coupling reaction. CuI (0.2 equiv) was added in a dry sealed vial for microwave synthesis under a nitrogen flow. Then, ketone (1 equiv), alkyne (1.2 equiv) and amine (1.2 equiv) were successively added under a nitrogen flow, and the mixture was heated under microwave irradiation to 100 °C for 2 h. Then, EtOAc was added and the organic phase was washed with 5% NH_4OH (3 x 20 mL) and brine. The organic phase was dried with Na_2SO_4 and concentrated under reduced pressure. The crude product was purified by Flash Chromatography using the indicated solvent mixture as eluent.

General Procedure (B) for the Amine Protection. The KA^2 product was dissolved in pyridine (2 mL/mmol) and acetic anhydride (4 mL/mmol) was added dropwise to the reaction mixture at 0 °C and. Then, the reaction mixture was heated to 40 °C for 16 h, followed by EtOAc addition. The organic phase was washed with 1M HCl (3 x 20 mL), satd. Na_2CO_3 (3 x 20 mL) and brine. The

organic phase was dried over Na_2SO_4 and concentrated under reduced pressure. The crude product was purified by Flash Chromatography using the indicated solvent mixture as eluent.

General procedure for the Pauson-Khand (C) reaction. In a dry round bottom flask under a nitrogen flow $Co_2(CO)_8$ (0.1 equiv), *N*, *N*, *N*', *N*'-tetramethylthiourea (0.6 equiv) and a solution of the enyne compound (1 equiv) were successively added in dry toluene (20 mL/mmol). Then, the reaction mixture was kept under a CO atmosphere and stirred at 70 °C until disappearance of the starting material as monitored by TLC. Then, the mixture was filtered on Celite and concentrated under reduced pressure. The crude product was purified by Flash Chromatography using the indicated solvent mixture as eluent.

***N*-Allyl-1-(phenylethynyl)cyclohexanamine (3).** Compound **3** was obtained following the general procedure (A) using cyclohexanone (235 µL, 2.28 mmol), phenylacetylene (300 µL, 2.73 mmol), CuI (86 mg) and allylamine (200 µL, 2.68 mmol). The crude product was purified by flash chromatography with 3:1 hexane-EtOAc as eluent, to give **3** (446 mg) as a yellow oil in 82% yield. ^{1}H NMR (400 MHz, $CDCl_3$) δ 7.47 – 7.40 (m, 2H), 7.35 – 7.27 (m, 3H), 6.00 (ddt, J = 16.3, 10.2, 6.1 Hz, 1H), 5.23 (dq, J = 17.2, 1.6 Hz, 1H), 5.09 (dd, J = 10.2, 1.6 Hz, 1H), 3.46 (d, J = 6.1 Hz, 2H), 1.96 (d, J = 13.0 Hz, 2H), 1.82 – 1.57 (m, 5H), 1.57 – 1.38 (m, 4H) ppm. ^{13}C NMR (100 MHz, $CDCl_3$) δ 137.3, 131.6, 128.2, 127.7, 123.6, 115.7, 93.2, 84.8, 55.0, 46.3, 38.2, 25.9, 23.1 ppm. MS (ESI) *m/z* (%): 240.12 [(M + H)$^+$, 100]. Anal. Calcd. for $C_{17}H_{21}N$: C, 85.30; H, 8.84; N, 5.85. Found: C, 85.75; H, 8.91; N, 5.79.

***N*-Allyl-*N*-(1-(phenylethynyl)cyclohexyl)acetamide (4).** Compound **4** was obtained following the general procedure (B) using compound **3** (446 mg, 1.86 mmol), pyridine (3.7 mL, 3.99 mmol) and acetic anhydride (7.5 mL). The crude product was purified by flash chromatography with 3:1 hexane-EtOAc as eluent, to give **4** (318 mg) as a yellow oil in 61% yield. ^{1}H NMR (400 MHz, $CDCl_3$) δ 7.44 – 7.39 (m, 2H), 7.35 – 7.26 (m, 3H), 6.00 – 5.85 (m, 1H), 5.28 (dd, J = 17.2, 1.1 Hz, 1H), 5.21 (dd, J = 10.4, 1.1 Hz, 1H), 4.31 – 4.20 (m, 2H), 2.55 (br s, 2H), 2.15 (s, 3H), 2.00 (d, J =

11.8 Hz, 2H), 1.88 – 1.59 (m, 6H) ppm. ^{13}C NMR (100 MHz, $CDCl_3$) δ 171.4, 135.4, 131.5, 128.2, 128.1, 123.2, 115.9, 90.5, 87.7, 49.9, 34.7, 25.2, 24.9, 24.1 ppm. MS (ESI) *m*/*z* (%): 304.12 [(M + Na)$^+$, 100]. Anal. Calcd. for $C_{19}H_{23}NO$: C, 81.10; H, 8.24; N, 4.98. Found: C, 81.19; H, 8.30; N, 4.89.

2’-Acetyl-6’-phenyl-3a’,4’-dihydro-2’H-spiro[cyclohexane-1,1’-cyclopenta[c]pyrrol]-5’(3’H)-one (5). Compound **5** was obtained following the general procedure (C) using compound **4** (318 mg, 1.13 mmol), toluene (23 mL), $Co_2(CO)_8$ (38 mg, 0.11 mmol), *N*, *N*, *N*’, *N*’-tetramethylthiourea (87 mg, 0.66 mmol). The reaction mixture was heated for 7 h and the crude product was purified by flash chromatography with EtOAc as eluent to give **5** (240 mg) as a white solid in 73% yield. Mp = 177−180 °C. ^{1}H NMR (400 MHz, $CDCl_3$) δ 7.45 – 7.31 (m, 3H), 7.11 (dt, *J* = 4.1, 2.3 Hz, 2H), 4.02 (t, *J* = 9.0 Hz, 1H), 3.50 - 3.43 (m, 1H), 3.22 – 3.08 (m, 1H), 2.97 (td, *J* = 13.7, 6.0 Hz, 1H), 2.85 – 2.73 (m, 1H), 2.77 (dd, *J* = 18.3, 6.6 Hz, 1H), 2.28 (dd, *J* = 18.3, 3.3 Hz, 1H), 2.10 (s, 3H), 1.67 – 1.53 (m, 4H), 1.30 – 1.07 (m, 4H) ppm. ^{13}C NMR (50 MHz, $CDCl_3$) δ 207.1, 177.8, 168.9, 139.1, 129.2, 128.7, 79.3, 65.1, 53.2, 39.6, 38.6, 31.6, 29.1, 28.5, 24.8 ppm. MS (ESI) *m*/*z* (%): 332.06 [(M + H)$^+$, 100]. Anal. Calcd. for $C_{20}H_{23}NO_2$: C, 77.64; H, 7.49; N, 4.53. Found: C, 78.01; H, 7.54; N, 4.46.

***N*-Allyl-1-(thiophen-3-ylethynyl)cyclohexanamine (7).** Compound **7** was obtained following the general procedure (A) using cyclohexanone (88 µL, 0.84 mmol), 3-ethynylthiophene (100 µL, 1.01 mmol), CuI (30 mg, 0.17 mmol) and allylamine (75 µL, 1.01 mmol). The crude product was purified by flash chromatography with 2:1 hexane/EtOAc as eluent to give **7** (152 mg) as a yellow oil in 74% yield. ^{1}H NMR (400 MHz, $CDCl_3$) δ 7.38 (dd, *J* = 3.0, 1.2 Hz, 1H), 7.25 – 7.24 (m, 1H), 7.09 (dd, *J* = 5.0, 1.2 Hz, 1H), 6.07 – 5.92 (m, 1H), 5.22 (dq, *J* = 17.2, 1.6 Hz, 1H), 5.09 (ddd, *J* = 10.2, 2.8, 1.3 Hz, 1H), 3.46 – 3.41 (m, 2H), 1.94 (d, *J* = 12.7 Hz, 2H), 1.70 - 1.63 (m, 6H), 1.51 – 1.40 (m, 3H) ppm. ^{13}C NMR (100 MHz, $CDCl_3$) δ 137.3, 130.1, 127.8, 125.1, 122.6, 115.6, 93.0,

79.6, 55.0, 46.3, 38.2, 25.9, 23.0 ppm. MS (ESI) *m*/*z* (%): 268.22 [(M + Na)$^+$, 100]. Anal. Calcd. for $C_{15}H_{19}NS$: C, 73.42; H, 7.80; N, 5.71. Found: C, 73.81; H, 7.99; N, 5.64.

***N*-Allyl-*N*-(1-(thiophen-2-ylethynyl)cyclohexyl)acetamide (8).** Compound **8** was obtained following the general procedure (B) using compound **7** (150 mg, 0.61 mmol), pyridine (1.1 mL, 1.36 mmol) and acetic anhydride (2.2 mL). The crude product was purified by flash chromatography with 2:1 hexane/EtOAc as eluent to give **8** (137 mg) as a yellow oil in 78% yield. ^{1}H NMR (400 MHz, $CDCl_3$) δ 7.42 – 7.37 (m, 1H), 7.25 (ddd, *J* = 5.1, 2.9, 0.9 Hz, 1H), 7.09 (dd, *J* = 4.9, 1.1 Hz, 1H), 5.97 – 5.84 (m, 1H), 5.27 (d, *J* = 17.2 Hz, 1H), 5.20 (d, *J* = 10.5 Hz, 1H), 4.25 – 4.18 (m, 2H), 2.15 (s, 3H), 2.01 (d, *J* = 12.1 Hz, 2H), 1.82 – 1.57 (m, 8H) ppm. ^{13}C NMR (100 MHz, $CDCl_3$) δ 171.4, 135.4, 129.8, 128.2, 125.5, 122.1, 115.9, 90.0, 82.7, 61.6, 49.7, 34.8, 25.2, 24.9, 24.0 ppm. MS (ESI) *m*/*z* (%): 300.20 [(M + H)$^+$, 100]. Anal. Calcd. for $C_{17}H_{21}NOS$: C, 71.04; H, 7.36; N, 4.87. Found: C, 71.10; H, 7.43; N, 4.80.

2’-Acetyl-6’-(thiophen-3-yl)-3a’,4’-dihydro-2’*H*-spiro[cyclohexane-1,1’-cyclopenta[*c*]pyrrol]-5’(3’H)-one (9). Compound **9** was obtained following the general procedure (C) using compound **8** (130 mg, 0.45 mmol), toluene (9 mL), $Co_2(CO)_8$ (15 mg, 0.04 mmol), *N*,*N*,*N*’,*N*’-tetramethylthiourea (40 mg, 0.3 mmol). The reaction mixture was heated for 4 h and the crude product was purified by flash chromatography with EtOAc as eluent to give **9** (96 mg) as a white solid in 68% yield. Mp = 183−186 °C. ^{1}H NMR (400 MHz, $CDCl_3$) δ 7.36 (dd, *J* = 4.9, 3.0 Hz, 1H), 7.09 (dd, *J* = 2.8, 1.0 Hz, 1H), 6.89 (dd, *J* = 4.9, 1.0 Hz, 1H), 4.00 (dd, *J* = 19.4, 10.4 Hz, 1H), 3.50 – 3.37 (m, 1H), 3.22 – 3.06 (m, 1H), 2.97 (td, *J* = 13.4, 5.5 Hz, 1H), 2.83 – 2.69 (m, 2H), 2.31 – 2.22 (m, 1H), 2.08 (s, 3H), 1.59 (t, *J* = 14.9 Hz, 4H), 1.42 – 1.06 (m, 6H) ppm. ^{13}C NMR (100 MHz, $CDCl_3$) δ 207.7, 181.22, 168.8, 134.1, 132.3, 128.7, 126.0, 124.7, 67.8, 52.9, 39.8, 38.1, 32.7, 28.9, 25.3, 24.1, 23.4, 21.9 ppm. MS (ESI) *m*/*z* (%): 316.14 [(M + H)$^+$, 100]. Anal. Calcd. for $C_{18}H_{21}NO_2S$: C, 68.54; H, 6.71; N, 4.44. Found: C, 68.71; H, 6.76; N, 4.40.

***N*-Allyl-1-(phenylethynyl)cyclopentanamine (13).** Compound **13** was obtained following the general procedure (A) using cyclopentanone (100 µL, 1.13 mmol), phenylacetylene (150 µL, 1.36 mmol), CuI (43 mg, 0.23 mmol) and allylamine (100 µL, 1.36 mmol). The crude product was purified by flash chromatography with 3:1 hexane/EtOAc as eluent to give **13** (156 mg) as a yellow oil in 61% yield. ^{1}H NMR (400 MHz,$CDCl_3$) *δ* 7.40 (dd, *J* = 6.5, 3.2 Hz, 2H), 7.31 – 7.27 (m, 3H), 6.05 – 5.95 (m, 1H), 5.23 (dd, *J* = 17.2, 1.6 Hz, 1H), 5.09 (dd, *J* = 10.2, 1.3 Hz, 1H) 3.44 (d, *J* = 6.0 Hz, 2H), 2.04 (dd, *J* = 13.9, 7.4 Hz, 2H), 1.90 – 1.73 (m, 6H). ^{13}C NMR (100 MHz, $CDCl_3$) *δ* 137.6, 136.1, 131.6, 129.7, 128.3, 128.2, 126.7, 116.5, 89.9, 84.9, 49.9, 48.3, 42.1, 40.6, 23.8 ppm. MS (ESI) *m/z* (%): 226.19 [$(M + H)^+$, 100]. Anal. Calcd. for $C_{16}H_{19}N$: C, 85.28; H, 8.50; N, 6.22. Found: C, 85.47; H, 8.59; N, 6.14.

***N*-Allyl-*N*-(1-(phenylethynyl)cyclopentyl)acetamide (14).** Compound **14** was obtained following the general procedure (B) using compound **14** (150 mg, 0.67 mmol), pyridine (1.34 mL, 1.66 mmol) and acetic anhydride (2.68 mL). The crude product was purified by flash chromatography with 4:1 hexane-EtOAc as eluent to give **14** (100 mg) as a yellow oil in 56% yield. ^{1}H NMR (400 MHz, $CDCl_3$) *δ* 7.40 – 7.35 (m, 2H), 7.35 – 7.26 (m, 3H), 6.00 – 5.88 (m, 1H), 5.32 – 5.17 (m, 2H), 4.20 – 4.11 (m, 2H), 2.47 – 2.42 (m, 2H), 2.19 – 2.09 (m, 3H), 2.09 (s, 3H), 1.92 – 1.76 (m, 3H) ppm. ^{13}C NMR (50 MHz, $CDCl_3$) *δ* 171.8, 151.1, 135.3, 134.7, 131.6, 131.5, 129.7, 128.3, 128.2, 127.9, 126.8, 92.9, 82.9, 63.9, 49.9, 40.2, 29.7, 23.3 ppm. MS (ESI) *m/z* (%): 290.23 [$(M + Na)^+$, 100]. Anal. Calcd. for $C_{18}H_{21}NO$: C, 80.86; H, 7.92; N, 5.24. Found: C, 81.17; H, 8.01; N, 5.16.

2'-Acetyl-6'-phenyl-3a',4'-dihydro-2'*H*-spiro[cyclopentane-1,1'-cyclopenta[*c*]pyrrol]-5'(3'*H*)-one (15). Compound **15** was obtained following the general procedure (C) using compound **14** (80 mg, 0.29 mmol), $Co_2(CO)_8$ (10 mg, 0.03 mmol), *N*,*N*,*N*',*N*'-tetramethylthiourea (24 mg, 0.18 mmol). The reaction mixture was heated for 6 h and the crude product was purified by flash chromatography with EtOAc as eluent to give **15** (61 mg) as a yellow oil in 72% yield. ^{1}H NMR (400 MHz, $CDCl_3$) *δ* 7.48 – 7.29 (m, 3H), 7.14 (dd, *J* = 7.8, 1.6 Hz, 2H), 4.03 (t, *J* = 9.1 Hz, 1H),

3.50 – 3.39 (m, 1H), 3.17 (dd, *J* = 11.2, 9.6 Hz, 1H), 2.81 (dd, *J* = 18.0, 6.6 Hz, 1H), 2.58 (dt, *J* = 13.2, 8.0 Hz, 1H), 2.34 (dd, *J* = 18.0, 3.6 Hz, 1H), 2.24 – 2.13 (m, 2H), 2.08 (s, 3H), 2.01 – 1.88 (m, 2H), 1.75 (ddd, *J* = 13.5, 9.8, 5.6 Hz, 1H), 1.71 – 1.58 (m, 3H) ppm. ^{13}C NMR (100 MHz, $CDCl_3$) δ 207.1, 183.3, 168.2, 136.0, 129.6, 128.3, 72.5, 52.8, 40.9, 40.2, 39.1, 34.9, 27.3, 27.1, 24.5 ppm. MS (ESI) *m*/*z* (%): 318.14 [$(M + Na)^+$, 100]. Anal. Calcd. for $C_{19}H_{21}NO_2$: C, 77.26; H, 7.17; N, 4.74. Found: C, 77.68; H, 7.26; N, 4.69.

***N*-Allyl-4-methyl-1-(phenylethynyl)cyclohexanamine (17).** Compound **17** was obtained following the general procedure (A) using 4- methylcyclohexanone (200 µL, 1.63 mmol), phenylacetylene (214 µL, 1.95 mmol), CuI (60 mg, 0.32 mmol) and allylamine (145 µL, 1.95 mmol). The crude product was purified by flash chromatography with 3:1 hexane/EtOAc as eluent to give **17** (338 mg) as a yellow oil in 82% yield. ^{1}H NMR (400 MHz, $CDCl_3$) δ 7.46 – 7.40 (m, 2H), 7.33 – 7.27 (m, 3H), 6.03 – 5.96 (m, 1H), 5.27 – 5.18 (m, 1H), 5.09 (dd, *J* = 10.2, 1.6 Hz, 1H), 3.47 (d, *J* = 6.1 Hz, 2H), 2.02 (dd, *J* = 26.2, 8.1 Hz, 2H), 1.50 – 1.36 (m, 7H), 0.93 (d, *J* = 5.4 Hz, 3H) ppm. ^{13}C NMR (100 MHz, $CDCl_3$) δ 137.3, 131.6, 128.2, 127.8, 123.6, 115.7, 92.9, 85.1, 55.1, 46.53, 38.3, 32.4, 31.9, 22.2 ppm. MS (ESI) *m*/*z* (%): 276.15 [$(M + Na)^+$, 100]. Anal. Calcd. for $C_{18}H_{23}NO$: C, 85.32; H, 9.15; N, 5.53. Found: C, 85.78; H, 9.24; N, 5.47.

***N*-Allyl-*N*-(4-methyl-1-(phenylethynyl)cyclohexyl)acetamide (18).** Compound **18** was obtained following the general procedure (B) using compound **17** (230 mg, 0.9 mmol), pyridine (1.8 mL, 2.23 mmol) and acetic anhydride (3.6 mL). The crude product was purified by flash chromatography with 3:1 hexane/EtOAc as eluent to give **18** (180 mg) as a yellow oil in 68% yield. ^{1}H NMR (400 MHz, $CDCl_3$) δ 7.42 – 7.40 (m, 2H), 7.34 – 7.28 (m, 3H), 5.93 (ddd, *J* = 15.2, 9.8, 4.6 Hz, 1H), 5.28 (d, *J* = 15.2 Hz, 1H), 5.21 (d, *J* = 10.5 Hz, 1H), 4.28 – 4.23 (m, 2H), 2.16 (s, 3H), 2.01 (d, *J* = 12.1 Hz, 2H), 1.73 – 1.40 (m, 7H), 0.91 (d, *J* = 5.3 Hz, 3H) ppm. ^{13}C NMR (100 MHz, $CDCl_3$) δ 171.4, 135.5, 131.4, 128.2, 128.1, 123.2, 115.9, 90.6, 87.7, 34.5, 32.6, 31.4, 25.2, 22.3

ppm. MS (ESI) *m*/*z*(%): 296.20 [(M + H)$^+$, 100]. Anal. Calcd. for $C_{20}H_{25}NO$: C, 81.31; H, 8.53; N, 4.74. Found: C, 81.89; H, 8.61; N, 4.68.

2'-Acetyl-4-methyl-6'-phenyl-3a',4'-dihydro-2'*H*-spiro[cyclohexane-1,1'-cyclopenta[*c*]pyrrol]-5'(3'*H*)-one (19). Compound **19** was obtained following the general procedure (C) using compound **18** (180 mg, 0.62 mmol), $Co_2(CO)_8$ (20 mg, 0.06 mmol), *N*,*N*,*N*',*N*'-tetramethylthiourea (48 mg, 0.36 mmol). The reaction mixture was heated for 8 h and the crude product was purified by flash chromatography with 3:1 hexane-EtOAc as eluent to give **19** (144 mg) as a white solid in 72% yield. Mp = 176–177 °C. ^{1}H NMR (400 MHz, $CDCl_3$) δ 7.38 (d, *J* = 6.0 Hz, 3H), 7.17 – 7.07 (m, 2H), 4.02 (td, *J* = 9.2, 3.3 Hz, 1H), 3.56 – 3.41 (m, 1H), 3.15 (t, *J* = 10.5 Hz, 1H), 3.05 (td, *J* = 13.7, 6.0 Hz, 1H), 2.97 – 2.83 (m, 1H), 2.83 – 2.73 (m, 1H), 2.28 (dd, *J* = 18.3, 1.9 Hz, 1H), 2.10 (d, *J* = 7.0 Hz, 3H), 1.65 – 1.19 (m, 8H), 0.51 (d, *J* = 6.5 Hz, 1H) ppm. ^{13}C NMR (100 MHz, $CDCl_3$) δ 207.9, 179.8, 168.8, 168.7, 138.8, 133.2, 129.4, 129.2, 128.4, 128.2, 67.7, 53.0, 39.8, 38.3, 38.1, 28.6, 28.5, 27.7, 26.3, 21.9 ppm. MS (ESI) *m*/*z* (%): 346.25 [(M + Na)$^+$, 100]. Anal. Calcd. for $C_{21}H_{25}NO_2$: C, 77.98; H, 7.79; N, 4.33. Found: C, 78.32; H, 7.86; N, 4.26.

***N*-*tert*-Butyl 4-(allylamino)-4-(phenylethynyl)piperidine-1-carboxylate (24).** Compound **24** was obtained following the general procedure (A) using *tert*-butyl 4-oxopiperidine-1-carboxylate (220 mg, 1.11 mmol), phenylacetylene (146 µL, 1.33 mmol), CuI (42 mg, 0.22 mmol) and allylamine (100 µL, 1.33 mmol). The crude product was purified by flash chromatography with 1:1 hexane/EtOAc as eluent to give **24** (313 mg) as a yellow oil in 83% yield. ^{1}H NMR (400 MHz, $CDCl_3$) δ 7.40 (ddd, *J* = 4.2, 2.9, 1.7 Hz, 2H), 7.34 – 7.26 (m, 3H), 6.00 – 5.93 (m, 1H), 5.22 (dd, *J* = 17.2, 1.6 Hz, 1H), 5.09 (dd, *J* = 10.2, 1.4 Hz, 1H), 3.97 (pd, *J* = 24.4 Hz, 2H), 3.43 (d, *J* = 6.0 Hz, 2H), 3.18 (ps, 2H), 1.88 (d, *J* = 12.0 Hz, 2H), 1.59 (td, *J* = 12.7, 3.9 Hz, 2H), 1.44 (s, 9H) ppm. ^{13}C NMR (50 MHz, $CDCl_3$) δ 154.8, 137.0, 131.7, 128.3, 128.2, 123.0, 115.9, 91.3, 85.8, 79.4, 53.7, 46.3, 40.8, 37.5, 28.5 ppm. MS (ESI) *m*/*z* (%): 341.22 [(M + H)$^+$, 100]. Anal. Calcd. for $C_{21}H_{28}N_2O_2$: C, 74.08; H, 8.29; N, 8.23. Found: C, 74.53; H, 8.37; N, 8.15.

***N-tert*-Butyl 4-(*N*-allylacetamido)-4-(phenylethynyl)piperidine-1-carboxylate (25).** Compound **25** was obtained following the general procedure (B) using compound **24** (300 mg, 0.88 mmol), pyridine (1.66 mL, 2.06 mmol) and acetic anhydride (3.32 mL). The crude product was purified by flash chromatography with 1:1 hexane/EtOAc as eluent to give **25** (211 mg) as a yellow oil in 63% yield. ^{1}H NMR (400 MHz, $CDCl_3$) δ 7.45 – 7.39 (m, 2H), 7.34 – 7.28 (m, 3H), 6.09 – 5.80 (m, 1H), 5.31 (dd, J = 17.2, 1.0 Hz, 1H), 5.24 (dd, J = 10.5, 1.1 Hz, 1H), 4.24 – 4.21 (m, 2H), 4.17 - 4.06 (m, 2H), 3.17 (ps, 2H), 2.57 (td, J = 12.6, 4.4 Hz, 2H), 2.15 (s, 3H), 2.05 (d, J = 11.6 Hz, 2H), 1.46 (s, 9H) ppm. ^{13}C NMR (50 MHz, $CDCl_3$) δ 171.5, 152.3, 135.1, 131.6, 128.48, 128.3, 122.6, 116.3, 92.5, 88.2, 79.6, 59.6, 49.6, 41.6, 34.5, 28.5, 24.9 ppm. MS (ESI) *m*/*z* (%): 383.25 [(M + H)$^{+}$, 100]. Anal. Calcd. for $C_{23}H_{30}N_2O_3$: C, 72.22; H, 7.91; N, 7.32. Found: C, 72.58; H, 7.99; N, 7.24.

***N-tert*-Butyl 2-acetyl-5-oxo-6-phenyl-3,3a,4,5-tetrahydro-2*H*-spiro[cyclopenta[*c*]pyrrole-1,4'-piperidine]-1'-carboxylate (26).** Compound **26** was obtained following the general procedure (C) using compound **26** (203 mg, 0.53 mmol), toluene (10 mL), $Co_2(CO)_8$ (17 mg, 0.05 mmol), *N*,*N*,*N*',*N*'-tetramethylthiourea (40 mg, 0.3 mmol). The reaction mixture was heated for 3 h and the crude product was purified by flash chromatography with 3:1 hexane/EtOAc as eluent to give **26** (171 mg) as a yellow oil in 79% yield. ^{1}H NMR (400 MHz, $CDCl_3$) δ 7.39 – 7.36 (m, 3H), 7.15 – 7.06 (m, 1H), 4.04 (t, J = 9.1 Hz, 1H), 3.89 – 3.68 (m, 1H), 3.55-3.46 (m, 2H), 3.24 – 3.12 (m, 1H), 2.84 (m, 3H), 2.80 (dd, J = 18.3, 6.7 Hz, 1H), 2.34 – 2.29 (m, 1H), 2.19 (s, 3H), 2.09 (s, 1H), 1.85 – 1.56 (m, 3H), 1.39 (d, J = 17.3 Hz, 9H) ppm. ^{13}C NMR (50 MHz, $CDCl_3$) δ 207.1, 177.8, 168.9, 139.1, 129.2, 128.7, 79.3, 65.1, 53.1, 39.6, 38.6, 31.6, 29.1, 28.5, 24.8 ppm. MS (ESI) *m*/*z* (%): 433.15 [(M + Na)$^{+}$, 100]. Anal. Calcd. for $C_{24}H_{30}N_2O_4$: C, 70.22; H, 7.37; N, 6.82. Found: C, 70.80; H, 7.45; N, 6.75.

***N-tert*-Butyl 4-(allylamino)-4-(thiophen-3-ylethynyl)piperidine-1-carboxylate (27).** Compound **27** was obtained following the general procedure (A) using *N-tert*-butyl 4-oxopiperidine-1-carboxylate (218 mg, 1.1 mmol), 3-ethynylthiophene (130 µL, 1.32 mmol), CuI (41 mg, 0.22

mmol) and allylamine (100 µL, 1.32 mmol). The crude product was purified by flash chromatography with 1:1 hexane-diethyl ether as eluent to give **27** (235 mg) as a white solid in 62% yield. Mp = 153–156 °C. ^{1}H NMR (400 MHz, $CDCl_3$) *δ* 7.40 (dd, *J* = 3.0, 1.1 Hz, 1H), 7.27 – 7.25 (m, 1H), 7.08 (dd, *J* = 5.0, 1.1 Hz, 1H), 5.97 (ddt, *J* = 16.3, 10.2, 6.0 Hz, 1H), 5.23 (dq, *J* = 17.2, 1.5 Hz, 1H), 5.10 (dd, *J* = 10.2, 1.3 Hz, 1H), 3.96 (ps, 2H), 3.47 – 3.38 (m, 3H), 3.21 - 3.16 (m, 2H), 1.89 (d, *J* = 12.5 Hz, 2H), 1.60 (td, *J* = 12.4, 4.0 Hz, 2H), 1.45 (s, 9H) ppm. ^{13}C NMR (100 MHz, $CDCl_3$) *δ* 154.7, 136.9, 129.9, 128.4, 125.3, 121.9, 115.9, 90.7, 80.7, 79.5, 53.7, 46.2, 28.4 ppm. MS (ESI) *m/z* (%): 347.18 [$(M + H)^+$, 100]. Anal. Calcd. for $C_{19}H_{26}N_2O_2S$: C, 65.86; H, 7.56; N, 8.08. Found: C, 66.01; H, 7.62; N, 8.00.

***N*-*tert*-Butyl 4-(*N*-allylacetamido)-4-(thiophen-3-ylethynyl)piperidine-1-carboxylate (28).** Compound **28** was obtained following the general procedure (B) using compound **27** (221 mg, 0.63 mml), pyridine (1.26 mL, 1.56 mmol) and acetic anhydride (2.52 mL). The crude product was purified by flash chromatography with 2:1 hexane-diethyl ether as eluent to give **28** (153 mg) as a yellow oil in 63 % yield. ^{1}H NMR (400 MHz, $CDCl_3$) *δ* 7.41 (d, *J* = 1.7 Hz, 1H), 7.27 7.25 (m, 1H), 7.08 (d, *J* = 4.9 Hz, 1H), 5.89 (ddd, *J* = 15.0, 9.9, 4.5 Hz, 1H), 5.29 (d, *J* = 17.3 Hz, 1H), 5.23 (d, *J* = 10.5 Hz, 1H), 4.19 – 4.05 (m, 4H), 3.14 (ps, 2H), 2.55 – 2.44 (m, 2H), 2.14 (s, 3H), 2.06 (d, *J* = 11.8 Hz, 2H), 1.45 (s, 9H) ppm. ^{13}C NMR (100 MHz, $CDCl_3$) *δ* 171.5, 154.6, 134.9, 129.8, 128.8, 125.4, 121.5, 116.3, 88.1, 83.3, 79.6, 59.4, 49.4, 34.4, 28.4, 24.9 ppm. MS (ESI) *m/z* (%): 411.19 [$(M + Na)^+$, 100]. Anal. Calcd. for $C_{21}H_{28}N_2O_3S$: C, 64.92; H, 7.26; N, 7.21. Found: C, 65.31; H, 7.31; N, 7.17.

***N*-*tert*-Butyl 2-acetyl-5-oxo-6-(thiophen-2-yl)-3,3a,4,5-tetrahydro-2*H*-spiro[cyclopenta[*c*]pyrrole-1,4’-piperidine]-1’-carboxylate (29).** Compound **29** was obtained following the general procedure (C) using compound **28** (149 mg, 0.38 mmol), toluene (7.6 mL) $Co_2(CO)_8$ (14 mg, 0.04 mmol), *N*,*N*,*N*’,*N*’-tetramethylthiourea (32 mg, 0.24 mmol). The reaction mixture was heated for 3 h and the crude product was purified by flash chromatography with EtOAc

as eluent to give **29** (108 mg) as a yellow oil in 68% yield. ^{1}H NMR (400 MHz, $CDCl_3$) δ 7.37 (dd, *J* = 4.9, 3.0 Hz, 1H), 7.09 (dd, *J* = 2.8, 1.0 Hz , 1H), 6.90 (dd, *J* = 4.9, 1.0 Hz, 1H), 4.15 (dd, *J* = 17.0, 7.9 Hz, 1H), 3.99 – 3.79 (m, 1H), 3.72 – 3.56 (m, 1H), 3.29 (t, *J* = 10.2 Hz, 1H), 2.90 (dd, *J* = 18.3, 6.7 Hz, 1H), 2.79 – 2.72 (m, 1H), 2.42 (dd, J = 18.3, 3.2 Hz, 1H), 2.34 – 2.26 (m, 1H), 2.19 (s, 3H), 1.93 – 1.63 (m, 2H), 1.49 (d, *J* = 17.3 Hz, 9H) ppm. ^{13}C NMR (100 MHz, $CDCl_3$) δ 209.7, 183.2, 170.8, 136.1, 134.3, 130.7, 128.0, 126.7, 78.3, 64.1, 52.1, 38.6, 37.6, 30.6, 28.1, 27.5, 23.8 ppm. MS (ESI) *m*/*z* (%): 417.20 [$(M + H)^+$, 100]. Anal. Calcd. for $C_{22}H_{28}N_2O_4S$: C, 63.44; H, 6.78; N, 6.73. Found: C, 63.74; H, 6.85; N, 6.68.

***N*-*tert*-Butyl 4-(*N*-allylbenzamido)-4-(phenylethynyl)piperidine-1-carboxylate (30).** Compound **24** (540 mg, 1.58 mmol) was dissolved in pyridine (3.15 mL) and the reaction mixture was kept to 0 °C while adding tosyl chloride (780 µL, 9.45 mmol) portionwise. Then, the reaction mixture was heated to 40 °C for 16 h. Then, EtOAc was added and the organic phase was washed with 1M HCl (3 × 20 mL), satd. Na_2CO_3 (3 × 20 mL) and brine. The organic phase was dried with Na_2SO_4 and concentrated under reduced pressure. The crude product was purified by Flash Chromatography using a mixture of 4:1 hexane/diethyl ether, to give pure **30** (448 mg) as a pale yellow oil in a yield of 68%. ^{1}H NMR (400 MHz, $CDCl_3$) δ 7.44 (dd, *J* = 6.7, 3.1 Hz, 2H), 7.41 – 7.35 (m, 5H), 7.32 (dd, *J* = 5.0, 1.8 Hz, 3H), 5.86 (ddt, *J* = 15.9, 10.4, 5.1 Hz, 1H), 5.17 (ps, 2H), 5.14 (dd, *J* = 8.3, 1.1 Hz, 2H), 4.16 (d, *J* = 5.1 Hz, 2H), 3.33 – 3.14 (m, 2H), 2.69 (td, *J* = 12.5, 4.5 Hz, 2H), 2.20 (d, *J* = 12.2 Hz, 2H), 1.47 (s, 9H) ppm. ^{13}C NMR (100 MHz, $CDCl_3$) mixture of rotamers δ 173.1, 154.7, 138.4, 136.1, 131.6, 129.4, 128.4, 128.3, 128.3, 126.4, 122.6, 116.8, 88.6, 79.6, 59.3, 51.2, 41.2, 34.2, 28.5, 28.4 ppm. MS (ESI) *m*/*z* (%): 467.30 [$(M + Na)^+$, 100]. Anal. Calcd. for $C_{28}H_{32}N_2O_3$: C, 75.65; H, 7.26; N, 6.30. Found: C, 75.89; H, 7.34; N, 6.19.

***N*-*tert*-Butyl 2-benzoyl-5-oxo-6-phenyl-3,3a,4,5-tetrahydro-2*H*-spiro[cyclopenta[*c*]pyrrole-1,4'-piperidine]-1'-carboxylate (31).** Compound **31** was obtained following the general procedure (C) using compound **30** (430 mg, 0.96 mmol), toluene (19.2 mL), $Co_2(CO)_8$ (34 mg, 0.1 mmol), *N*,

N, *N*', *N*'-tetramethylthiourea (80 mg, 0,6 mmol). The reaction mixture was heated for 3 h and the crude product was purified by flash chromatography with 3:1 hexane/EtOAc as eluent to give **31** (353 mg) as a white solid in 78% of yield. Mp = 217–219 °C. ^{1}H NMR (400 MHz, $CDCl_3$) δ 7.41 (ps, 8H), 7.14 (d, *J* = 6.8 Hz, 2H), 4.06 - 3.87 (m, 1H), 3.83 (dd, *J* = 9.9, 8.4 Hz, 1H), 3.67 – 3.29 (m, 1H), 3.21 (t, *J*= 10.5, 1H), 2.96 – 2.79 (m, 2H), 2.72 (dd, *J* = 18.4, 6.6 Hz, 1H), 2.24 (dd, *J* = 18.5, 3.1 Hz, 1H), 2.16 – 2.05 (m, 1H), 1.92 – 1.77 (m 1H), 1.73 – 1.66 (m, 2H),1.41 (d, *J* = 21.8 Hz, 1H) ppm. ^{13}C NMR (100 MHz, $CDCl_3$) mixture of rotamers δ 207.3, 193.5, 179.5, 169.6, 154.7, 146.7, 137.8, 129.9, 129.1, 128.8, 128.6, 126.3, 79.4, 65.6, 55.3, 38.2, 31.6, 28.4 ppm. MS (ESI) *m*/*z* (%): 473.26 [$(M + H)^+$, 100]. Anal. Calcd. for $C_{29}H_{32}N_2O_4$: C, 73.70; H, 6.83; N, 5.93. Found: C, 74.11; H, 6.92; N, 5.88.

***N*-*tert*-Butyl 4-(*N*-allyl-4-methylphenylsulfonamido)-4-(phenylethynyl)piperidine-1-carboxylate (32).** Compound **24** (360 mg, 1.05 mmol) was dissolved in pyridine (2.1 mL) and while maintaining the reaction mixture at 0 °C tosyl chloride (1200 mg, 6.30 mmol) was added portionwise, and the reaction mixture was heated to 40 °C for 16 h. Then, EtOAc was added and the organic phase was washed with 1M HCl (3 × 20 mL), satd. Na_2CO_3 (3 × 20 mL) and brine. The organic phase was dried with Na_2SO_4 and concentrated under reduced pressure. The crude product was purified by flash chromatography using a mixture of 3:1hexane-diethyl ether, to give pure **32** (326 mg) as a yellow oil in a yield of 63%. ^{1}H NMR (400 MHz, $CDCl_3$) δ 7.75 (d, *J* = 8.3 Hz, 1H), 7.33 – 7.24 (m, 2H), 7.23 – 7.14 (m, 2H), 6.04 (dq, *J* = 10.6, 5.6 Hz, 1H), 5.31 (d, *J* = 17.3 Hz, 1H), 5.19 (d, *J* = 10.3 Hz, 1H), 4.19 (ps, 2H), 4.05 (ps, 2H), 3.06 (s, 1H), 2.33 (ps, 4H), 1.45 (s, 9H) ppm. ^{13}C NMR (100 MHz, $CDCl_3$) δ 179.6, 154.4, 143.2, 138.8, 136.8, 131.5, 129.4, 128.6, 128.2, 127.5, 121.9, 116.9, 87.9, 87.1, 79.8, 60.9, 50.3, 28.4, 21.4 ppm. MS (ESI) *m*/*z* (%): 417.20 [$(M + Na)^+$, 100]. Anal. Calcd. for $C_{28}H_{34}N_2O_4S$: C, 67.99; H, 6.93; N, 5.66. Found: C, 68.34; H, 7.02; N, 5.58.

***N*-Allyl-4-(phenylethynyl)-1-tosylpiperidin-4-amine (34).** Compound **34** was obtained following the general procedure (A) using 1-tosylpiperidin-4-one (370 mg, 0.93 mmol), phenylacetylene (122 µL, 1.12 mmol), CuI (35 mg, 0.19 mmol) and allylamine (85 µL 1.12 mmol). The crude product was purified by flash chromatography with 2:1 hexane/EtOAc as eluent to give **34** (259 mg) as a yellow oil in 71% yield. ^{1}H NMR (400 MHz, $CDCl_3$) δ 7.67 (d, J = 8.2 Hz, 2H), 7.33 (d, J = 8.1 Hz, 2H), 7.25 – 7.21 (m, 2H), 7.16 – 7.12 (m, 3H), 5.93 (ddt, J = 16.3, 10.3, 6.0 Hz, 1H), 5.20 (dd, J = 17.2, 1.6 Hz, 1H), 5.08 (dd, J = 10.2, 1.3 Hz, 1H), 3.61 (dt, J = 11.8, 3.7 Hz, 2H), 3.37 (d, J = 6.0 Hz, 2H), 2.81 (td, J = 11.6, 2.6 Hz, 2H), 2.43 (s, 3H), 1.96 (d, J = 12.9 Hz, 2H), 1.82 – 1.73 (m, 2H). ^{13}C NMR (100 MHz, $CDCl_3$) δ 143.4, 136.7, 133.2, 131.4, 129.7, 129.5, 128.2, 128.2, 127.7, 127.6, 122.6, 116.0, 106.0, 90.3, 86.6, 64.4, 52.9, 46.3, 44.5, 43.2, 36.8, 34.4, 21.5 ppm. MS (ESI) *m*/*z* (%): 417.26 [(M + Na)$^+$, 100]. Anal. Calcd. for $C_{23}H_{26}N_2O_2S$: C, 70.02; H, 6.64; N, 7.10. Found: C, 70.42; H, 6.71; N, 7.00.

***N*-Allyl-*N*-(4-(phenylethynyl)-1-tosylpiperidin-4-yl)acetamide (35).** Compound **35** was obtained following the general procedure (B) using compound **34** (259 mg, 0.66 mmol), pyridine (1.32 mL, 1.66 mmol) and acetic anhydride (2.64 mL). The crude product was purified by flash chromatography with 3:1 hexane/EtOAc as eluent to give **35** (150 mg) as a yellow oil in 52% yield. ^{1}H NMR (400 MHz, $CDCl_3$) δ 7.74 – 7.63 (m, 1H), 7.35 – 7.28 (m, 1H), 7.24 – 7.18 (m, 1H), 5.87 (ddt, J = 17.2, 10.4, 4.4 Hz, 1H), 5.29 (dd, J = 17.2, 0.9 Hz, 1H), 5.23 (dd, J = 10.5, 0.9 Hz, 1H), 4.14 (dt, J = 4.0, 1.8 Hz, 2H), 3.78 (d, J = 11.8 Hz, 2H), 2.77 (td, J = 12.3, 2.3 Hz, 2H), 2.62 – 2.52 (m, 2H), 2.41 (s, 3H), 2.24 – 2.18 (m, 2H), 2.13 (s, 3H) ppm. ^{13}C NMR (100 MHz, $CDCl_3$) δ 171.5, 143.4, 134.7, 132.5, 131.1, 129.5, 128.3, 128.1, 127.9, 122.2, 116.5, 88.8, 87.9, 57.86, 49.1, 44.1, 33.9, 24.6, 21.5 ppm. MS (ESI) *m*/*z* (%): 437.17 [(M + H)$^+$, 100]. Anal. Calcd. for $C_{25}H_{28}N_2O_3S$: C, 68.78; H, 6.46; N, 6.42. Found: C, 69.00; H, 6.53; N, 6.37.

(*rac*)-1-((3a'*R*,5'*S*)-5'-Hydroxy-6'-phenyl-3',3a',4',5'-tetrahydro-2'*H*-spiro[cyclohexane-1,1'-cyclopenta[*c*]pyrrol]-2'-yl)ethanone (36). To a solution of **5** (50 mg, 0.162 mmol) in a 1:1 mixture

of MeOH/CH_2Cl_2 (3.5 mL) were added successively $CeCl_3{\cdot}7H_2O$ (120 mg, 0.32 mmol) and $NaBH_4$ (12 mg, 0.32 mmol). The reaction mixture was left stirring at room temperature for 1 h, then the reaction was quenched with satd. NH_4Cl (4 mL) and 1M HCl (1 mL). The reaction mixture was partitioned between EtOAc and water, and the aqueous layer was extracted three times with EtOAc. The combined organic layer was washed with brine, dried over Na_2SO_4 and concentrated under reduced pressure. The crude product was purified by flash silica gel chromatography (2:1 EtOAc/petrol ether) to give **36** (46 mg, 92% yield) as a pale yellow oil. ^{1}H NMR (400 MHz, $CDCl_3$) δ 7.41 – 7.26 (m, 3H), 7.21 – 7.13 (m, 2H), 5.10 (t, J = 7.1 Hz, 1H), 3.79 (t, J = 8.8 Hz, 1H), 3.32 – 3.16 (m, 1H), 3.09 (t, J = 9.8 Hz, 1H), 2.95 (td, J = 13.5, 5.6 Hz, 1H), 2.65 (dt, J = 12.7, 7.1 Hz, 1H), 2.60 – 2.47 (m, 1H), 2.02 (s, 3H), 1.98 – 1.54 (m, 2H), 1.52 – 1.37 (m, 4H), 1.14 – 1.02 (m, 4H). ^{13}C NMR (100 MHz, $CDCl_3$) δ 168.8, 148.3, 137.3, 136.5, 129.5, 128.2, 127.4, 84.9, 66.6, 54.5, 43.9, 37.7, 32.6, 28.8, 25.4, 24.3, 23.4, 22.1. MS (ESI) *m/z* (%): 334.27 (100, $[M + Na]^+$). Anal. Calcd. for $C_{20}H_{25}NO_2$: C, 77.14; H, 3.09; N, 4.50. Found: C, 77.50; H, 3.21; N, 4.39.

(*rac*)- 1-((1a’S,2’S,3a’S,6a’S)-2’-Hydroxy-1a’-phenyltetrahydrospiro[cyclohexane-1,6’-oxireno[2’,3’:1,5]cyclopenta[1,2-*c*]pyrrol]-5’(1a’*H*)-yl)ethanone (37). To a solution of compound **36** (43 mg, 0.154 mmol) in anhydrous CH_2Cl_2 (1 mL) mCPBA (34 mg, 80%, 0.154 mmol) was added at 0 °C, and the reaction mixture was stirred at 0 °C for 3 h. The reaction was quenched with satd. $NaHCO_3$ (2 mL) and the resulting mixture was partitioned between EtOAc and water. The aqueous layer was extracted three times with EtOAc, washed with brine (20 mL), dried over Na_2SO_4 and concentrated under reduced pressure. The crude product was purified by flash silica gel chromatography (1:1 EtOAc/petrol ether) to give pure **37** (34 mg, 68% yield) as a colorless oil. ^{1}H NMR (400 MHz, $CDCl_3$) δ 7.44 – 7.32 (m, 5H), 4.59 (t, J = 7.7 Hz, 1H), 3.69 (t, J = 8.5 Hz, 1H), 3.36 – 3.25 (m, 1H), 2.79 – 2.69 (m, 1H), 2.67 – 2.54 (m, 1H), 2.07 – 2.01 (m, 1H), 2.04 (s, 3H), 1.76 – 1.42 (m, 4H), 1.35 – 1.03 (m, 7H). ^{13}C NMR (100 MHz, $CDCl_3$) δ 169.1, 133.5, 128.5 (2C), 128.4, 127.8 (2C), 83.7, 80.0, 73.7, 66.4, 49.9, 38.5, 30.9, 28.1, 26.0, 25.3, 23.9, 23.0,

21.9. MS (ESI) *m/z* (%): 350.34 (100, $[M + Na]^+$). Anal. Calcd. for $C_{20}H_{25}NO_3$: C, 73.37; H, 7.70; N, 4.28. Found: C, 73.78; H, 7.79; N, 4.20.

(*rac*)- 1-((3a'*R*,5'*S*)-5'-Ethyl-5'-hydroxy-6'-phenyl-3',3a',4',5'-tetrahydro-2'*H*-spiro[cyclohexane-1,1'-cyclopenta[*c*]pyrrol]-2'-yl)ethanone (38). $CeCl_3$ (40 mg, 0.162 mmol) was dried under vacuum and transferred under a nitrogen atmosphere to a round bottom flask. Then, a solution of compound **5** (50 mg, 0.162 mmol) in THF (1 mL) was added and stirred at room temperature for 1 h. The mixture was cooled to 0 °C before adding dropwise the Grignard reagent as a 3 M solution in Et_2O (300 µL, 0.810 mmol). The resulting mixture was left stirring at 0 ° C for 30 min, then quenched by carefully adding a saturated solution of NH_4Cl (1 mL). The solution was left under stirring for 30 min before pouring a saturated solution of NH_4Cl (15 mL). Then, the resulting mixture was extracted with Et_2O, washed with water and brine, dried over Na_2SO_4 and concentrated under reduced pressure. The crude product was purified by flash silica gel chromatography (1:1 EtOAc/petrol ether) to give **38** (26 mg, 48% yield) as a yellow oil. ^{1}H NMR (400 MHz, $CDCl_3$) δ 7.36 – 7.33 (m, 3H), 7.19 – 7.16 (m, 2H), 3.78 (t, *J* = 8.7 Hz, 1H), 3.24 – 3.12 (m, 1H), 3.06 (t, *J* = 9.6 Hz, 1H), 2.89 (td, *J* = 13.2, 5.6 Hz, 1H), 2.57 (dt, *J* = 12.7, 5.1 Hz, 1H), 2.45 (dd, *J* = 13.0, 7.3 Hz, 1H), 2.03 (s, 3H), 1.75 – 1.61 (m, 4H), 1.58 – 1.40 (m, 4H), 1.36 – 1.25 (m, 4H), 1.00 (t, *J* = 7.4 Hz, 3H). ^{13}C NMR (100 MHz, $CDCl_3$) δ 168.6, 148.9, 137.5, 135.5, 127.9, 127.5, 92.4, 66.6, 54.8, 43.8, 40.5, 32.6, 31.9, 28.6, 25.4, 24.2, 23.4, 21.7, 8.53. MS (ESI) *m/z* (%): 362.28 (100, $[M + Na]^+$). Anal. Calcd. for $C_{22}H_{29}NO_2$: C, 77.84; H, 8.61; N, 4.13. Found: C, 78.12; H, 8.70; N, 4.05.

(*rac*)-2'-Acetyl-7'-phenyl-3',3a',4',5'-tetrahydrospiro[cyclohexane-1,1'-pyrrolo[3,4-*c*]pyridin]-6'(2'*H*)-one (39). To a stirred solution of compound **5** (50 mg, 0.162 mmol) in TFA (600 µL) was added NaN_3 (19 mg, 0.29 mmol) and the reaction mixture was heated at reflux temperature for 16 h. After completing the reaction, the mixture was cooled to room temperature and evaporated under reduced pressure. The resulting crude was treated with water and extracted with CH_2Cl_2. The

combined organic phase was successively washed with satd. $NaHCO_3$, water, and brine, then dried over Na_2SO_4 and concentrated under reduced pressure. The crude product was purified by flash silica gel chromatography (EtOAc) to give pure **39** (22 mg, 0.067 mmol, 41%) as a colorless oil. ^{1}H NMR (400 MHz, $CDCl_3$) δ 7.35 – 7.25 (m, 5H), 5.56 (br s, 1H), 3.83 – 3.67 (m, 1H), 3.40 (d, J = 4.4 Hz, 1H), 3.17 – 3.00 (m, 1H), 2.70 (dd, J = 18.7, 6.9 Hz, 1H), 2.34 (d, J = 18.7 Hz, 1H), 2.12 – 2.02 (m, 2H), 1.98 (s, 3H), 1.83 – 1.56 (m, 8H).^{13}C NMR (100 MHz, $CDCl_3$) δ 172.2, 165.2, 133.6, 132.5, 132.0, 129.0, 128.0, 127.8, 59.6, 42.7, 40.2, 39.4, 27.5, 25.7, 23.3, 22.4, 21.6. MS (ESI) *m*/*z* (%): 347.25 (100, $[M + Na]^+$). Anal. Calcd. for $C_{20}H_{24}N_2O_2$: C, 74.04; H, 7.46; N, 3.64. Found: C, 74.56; H, 7.53; N, 3.57.

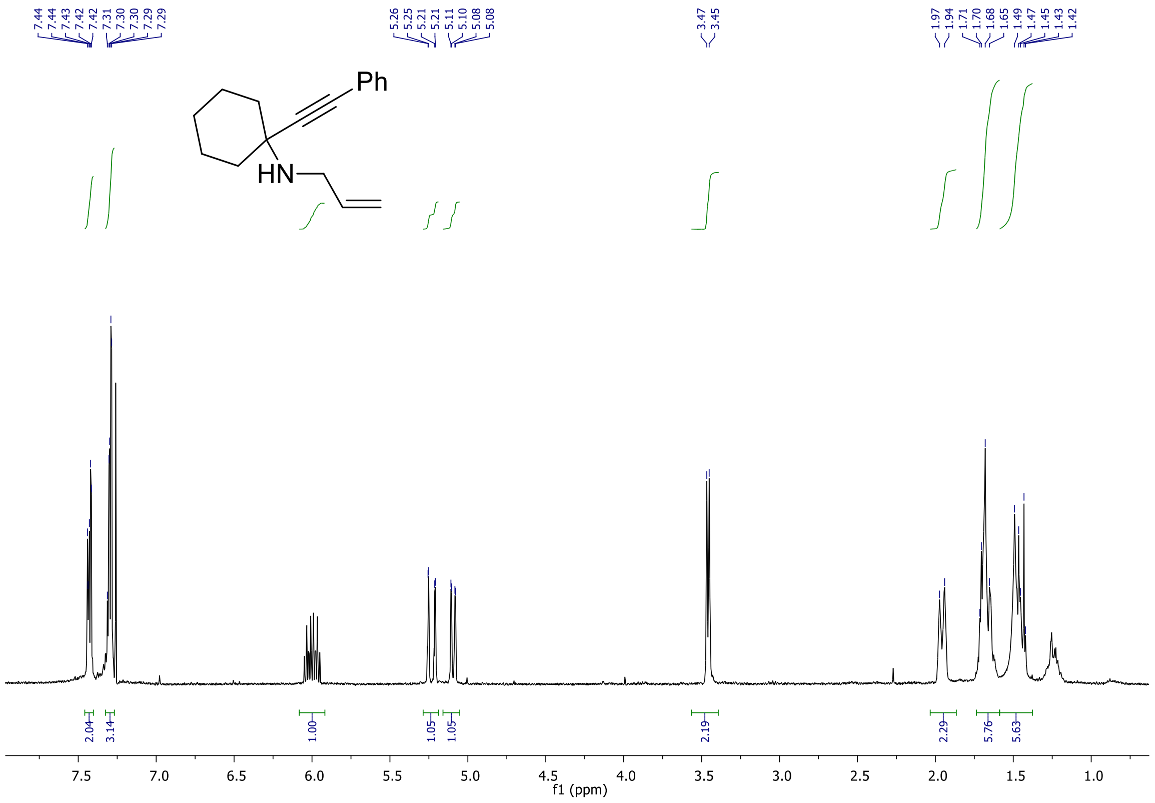

Figure S1. ^{1}H NMR spectrum of compound **3** (400 MHz, $CDCl_3$).

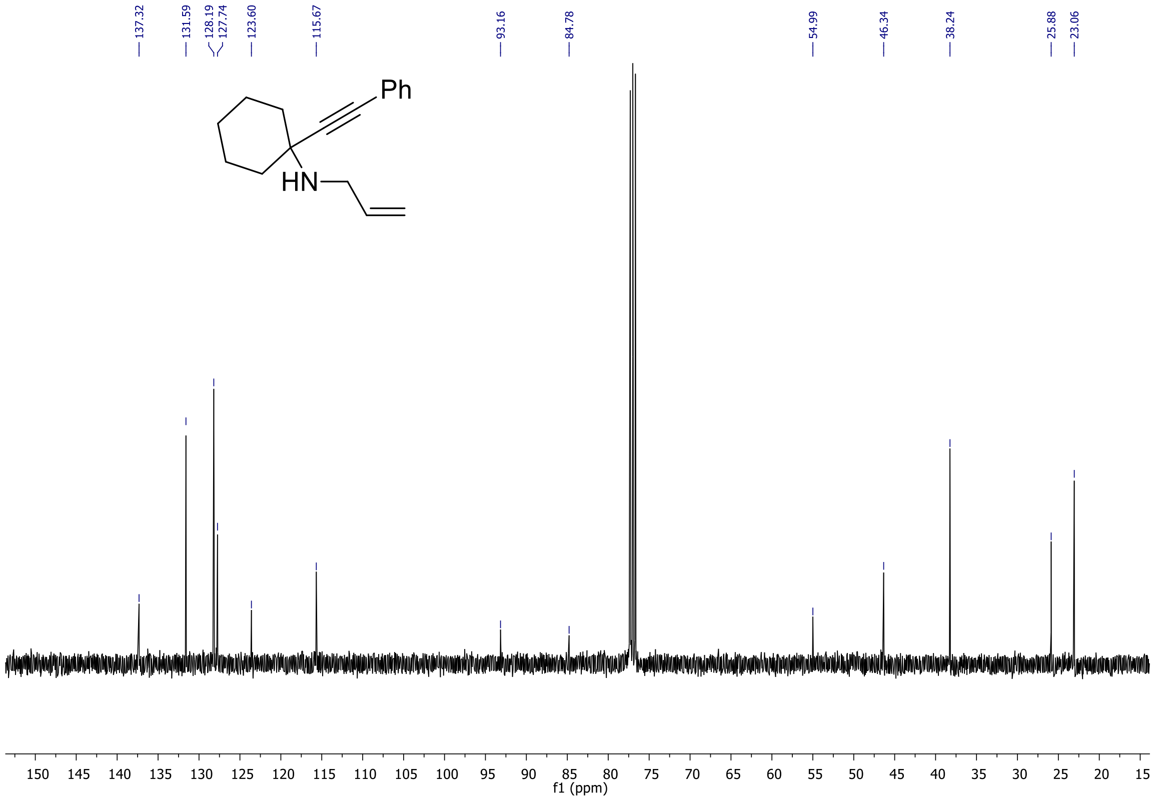

Figure S2. ^{13}C NMR spectrum of compound **3** (100 MHz, $CDCl_3$).

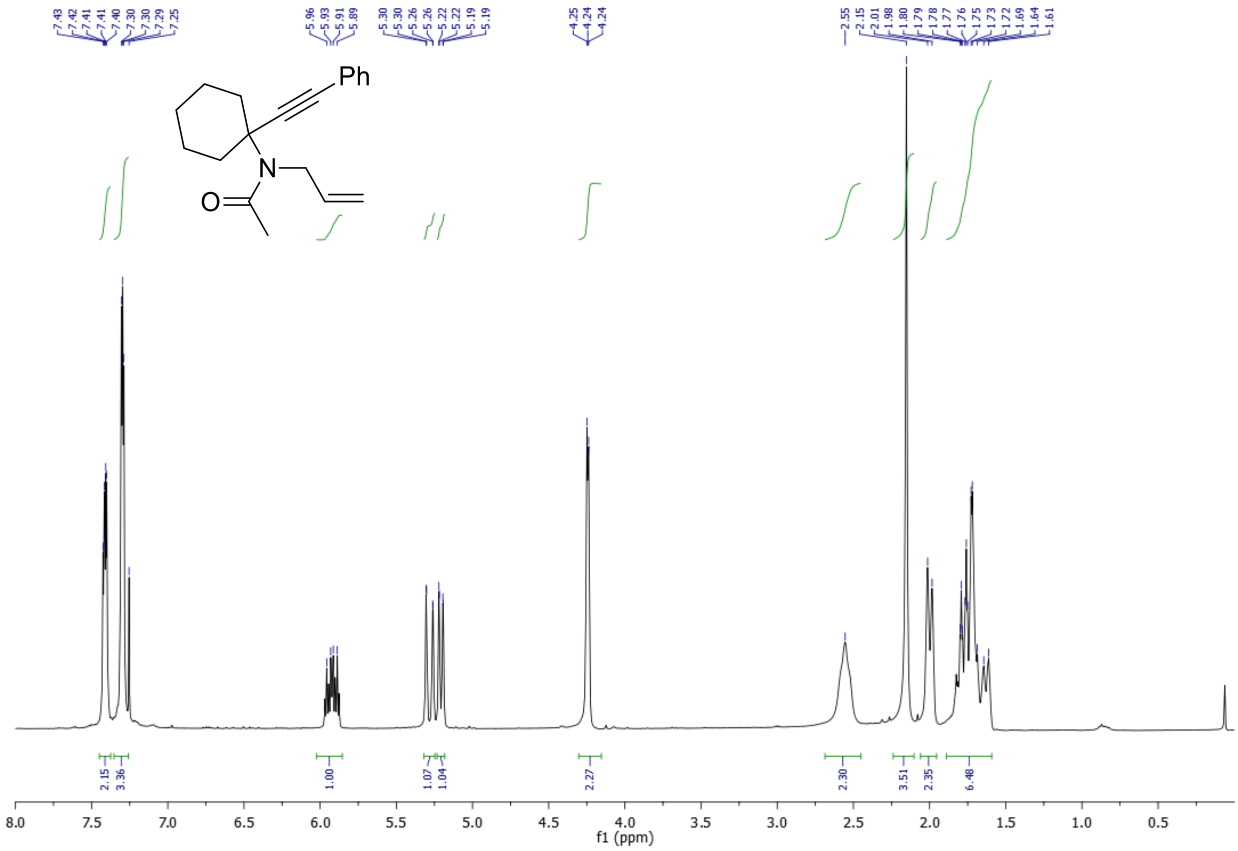

Figure S3. ^{1}H NMR spectrum of compound **4** (400 MHz, $CDCl_3$).

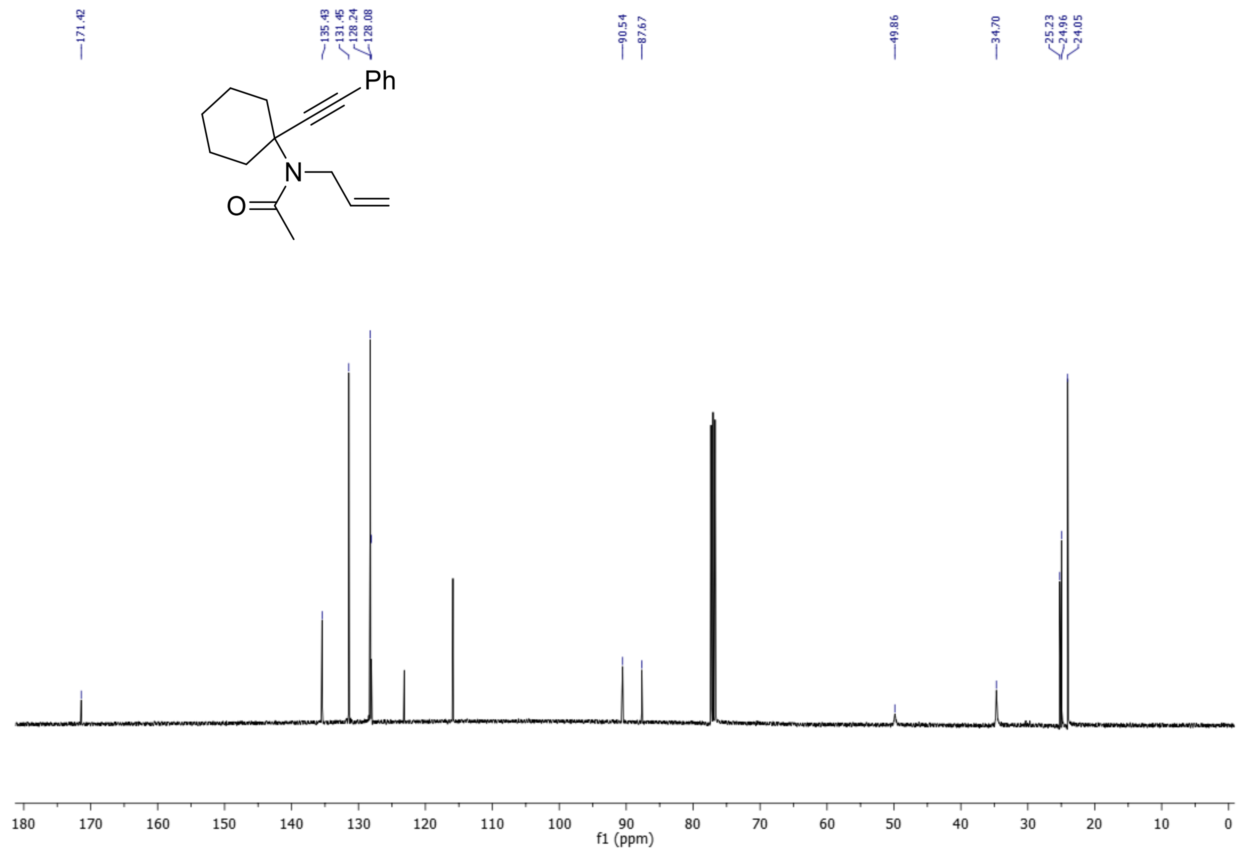

Figure S4. ^{13}C NMR spectrum of compound **4** (100 MHz, $CDCl_3$).

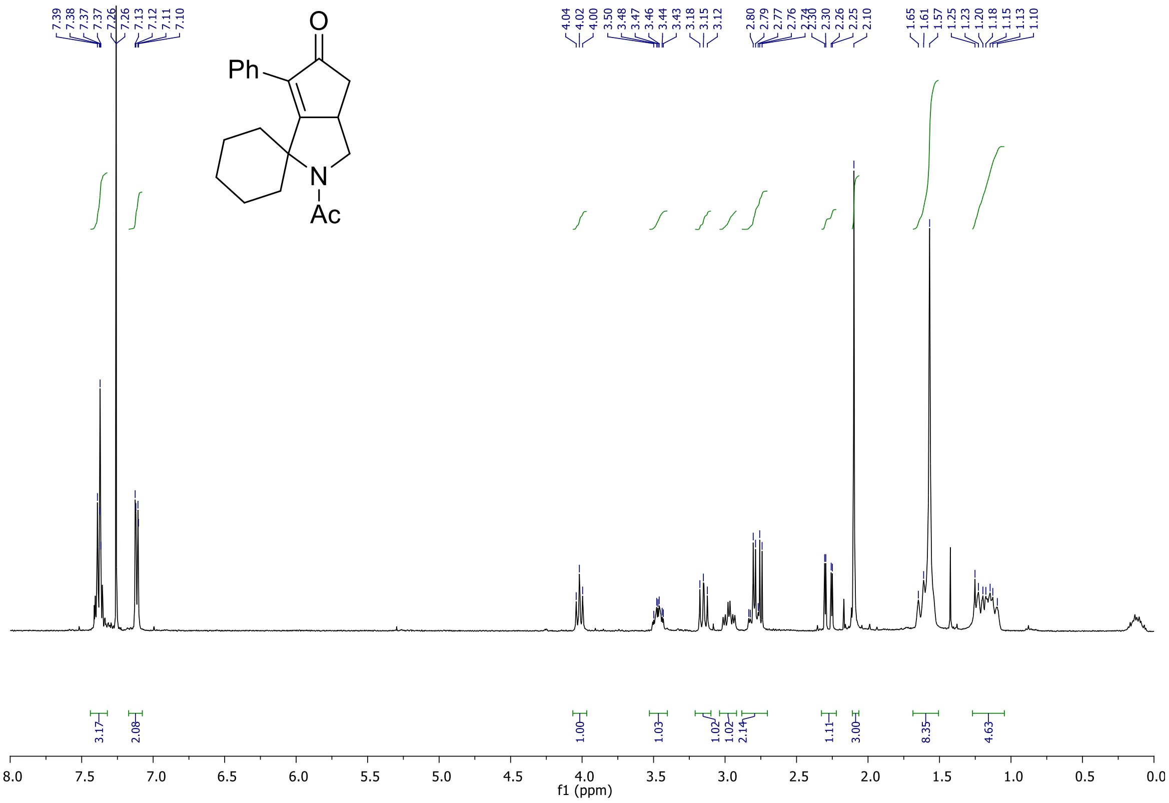

Figure S5. ^{1}H NMR spectrum of compound **5** (400 MHz, $CDCl_3$).

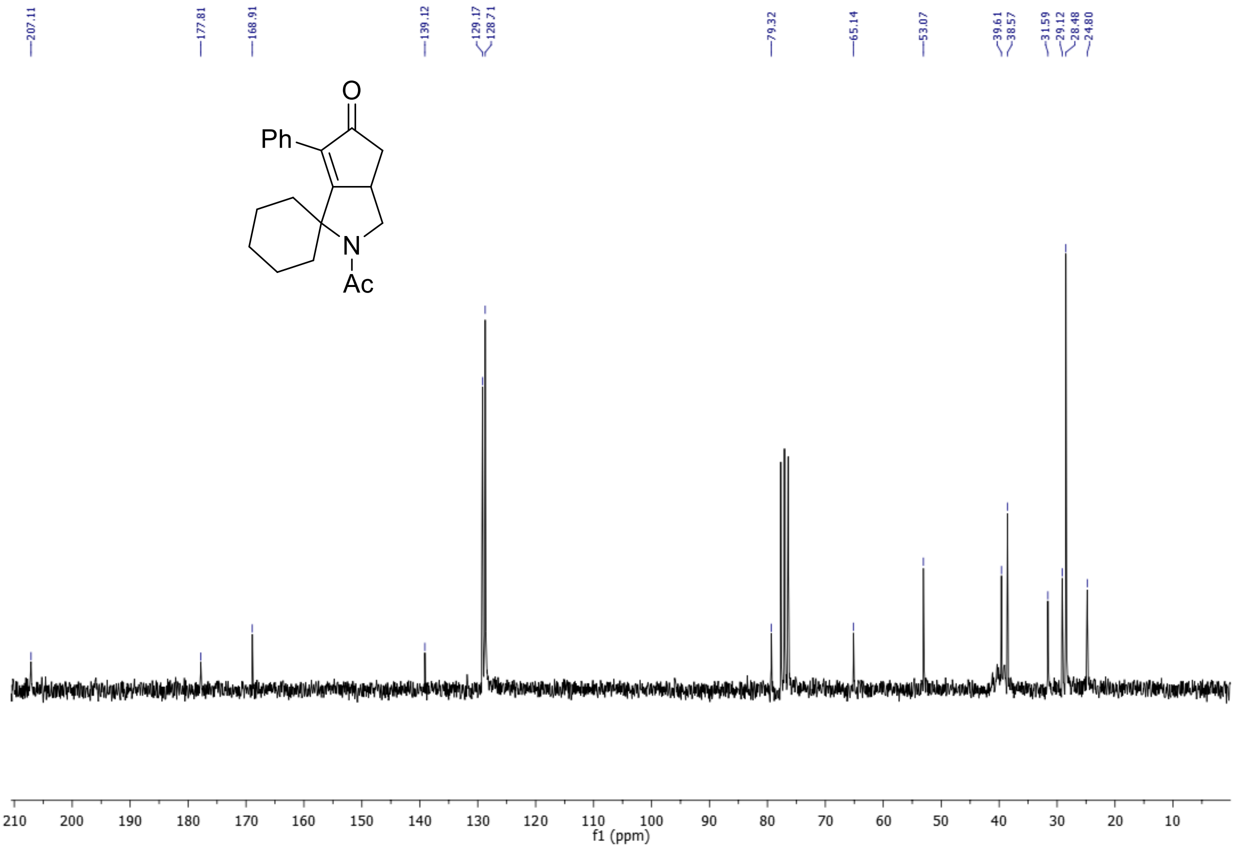

Figure S6. ^{13}C NMR spectrum of compound **5** (50 MHz, $CDCl_3$).

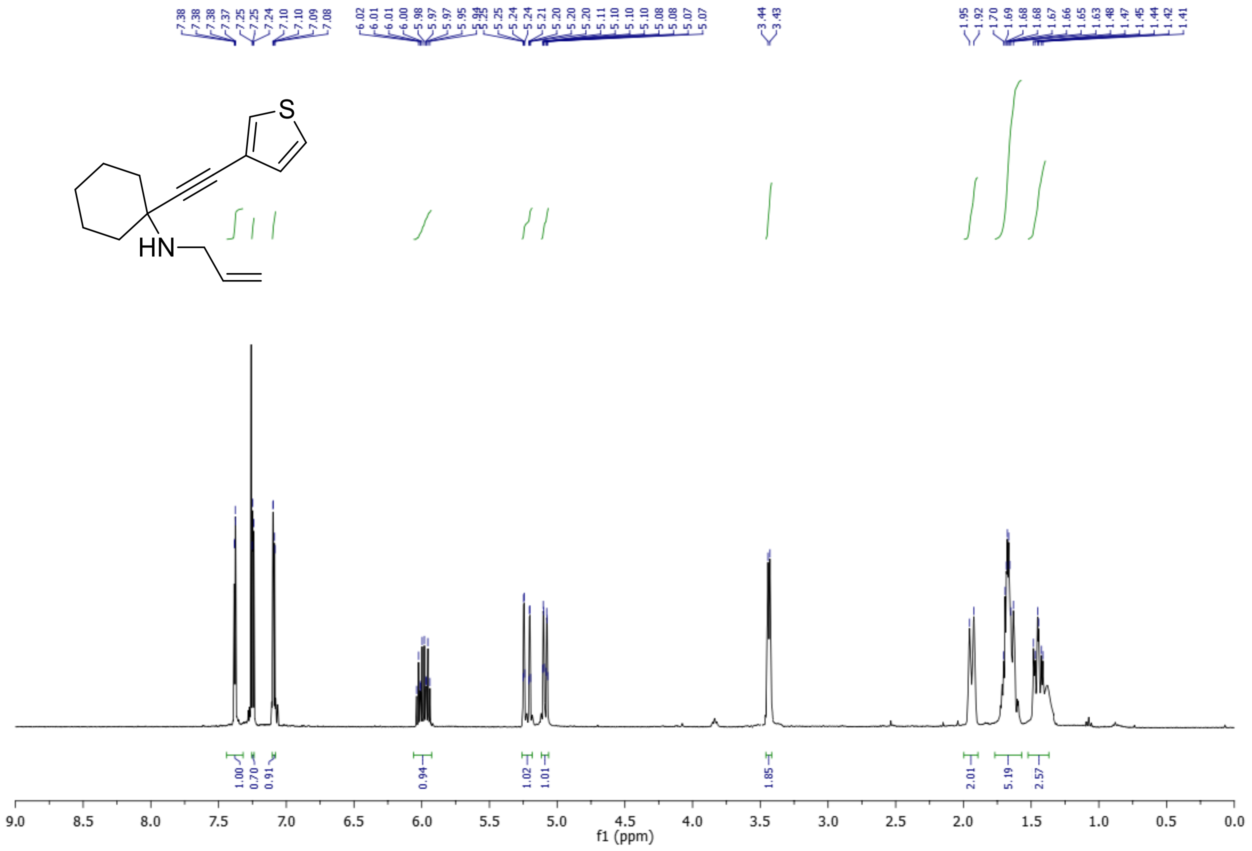

Figure S7. ^{1}H NMR spectrum of compound **7** (400 MHz, $CDCl_3$).

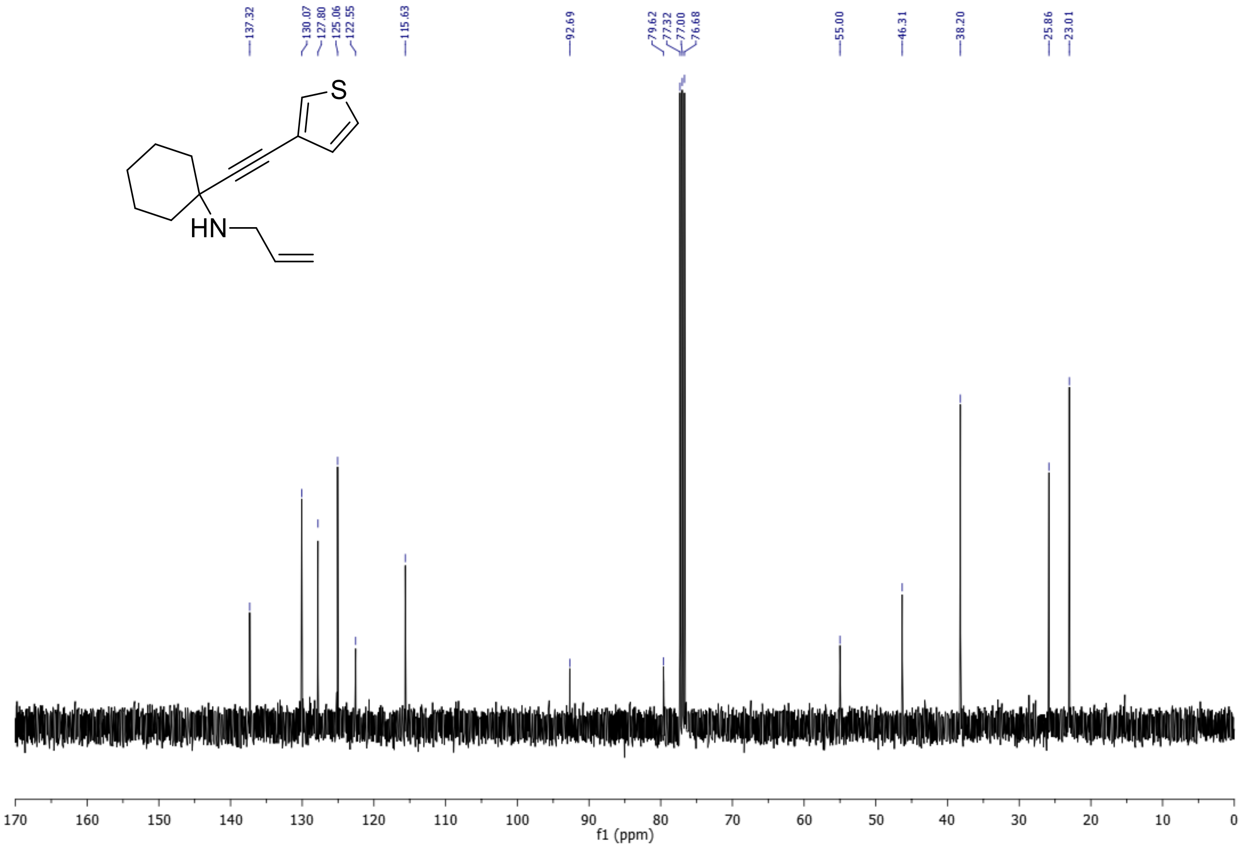

Figure S8. ^{13}C NMR spectrum of compound **7** (100 MHz, $CDCl_3$).

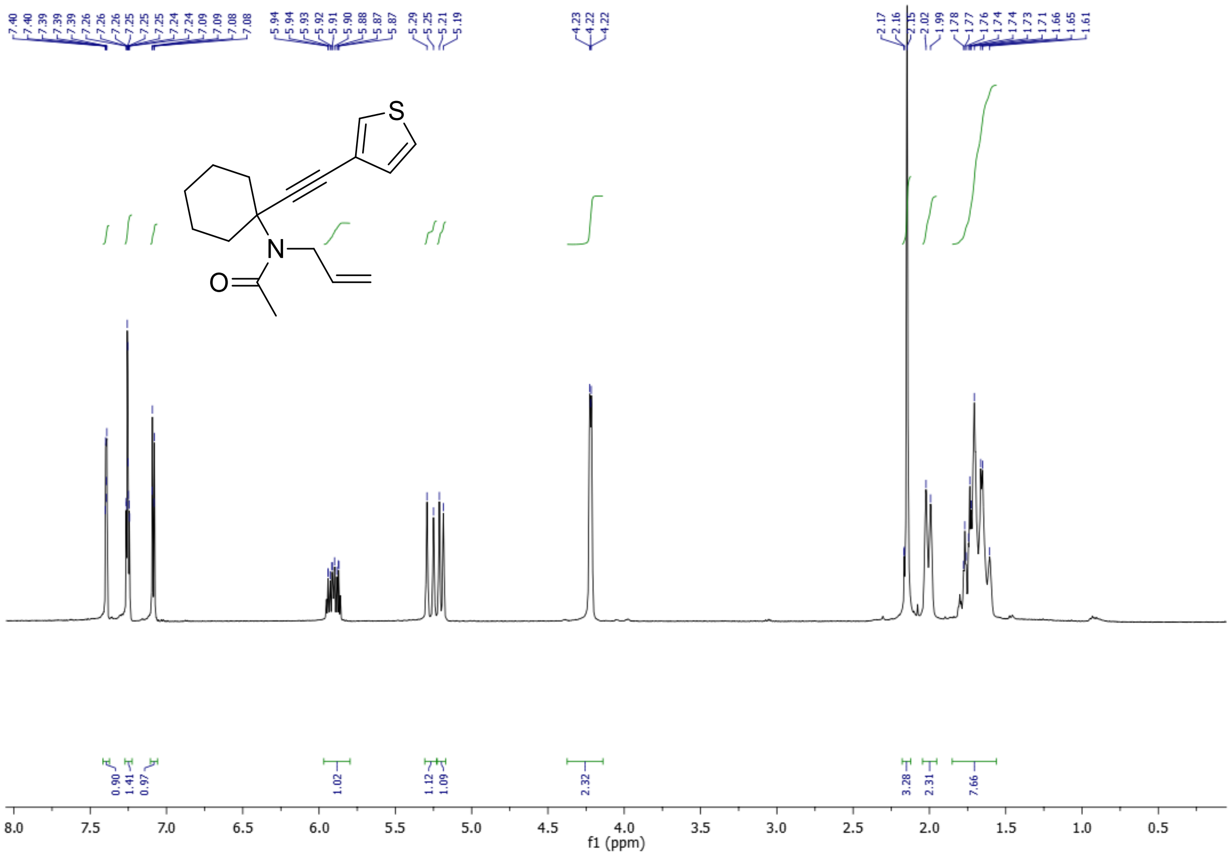

Figure S9. ^{1}H NMR spectrum of compound **8** (400 MHz, $CDCl_3$).

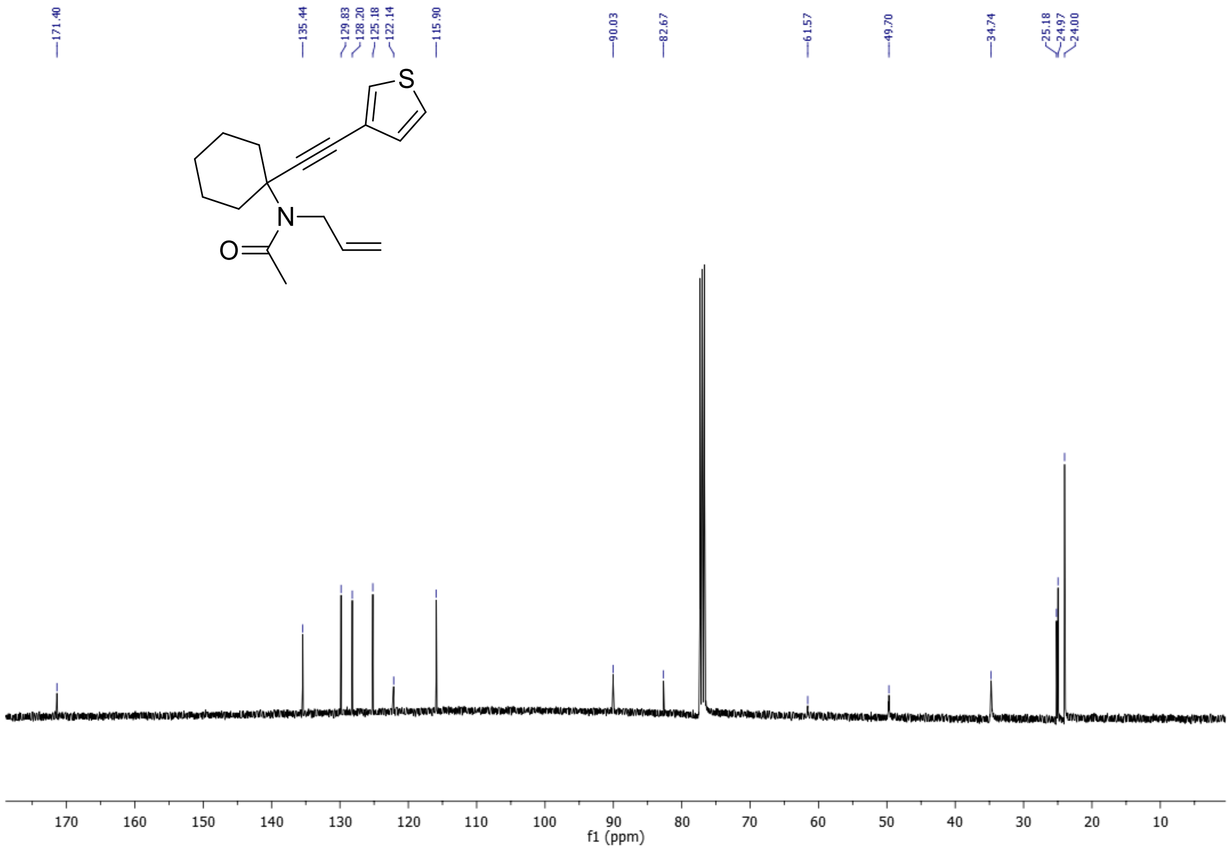

Figure S10. ^{13}C NMR spectrum of compound **8** (100 MHz, $CDCl_3$).

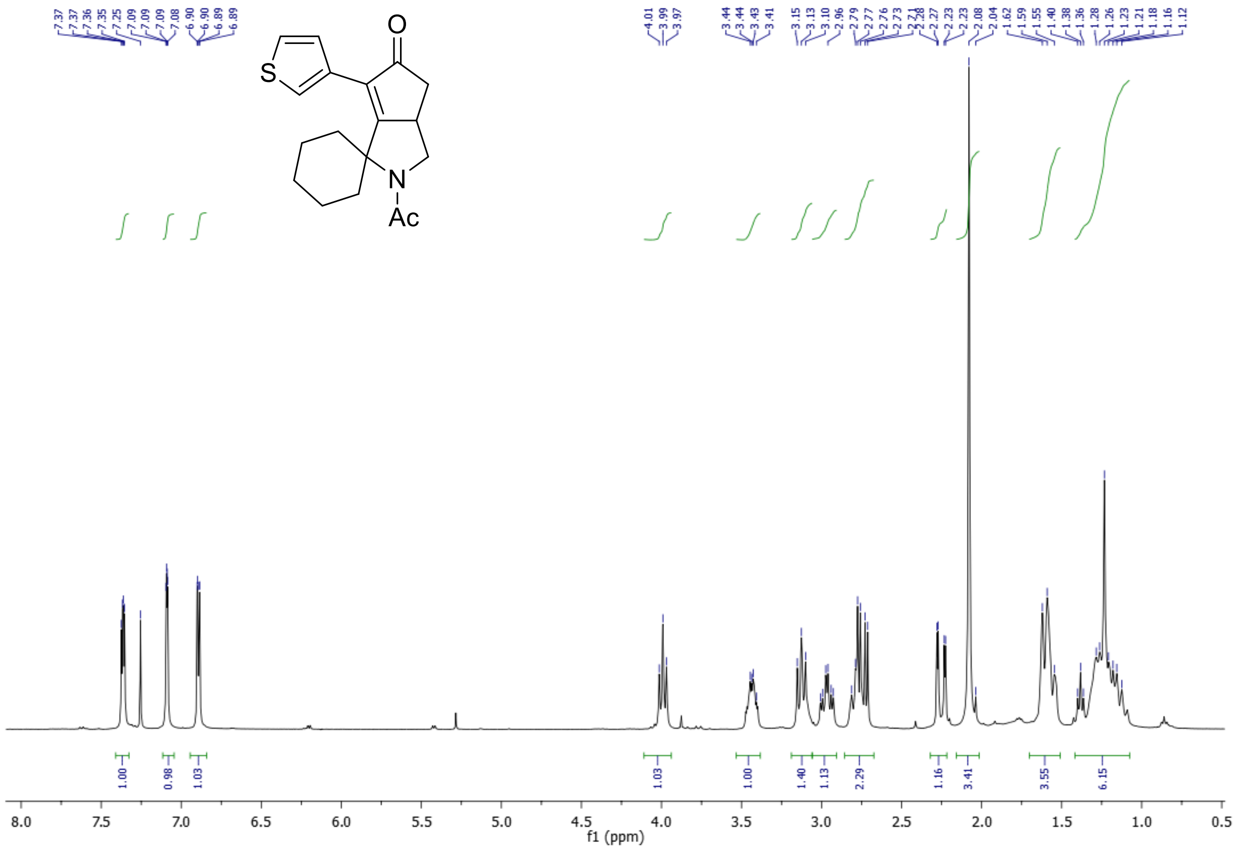

Figure S11. ^{1}H NMR spectrum of compound **9** (400 MHz, $CDCl_3$).

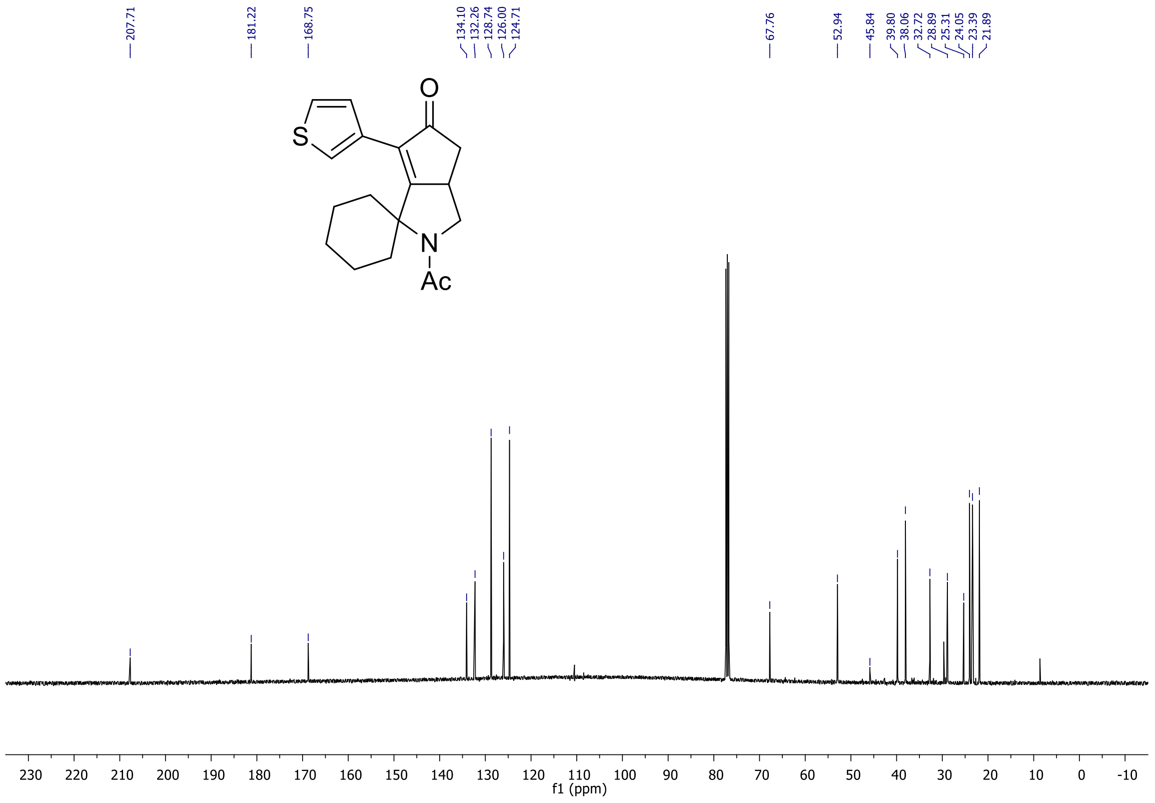

Figure S12. ^{13}C NMR spectrum of compound **9** (100 MHz, $CDCl_3$).

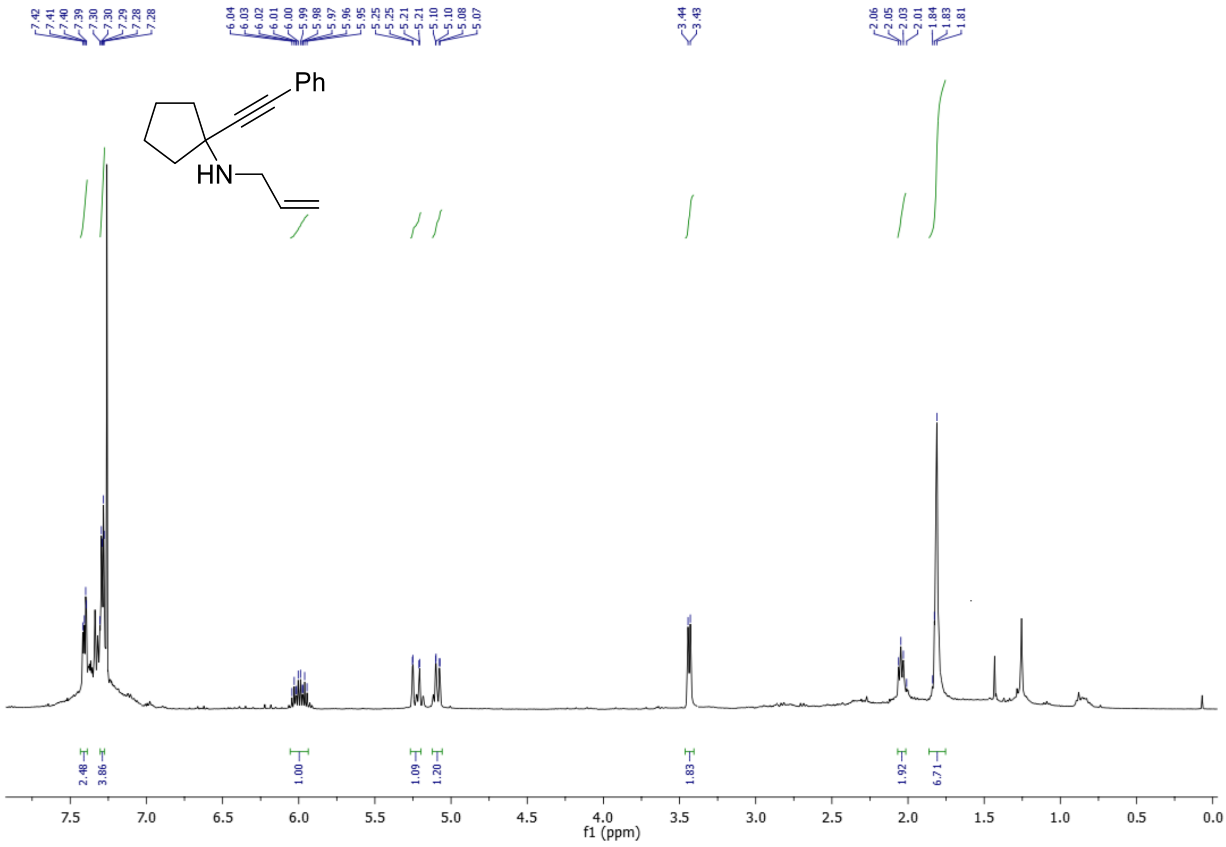

Figure S13. ^{1}H NMR spectrum of compound **13** (400 MHz, $CDCl_3$).

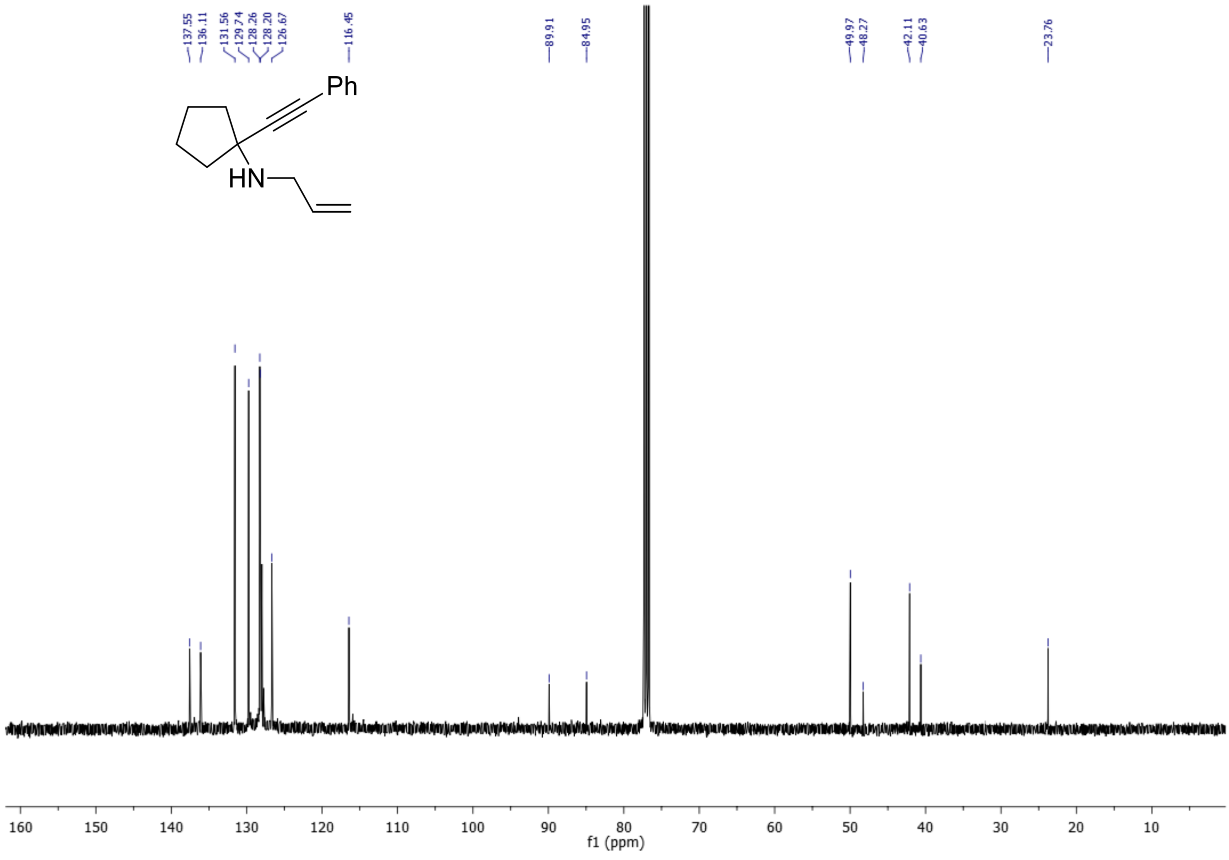

Figure S14. ^{13}C NMR spectrum of compound **13** (100 MHz, $CDCl_3$).

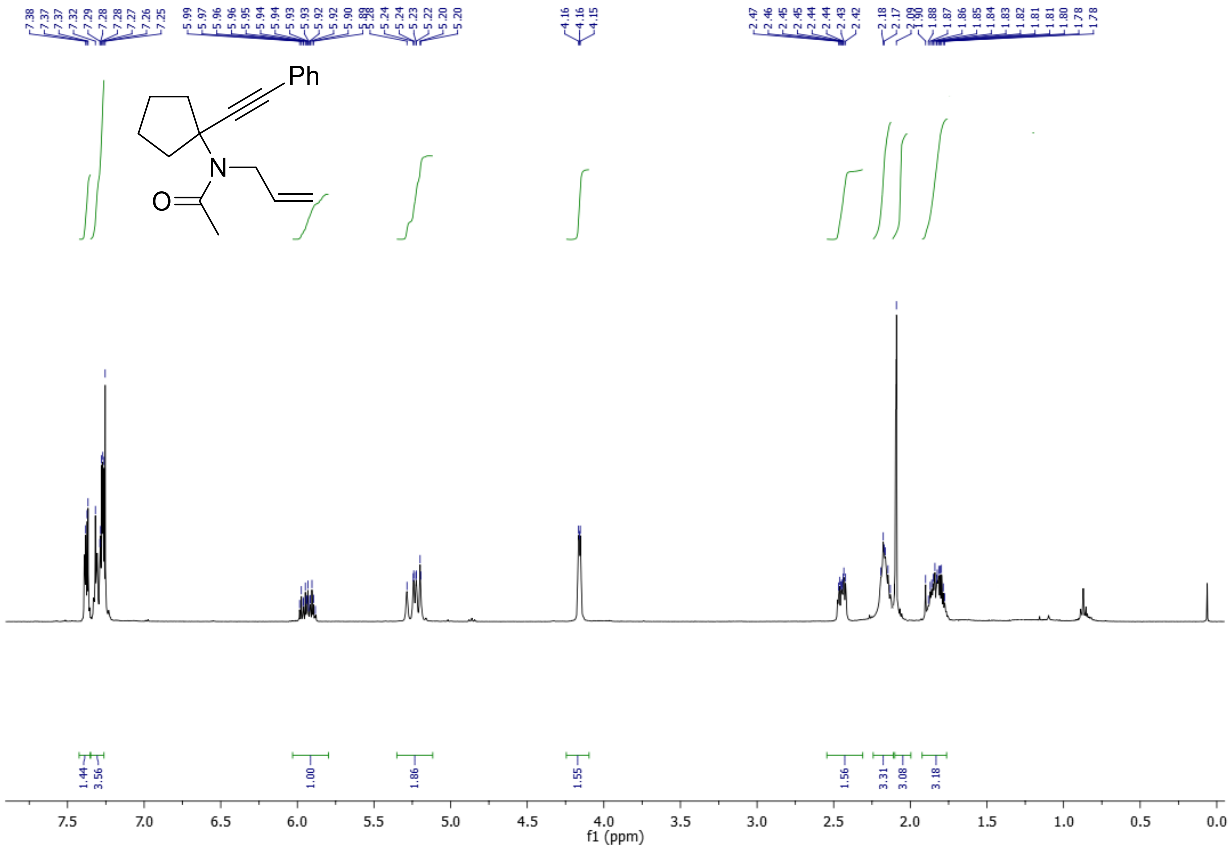

Figure S15. ^{1}H NMR spectrum of compound **14** (400 MHz, $CDCl_3$).

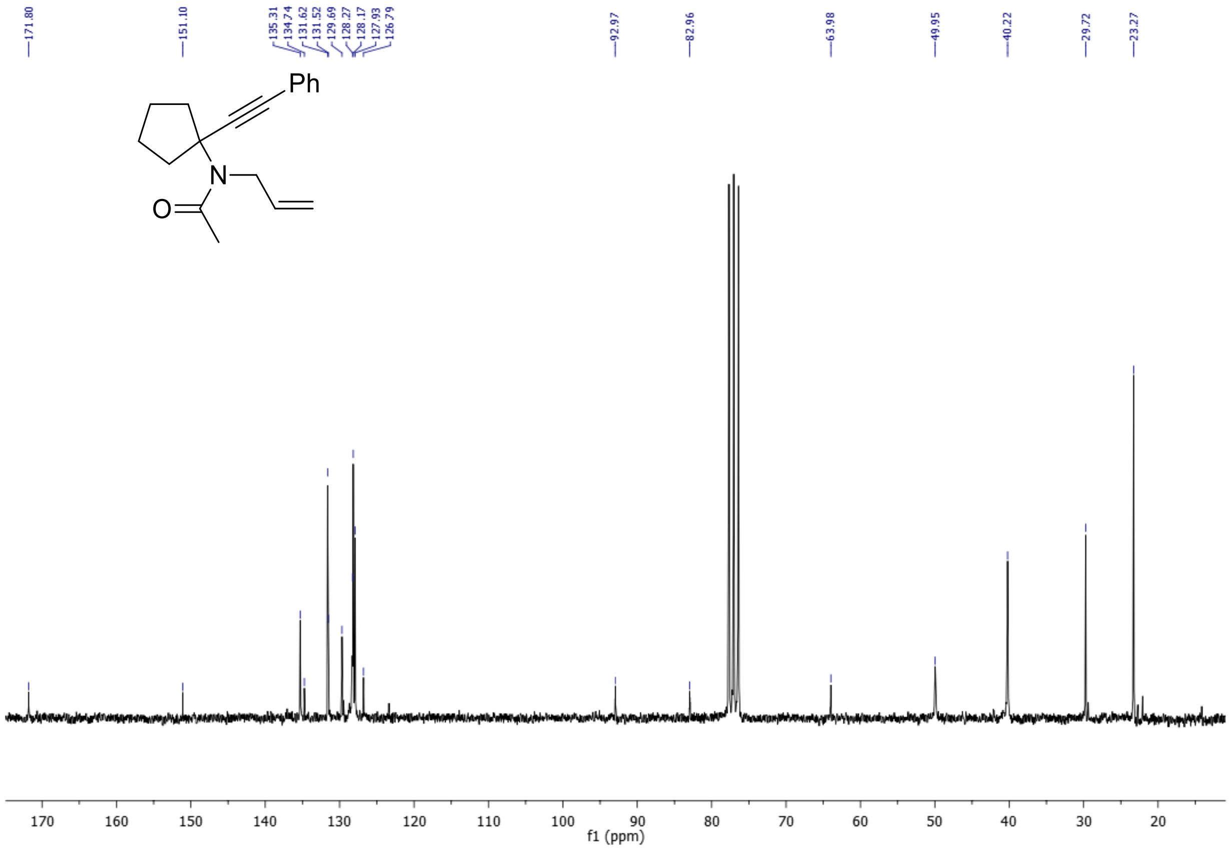

Figure S16. ^{13}C NMR spectrum of compound **14** (50 MHz, $CDCl_3$).

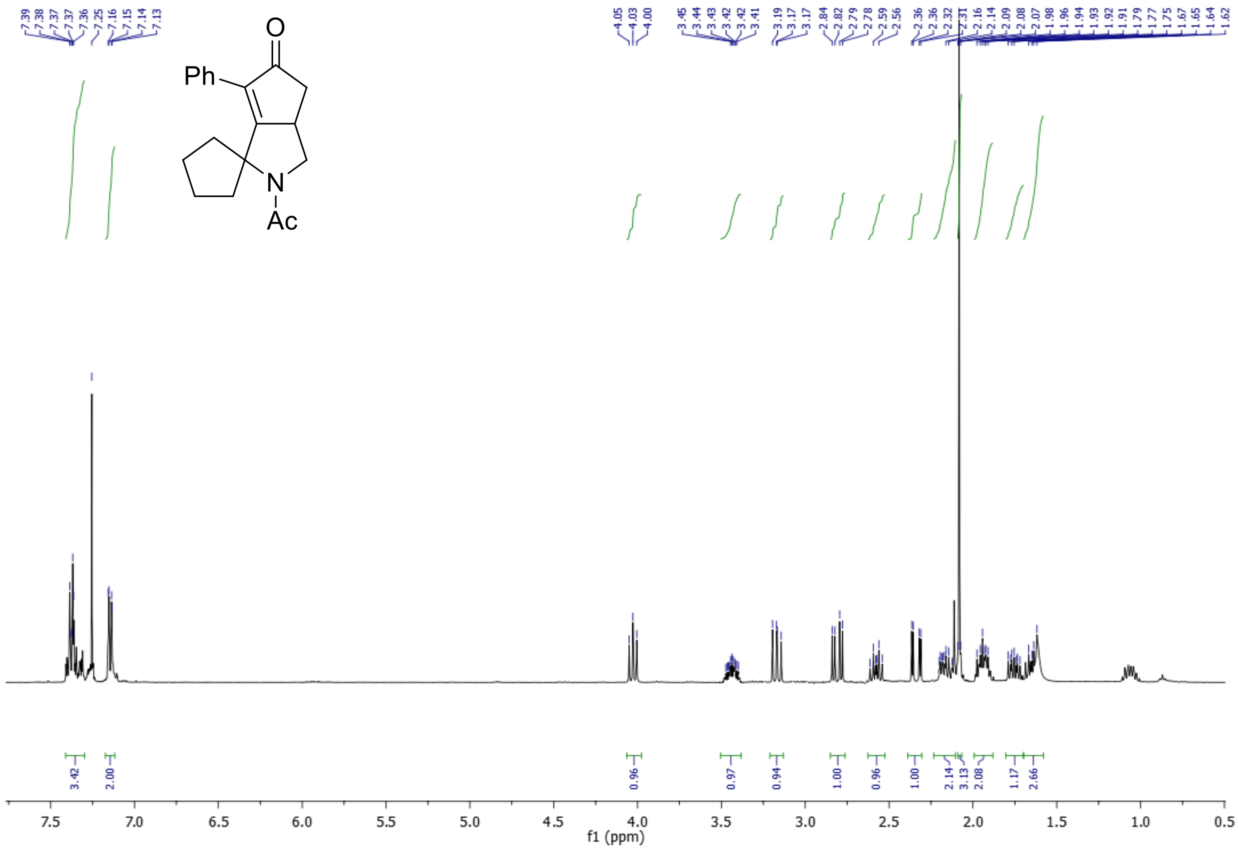

Figure S17. ^{1}H NMR spectrum of compound **15** (400 MHz, $CDCl_3$).

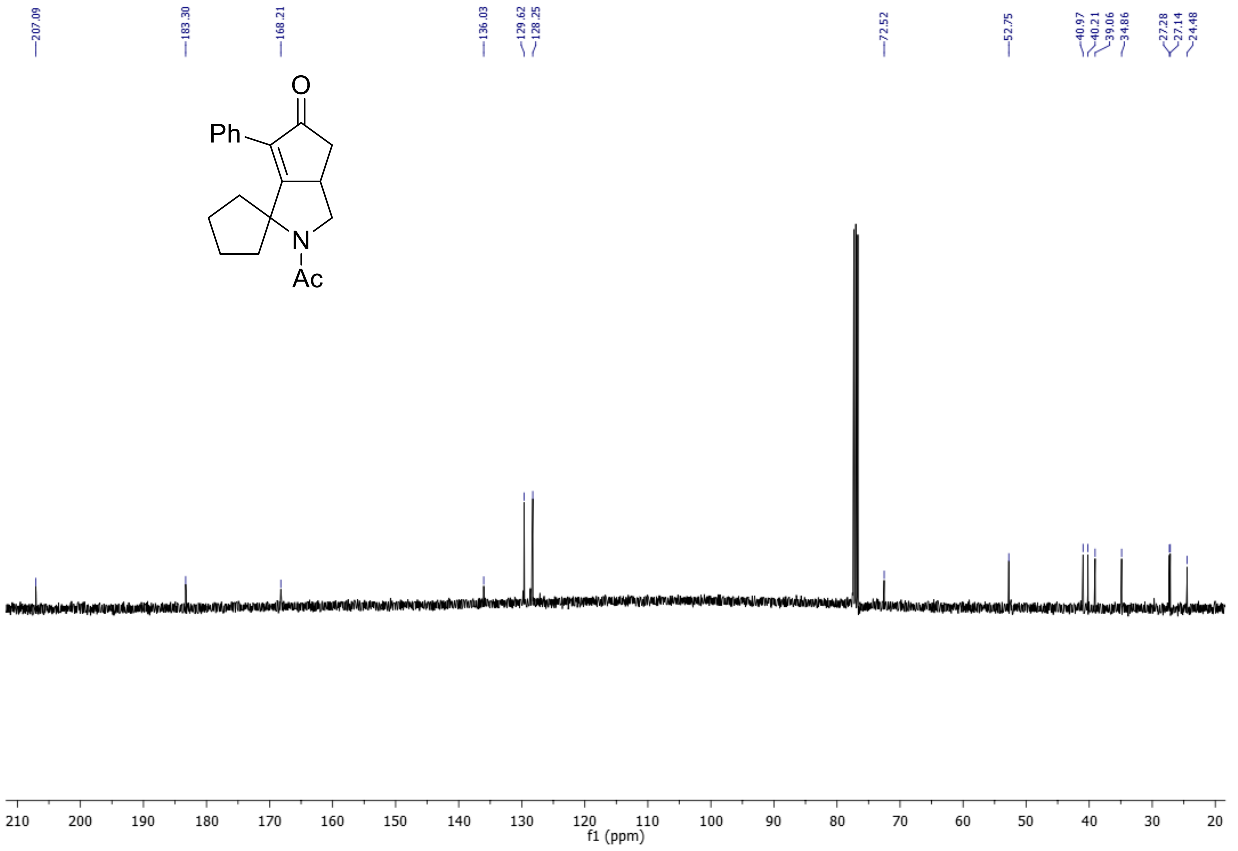

Figure S18. ^{13}C NMR spectrum of compound **15** (100 MHz, $CDCl_3$).

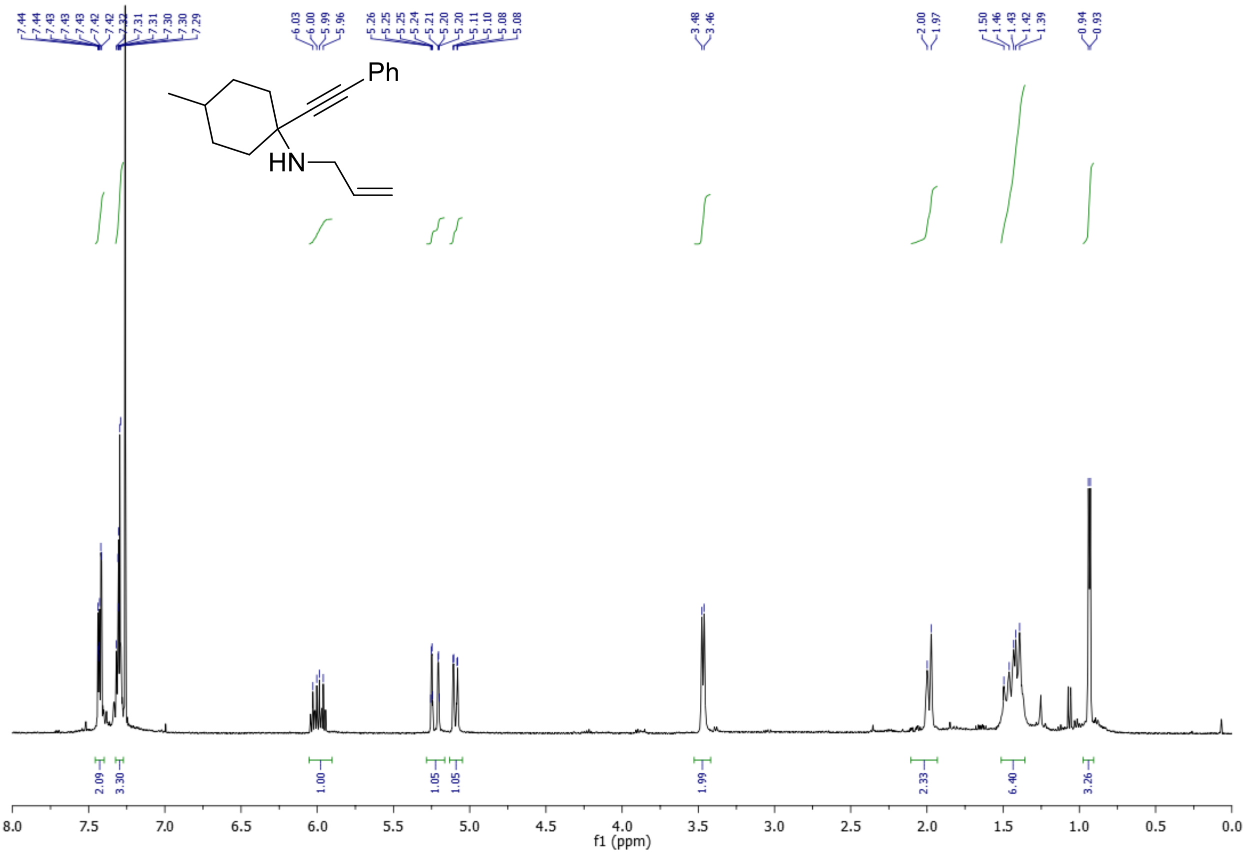

Figure S19. ^{1}H NMR spectrum of compound **17** (400 MHz, $CDCl_3$).

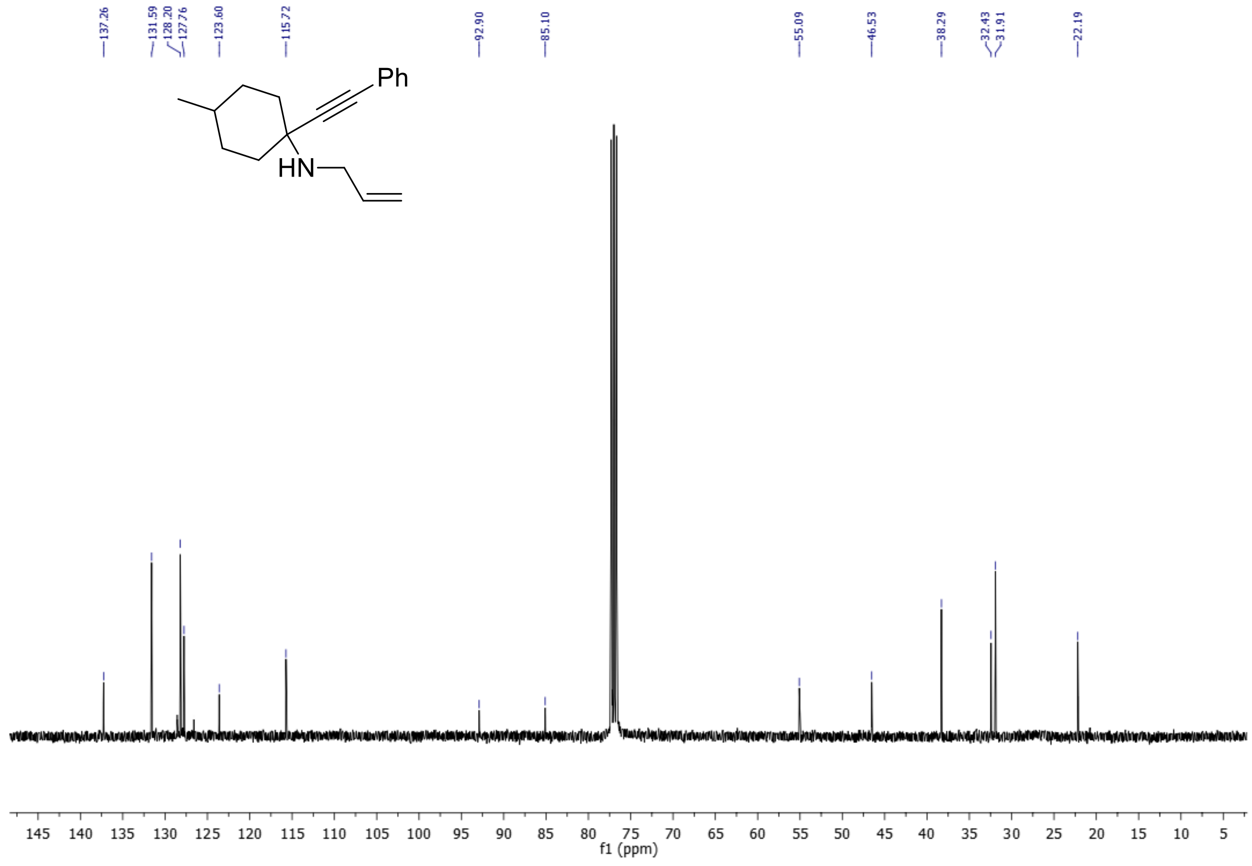

Figure S20. ^{13}C NMR spectrum of compound **17** (100 MHz, $CDCl_3$).

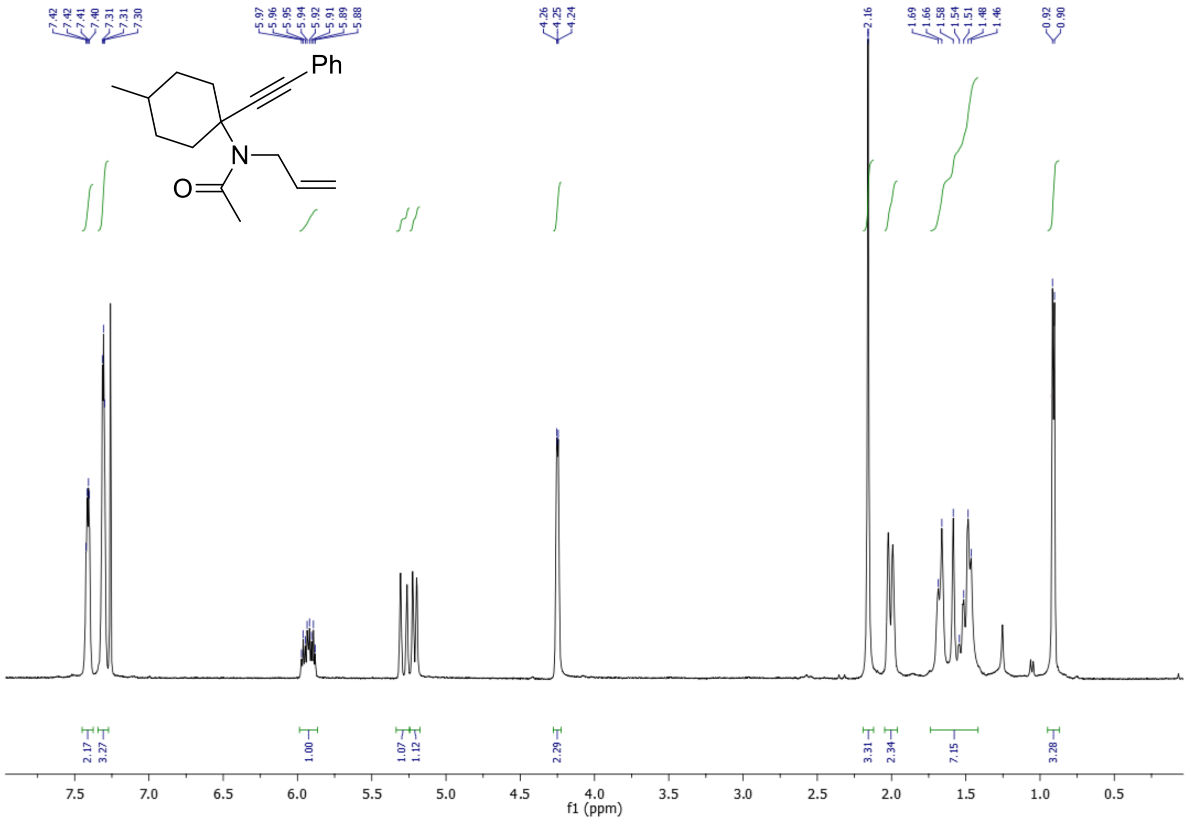

Figure S21. ^{1}H NMR spectrum of compound **18** (400 MHz, $CDCl_3$).

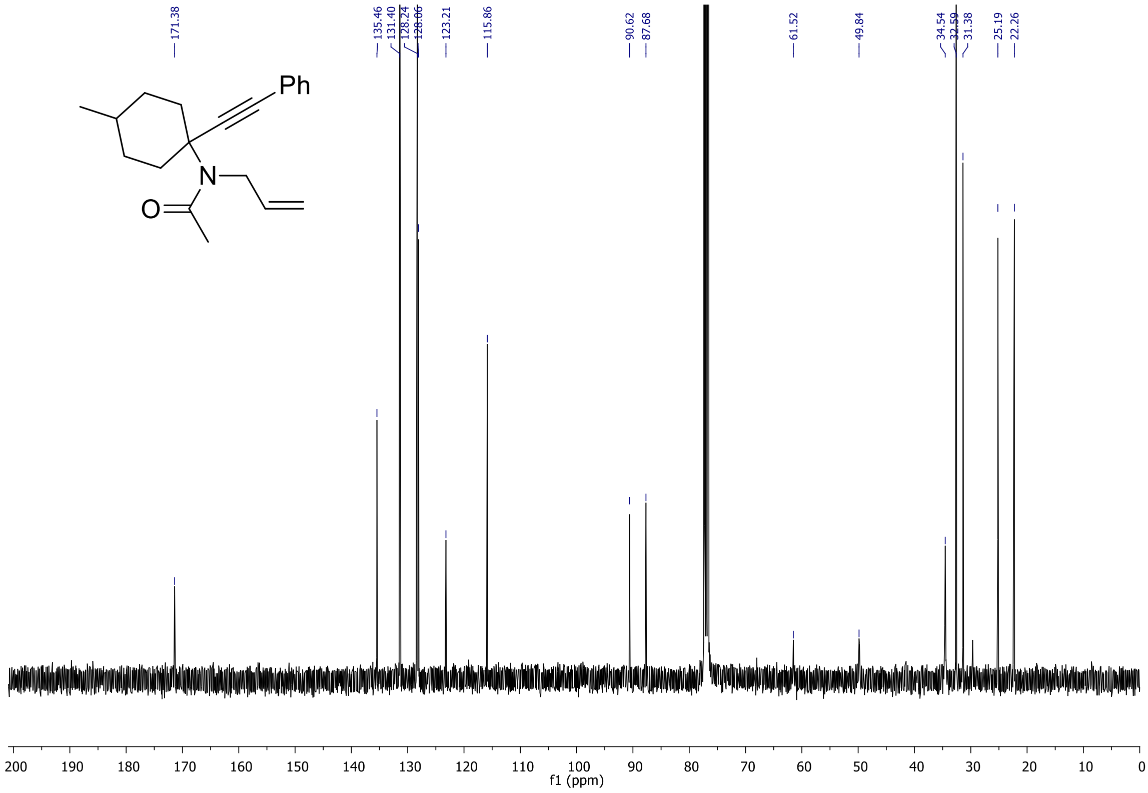

Figure S22. ^{13}C NMR spectrum of compound **18** (100 MHz, $CDCl_3$).

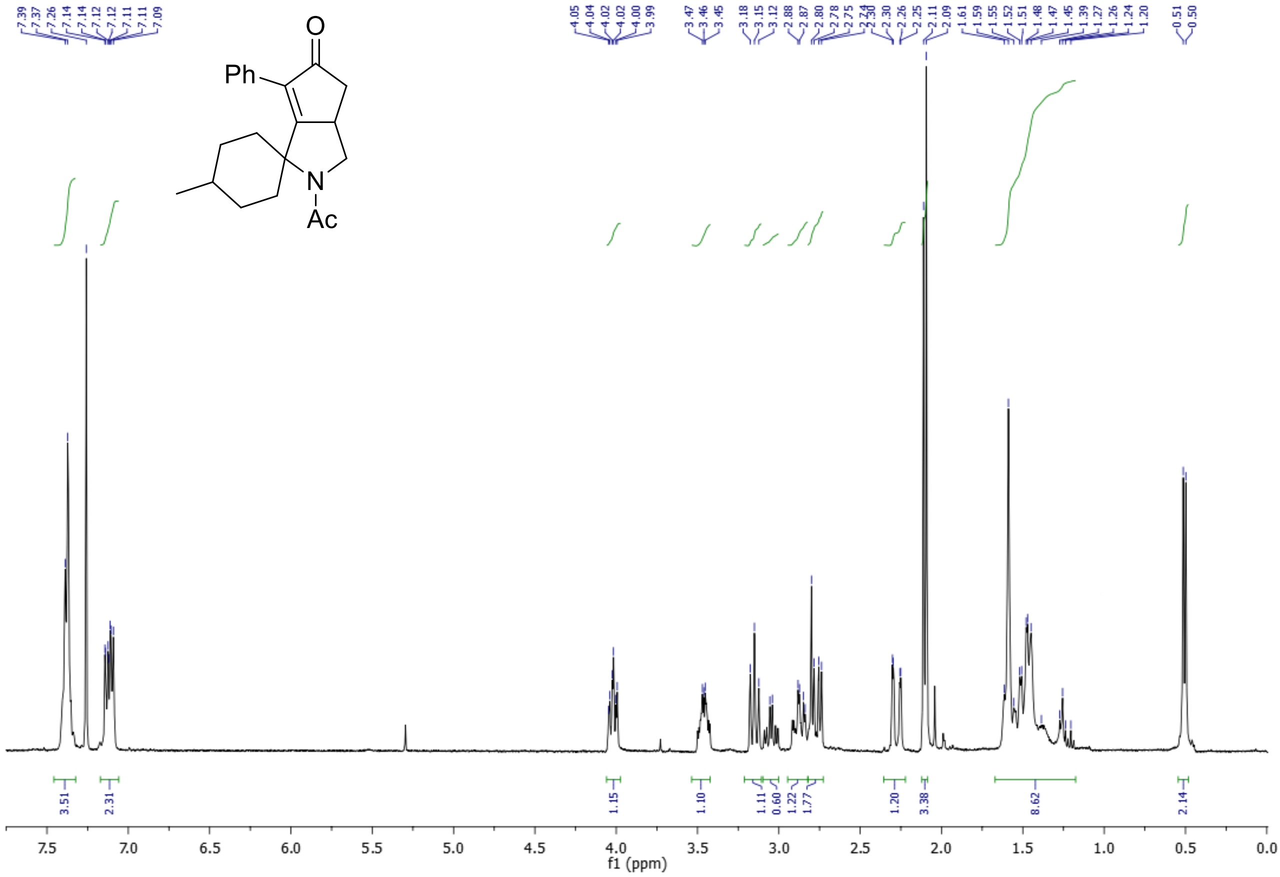

Figure S23. ^{1}H NMR spectrum of compound **19** (400 MHz, $CDCl_3$).

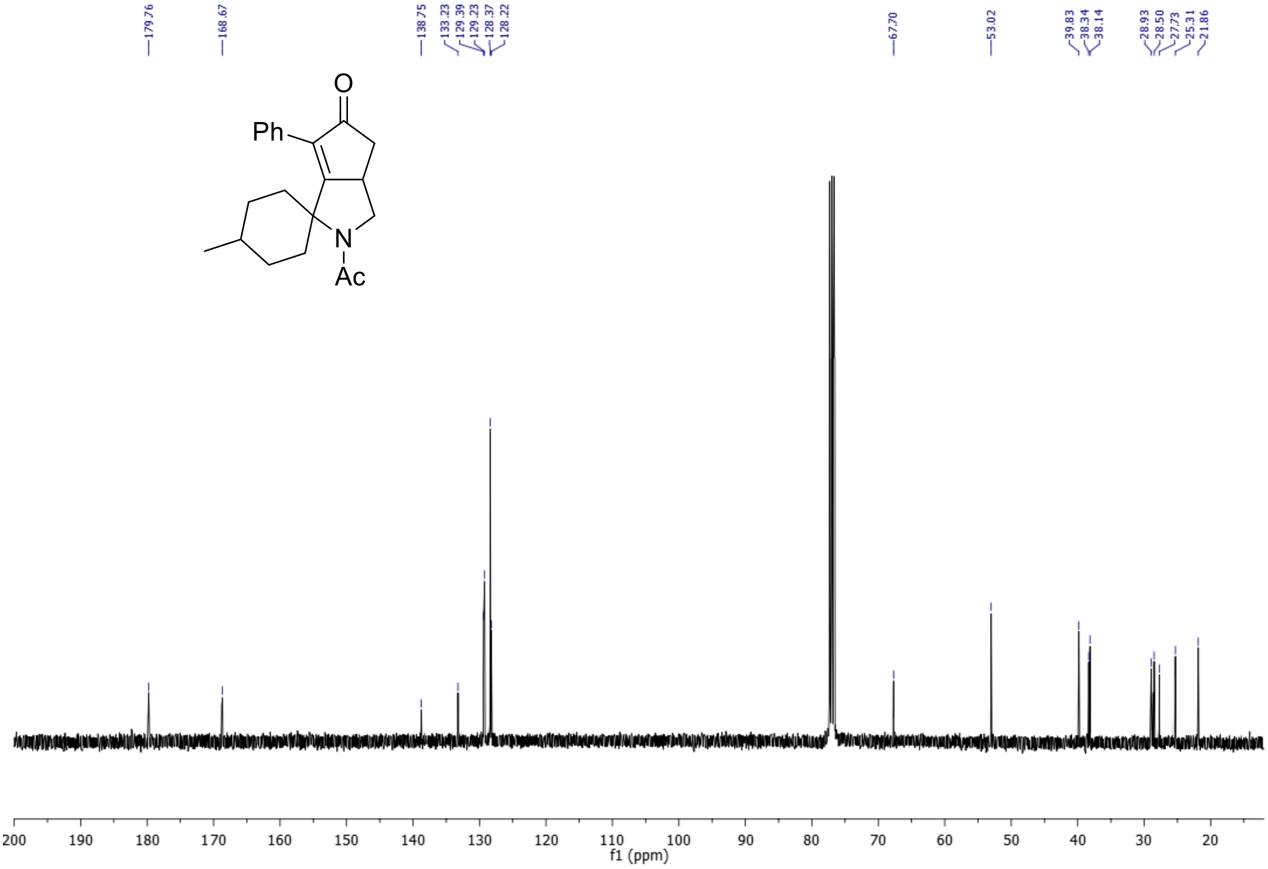

Figure S24. ^{13}C NMR spectrum of compound **19** (100 MHz, $CDCl_3$).

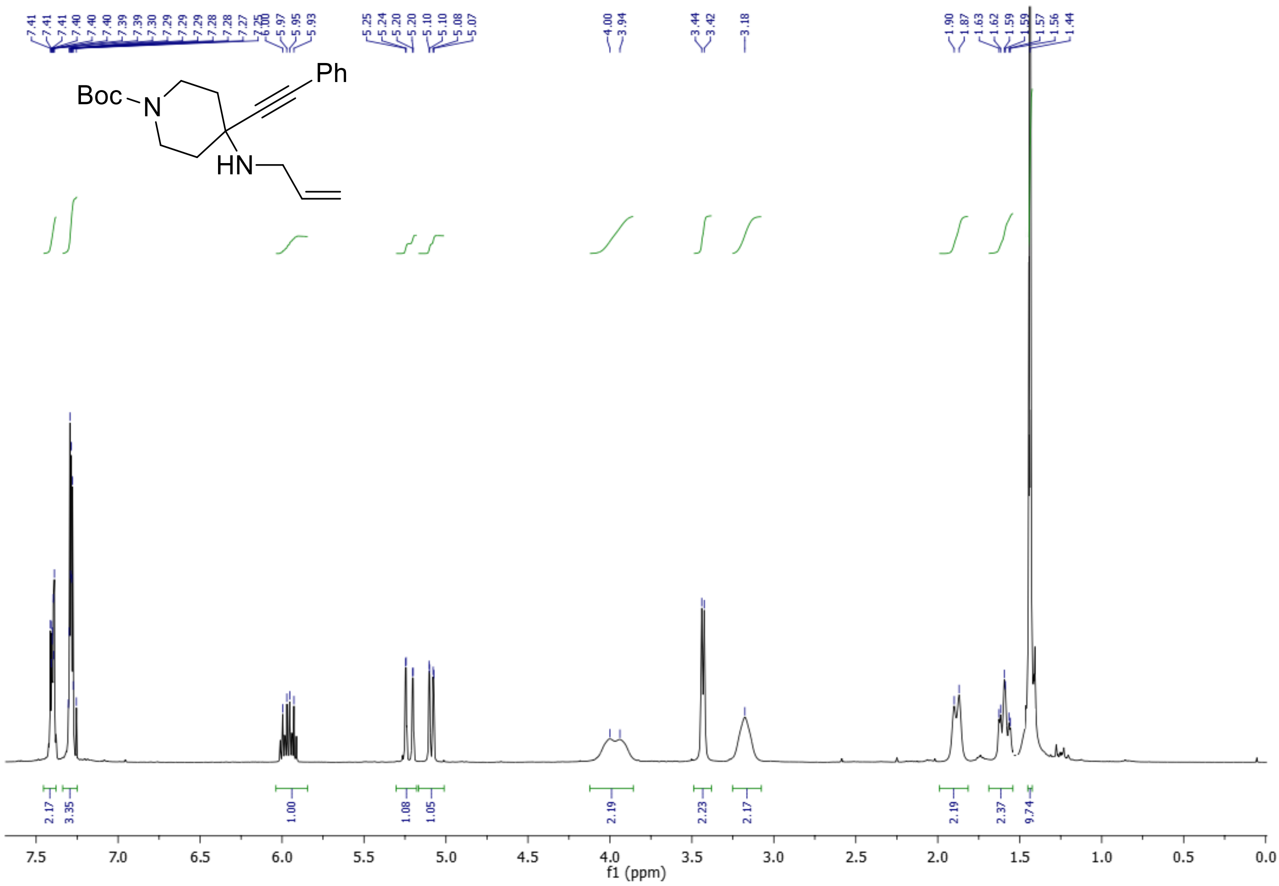

Figure S25. ^{1}H NMR spectrum of compound **24** (400 MHz, $CDCl_3$).

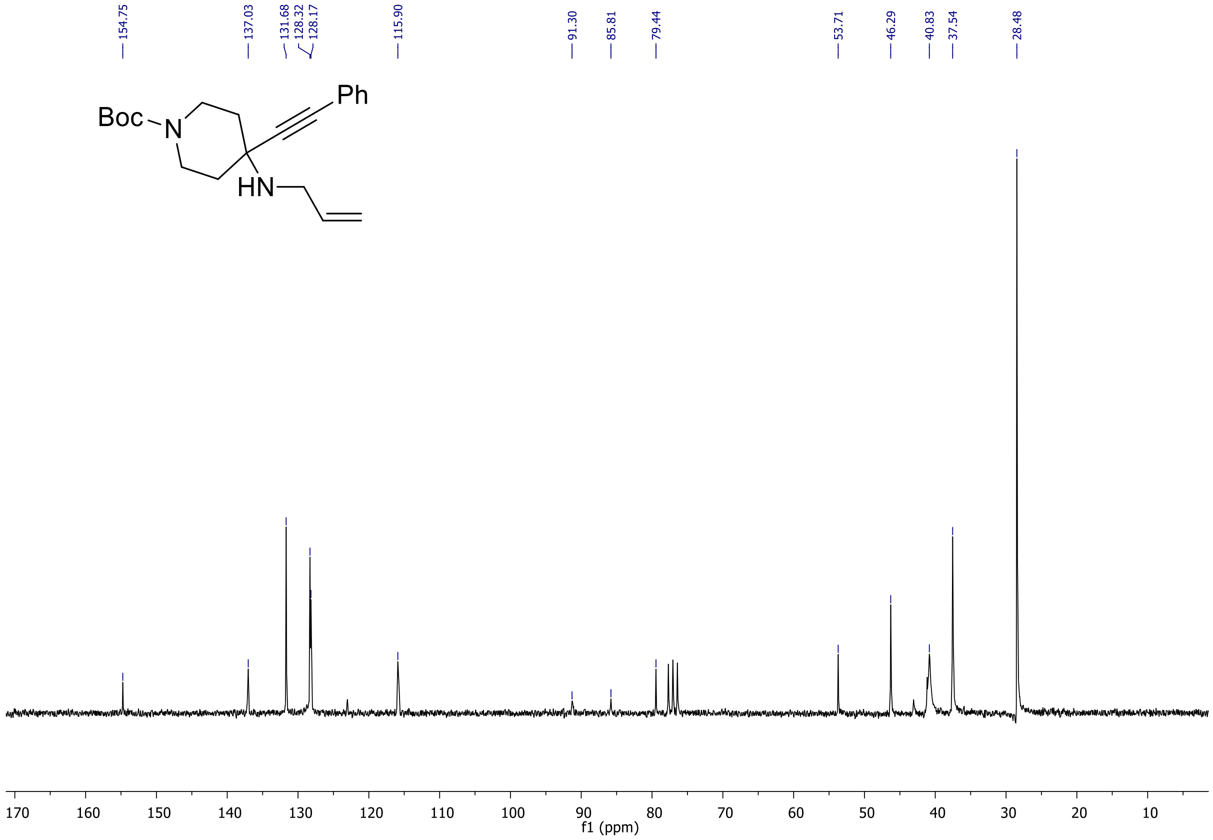

Figure S26. ^{13}C NMR spectrum of compound **24** (50 MHz, $CDCl_3$).

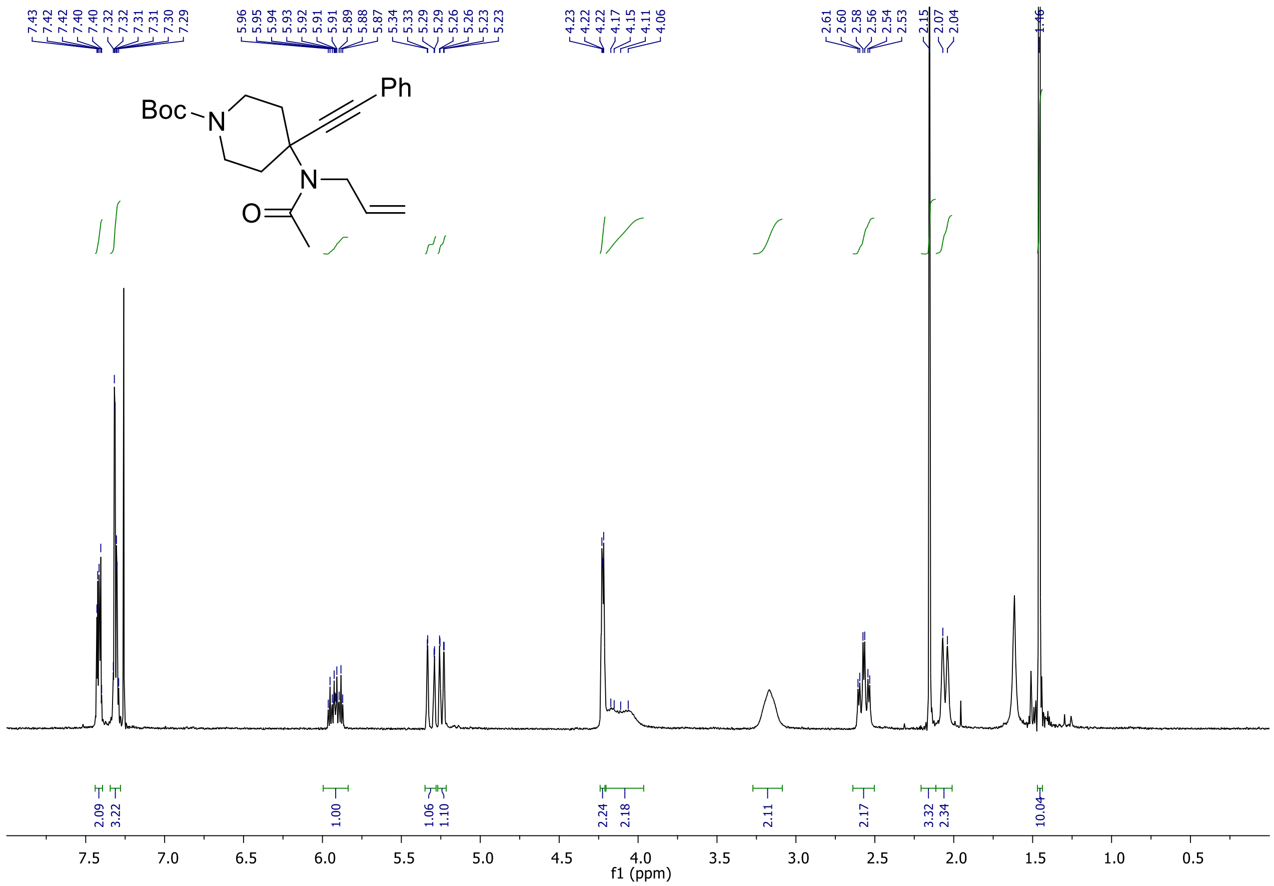

Figure S27. ^{1}H NMR spectrum of compound **25** (400 MHz, $CDCl_3$).

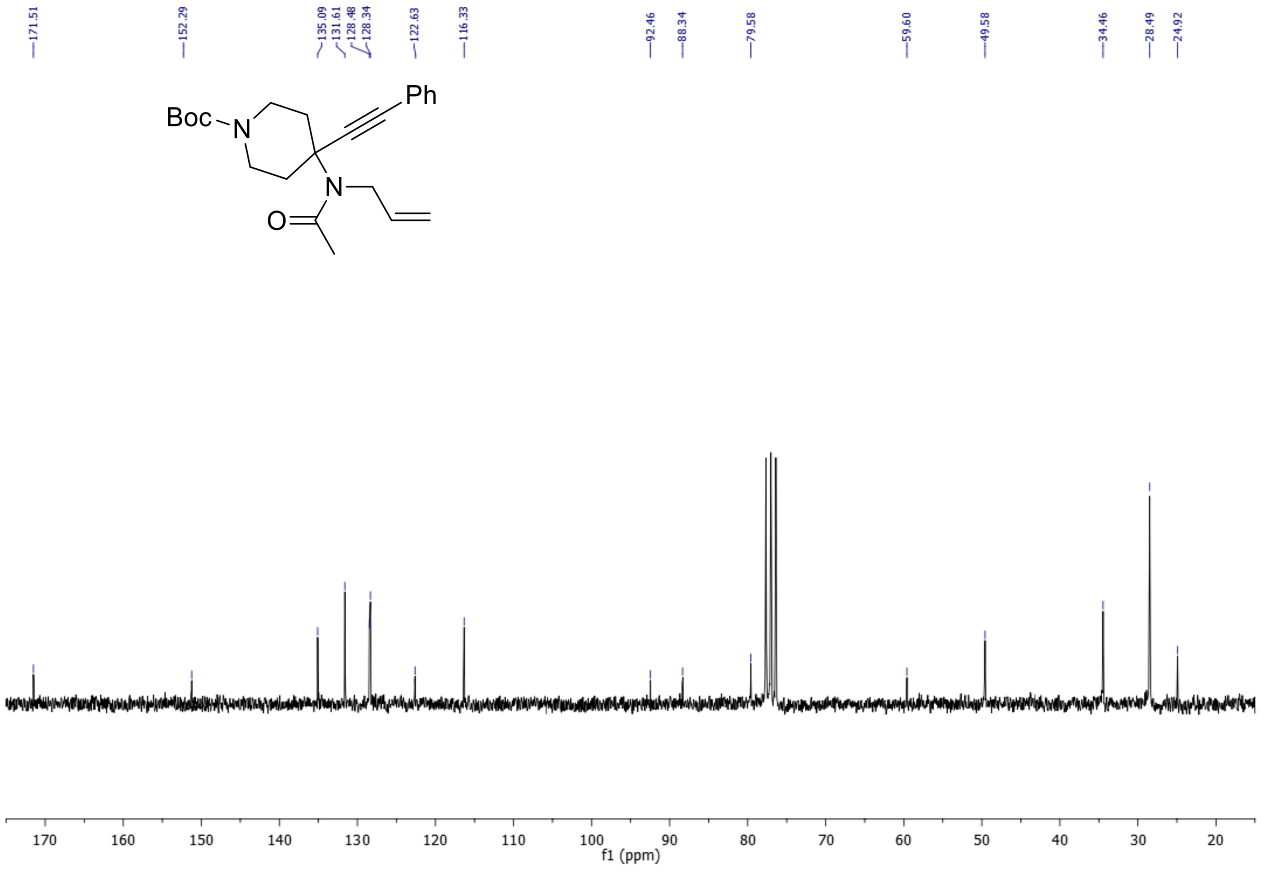

Figure S28. ^{13}C NMR spectrum of compound **25** (50 MHz, $CDCl_3$).

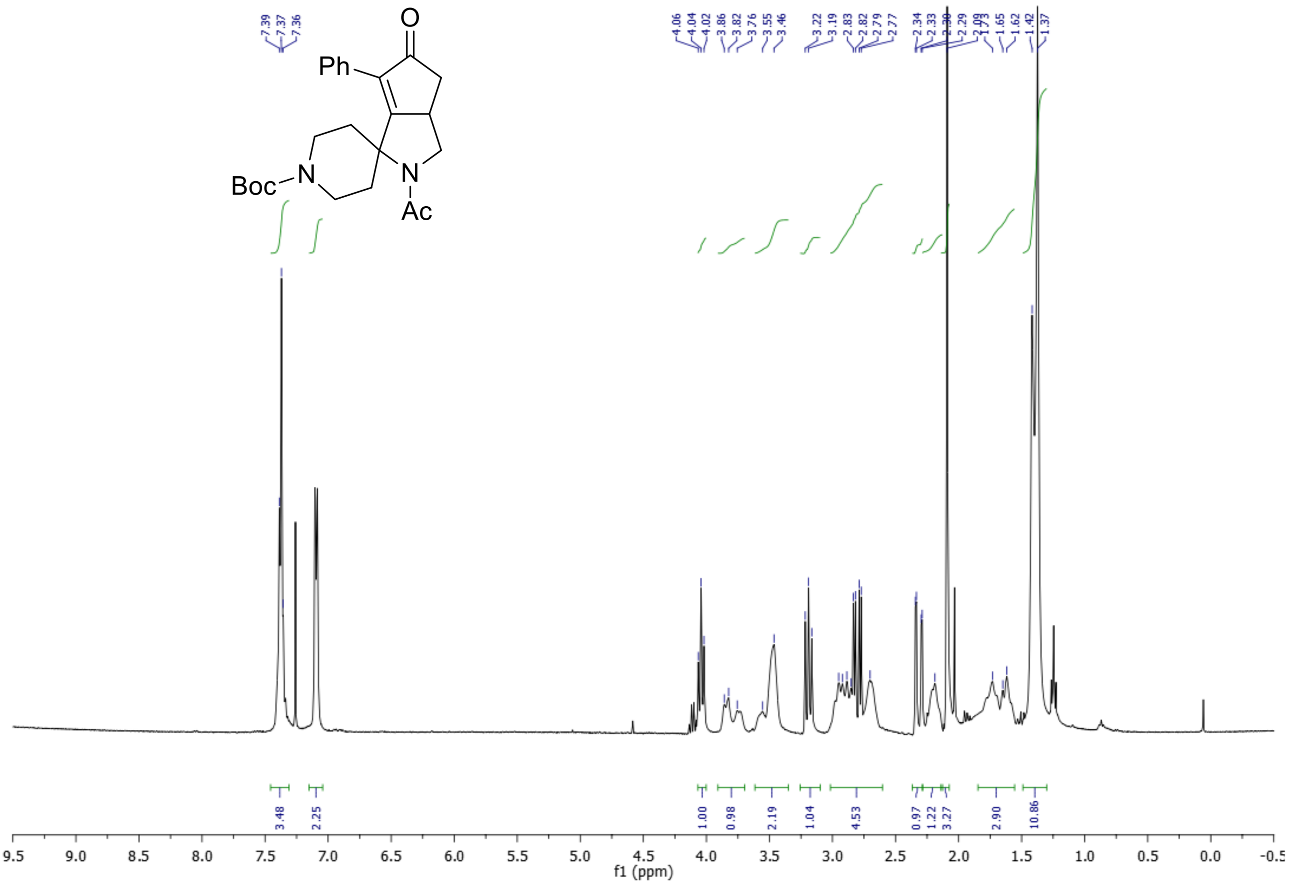

Figure S29. ^{1}H NMR spectrum of compound **26** (400 MHz, $CDCl_3$).

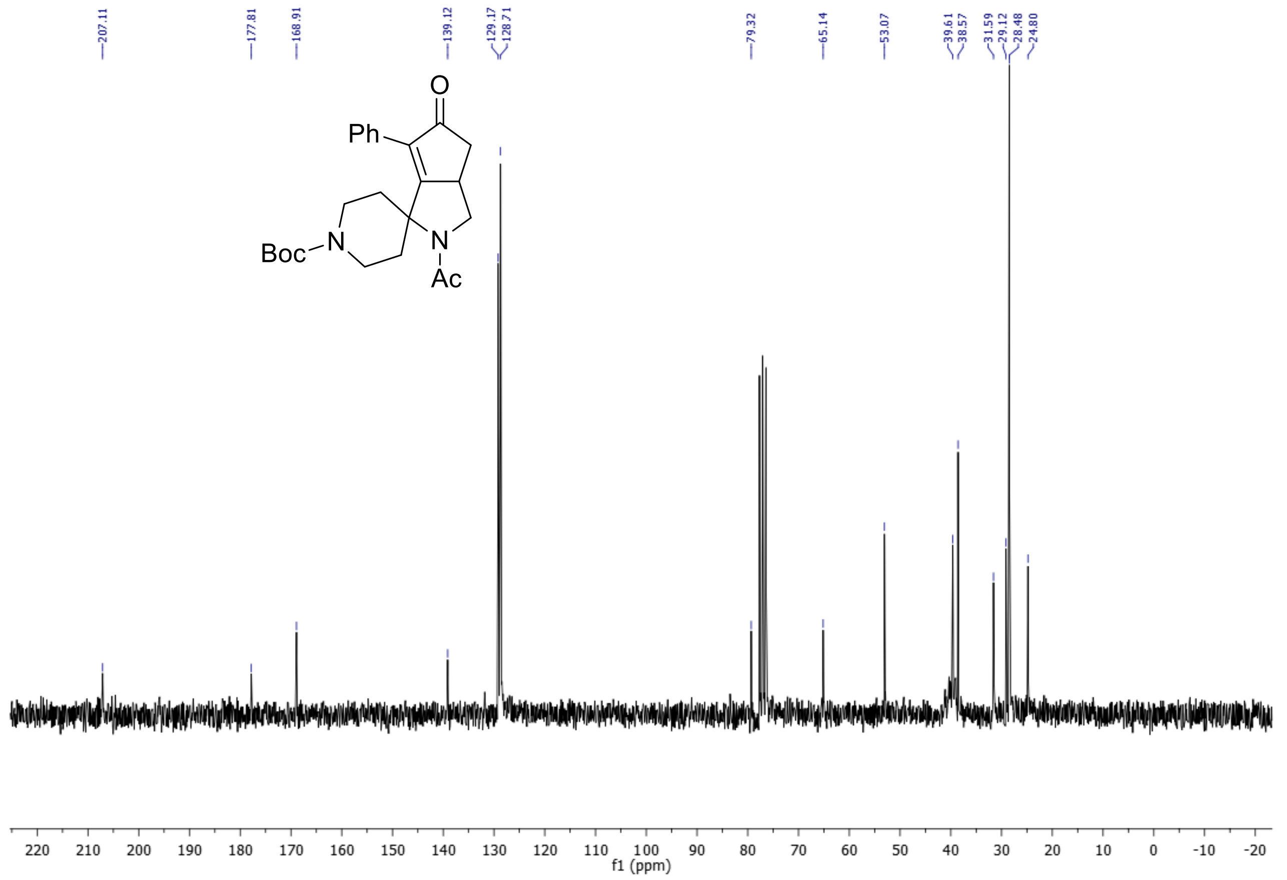

Figure S30. ^{13}C NMR spectrum of compound **26** (50 MHz, $CDCl_3$).

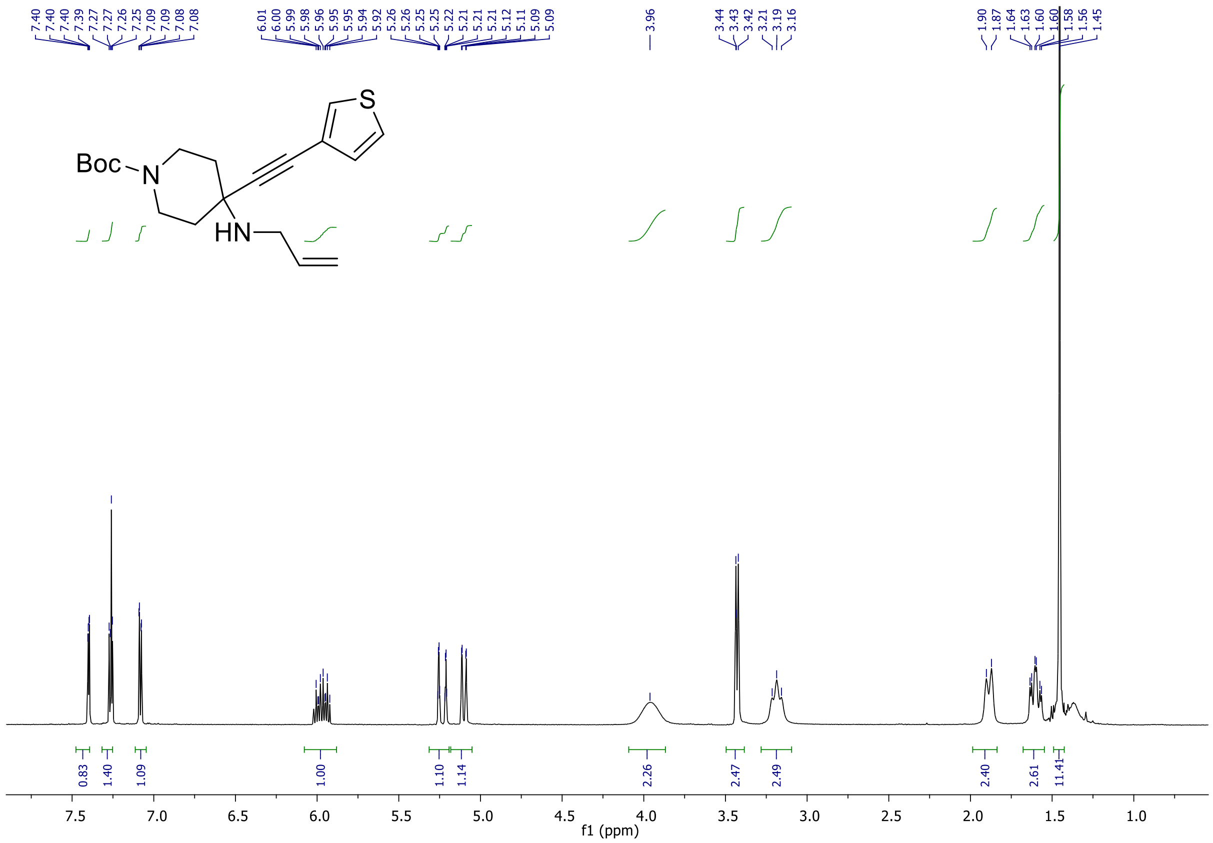

Figure S31. ^{1}H NMR spectrum of compound **27** (400 MHz, $CDCl_3$).

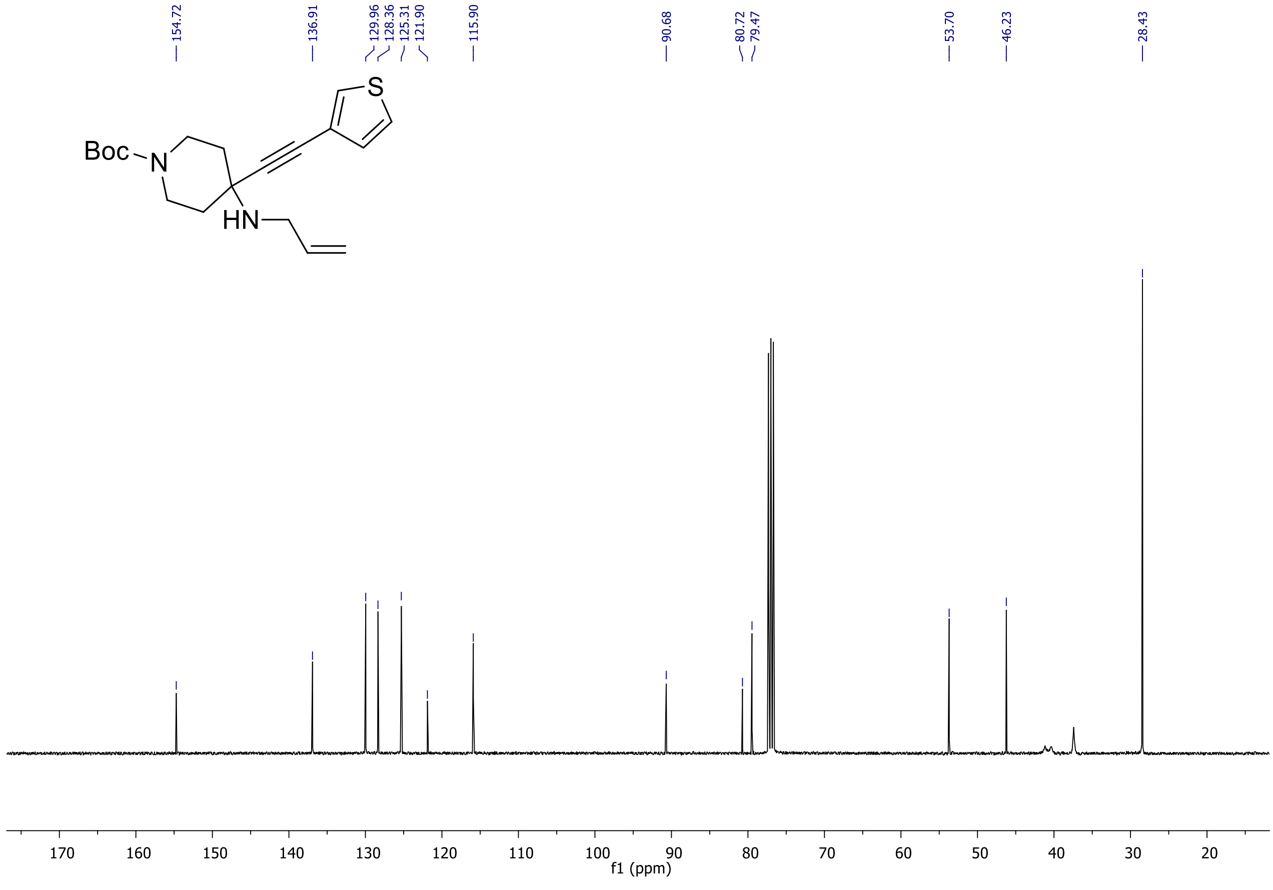

Figure S32. ^{13}C NMR spectrum of compound **27** (100 MHz, $CDCl_3$).

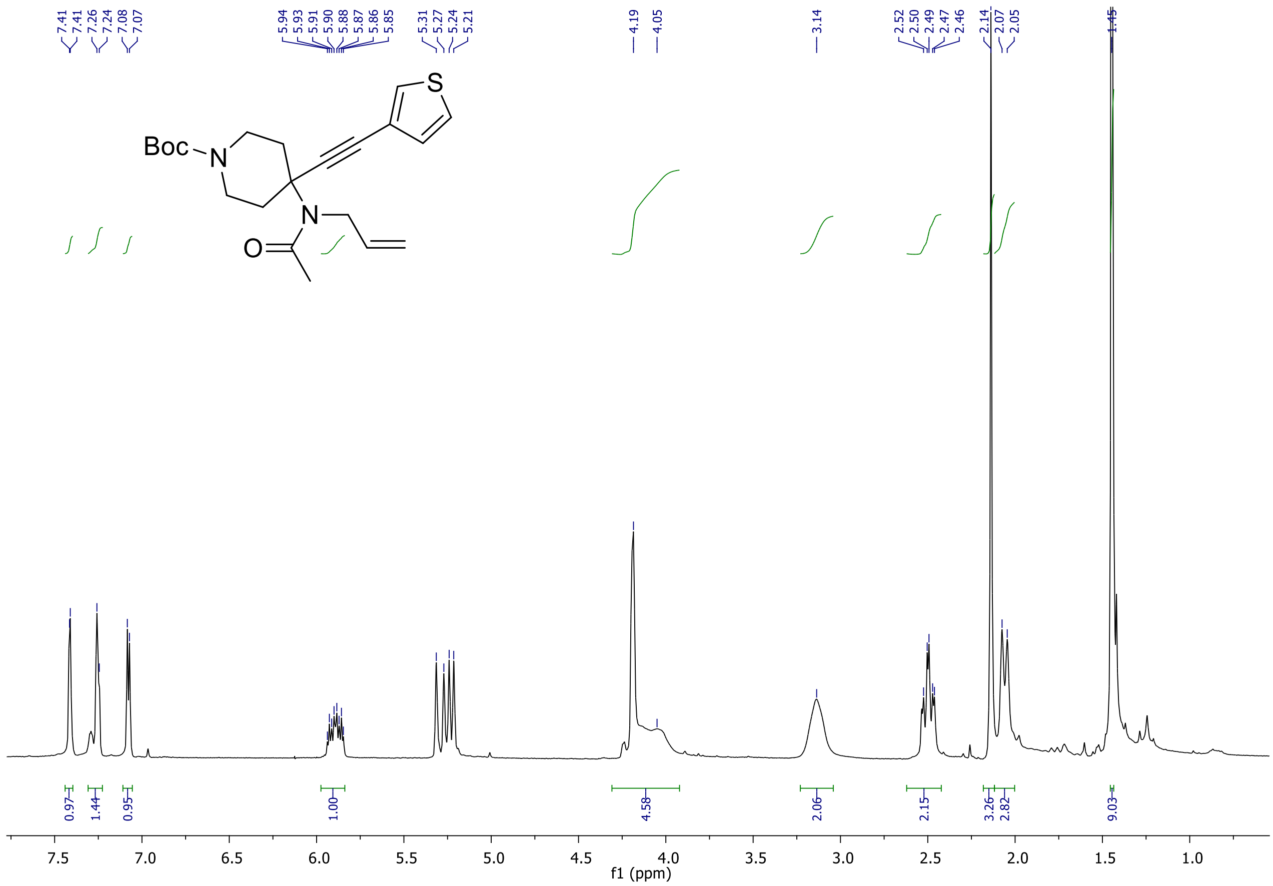

Figure S33. ^{1}H NMR spectrum of compound **28** (400 MHz, $CDCl_3$).

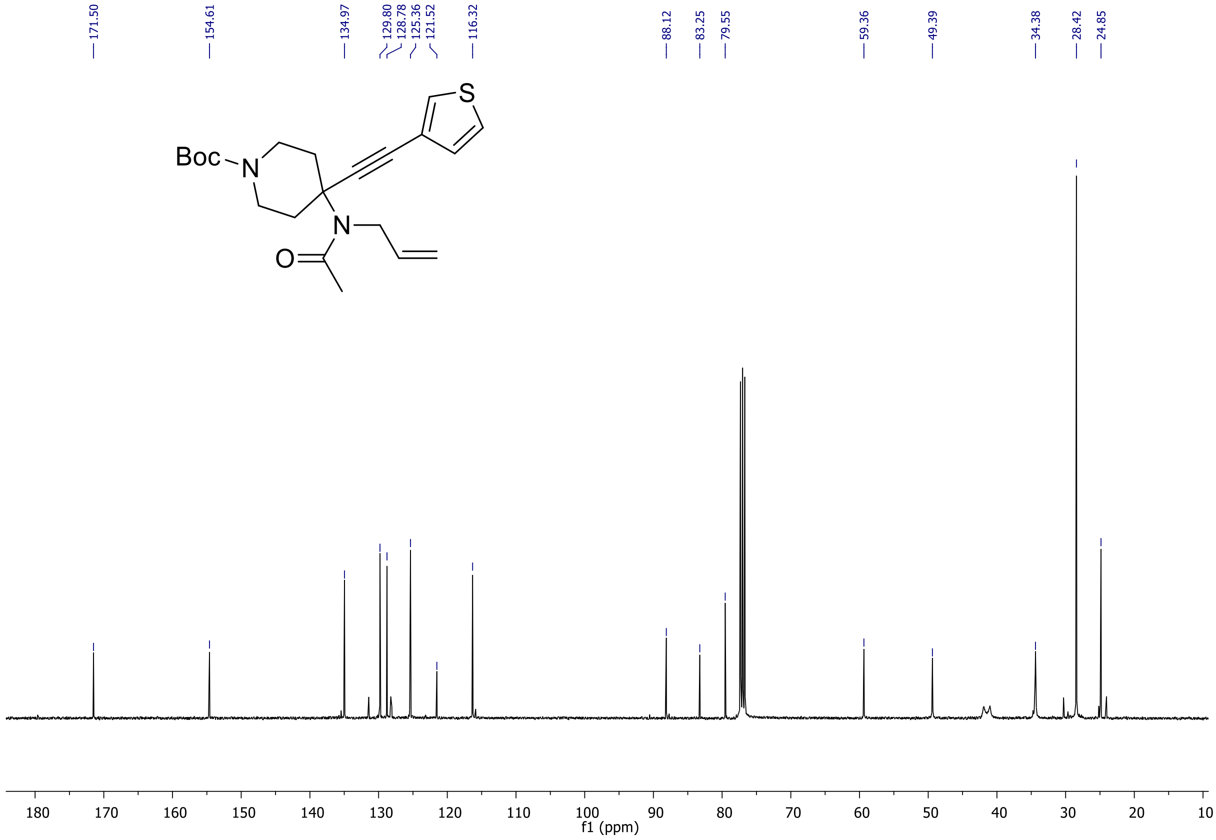

Figure S34. ^{13}C NMR spectrum of compound **28** (100 MHz, $CDCl_3$).

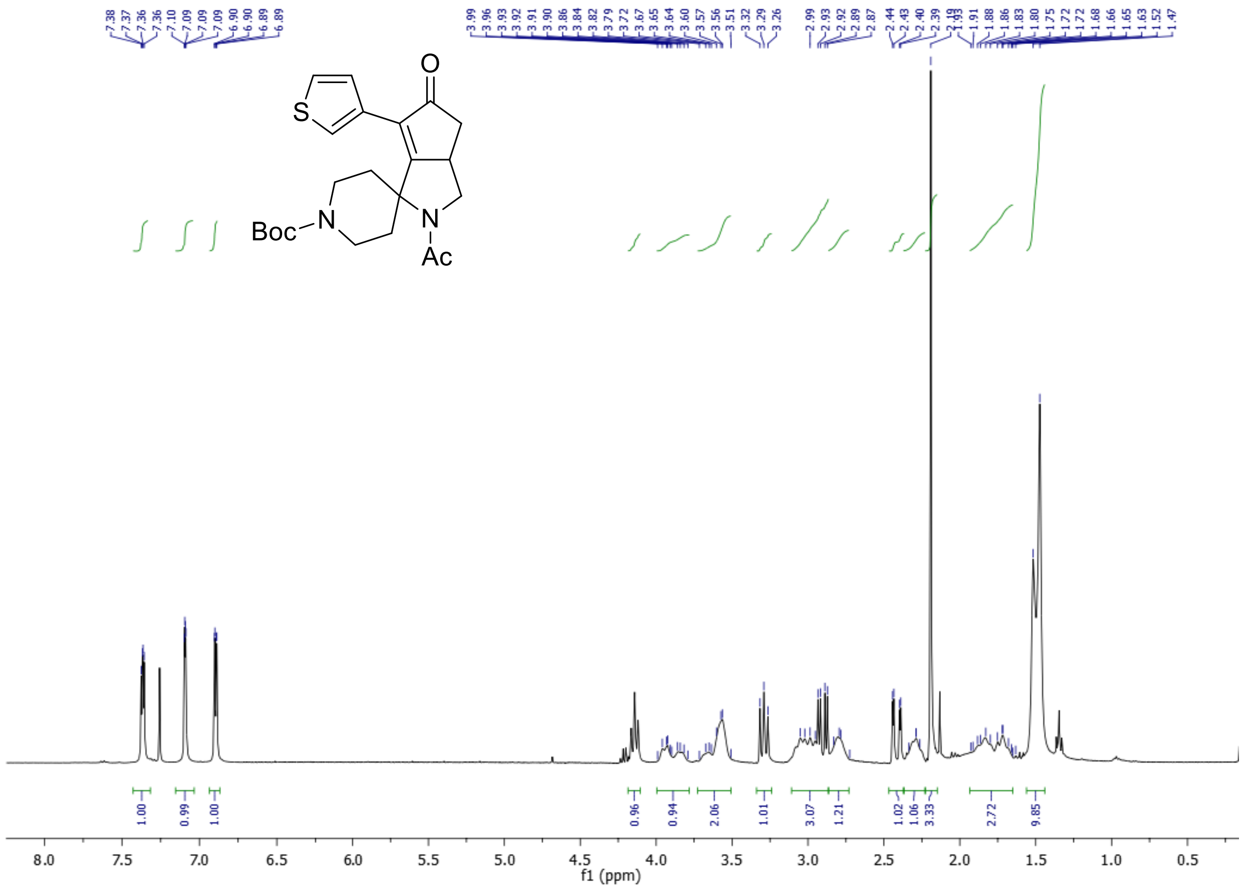

Figure S35. ^{1}H NMR spectrum of compound **29** (400 MHz, $CDCl_3$).

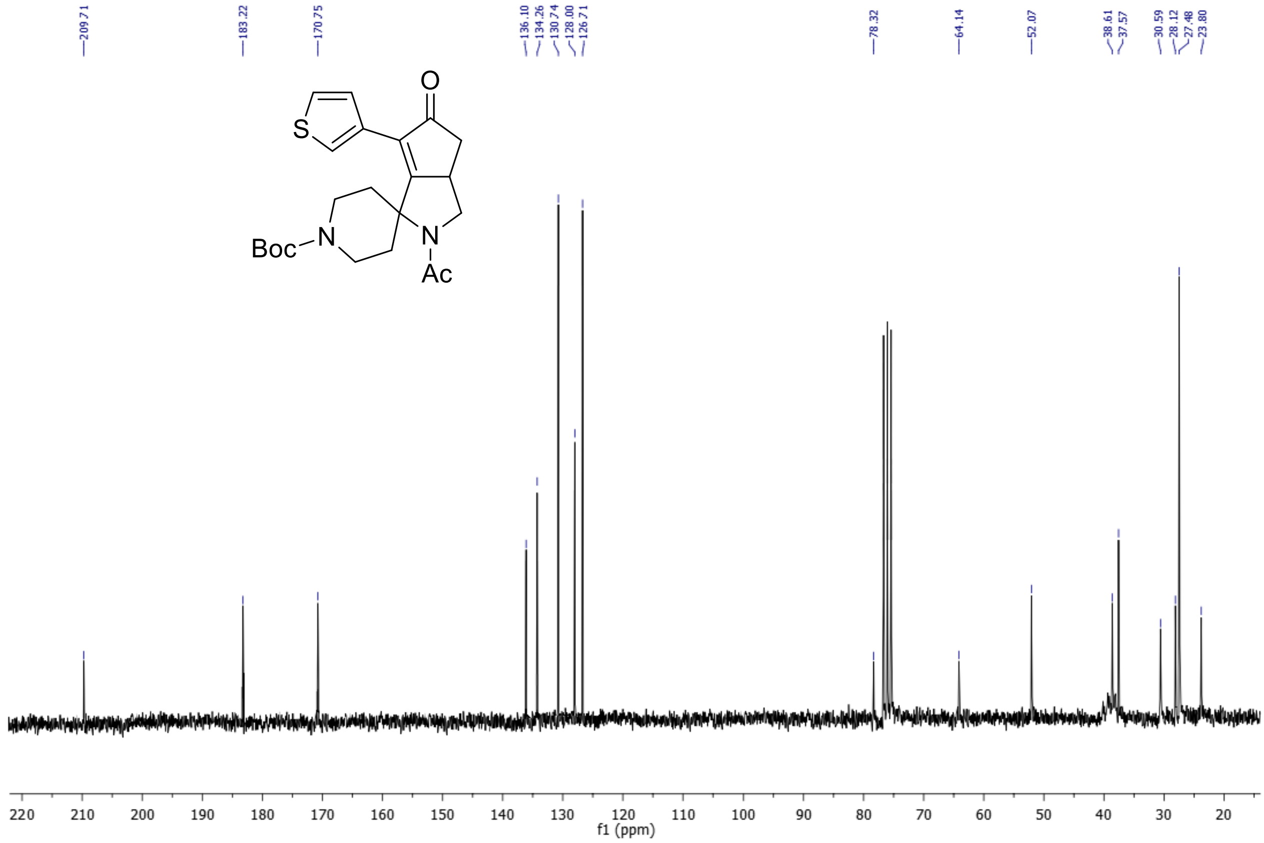

Figure S36. ^{13}C NMR spectrum of compound **29** (100 MHz, $CDCl_3$).

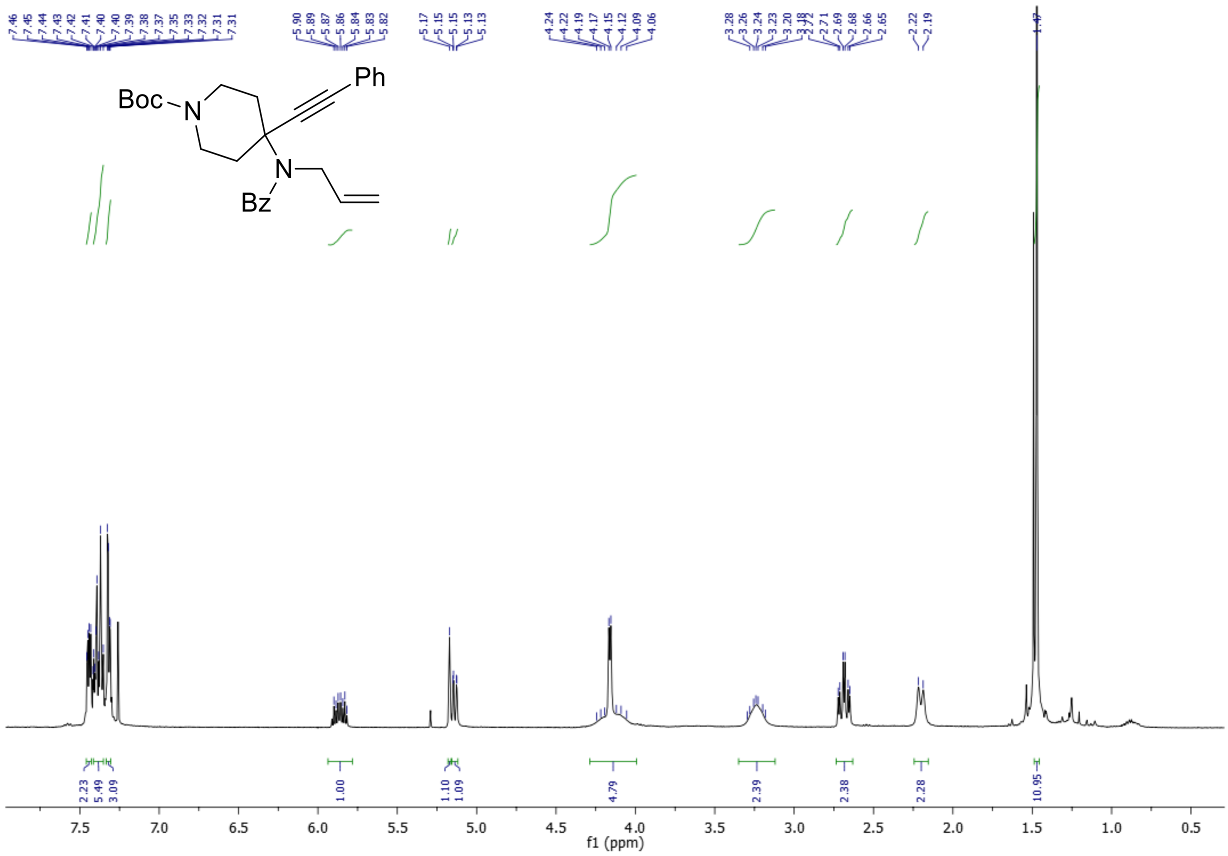

Figure S37. ^{1}H NMR spectrum of compound **30** (400 MHz, $CDCl_3$).

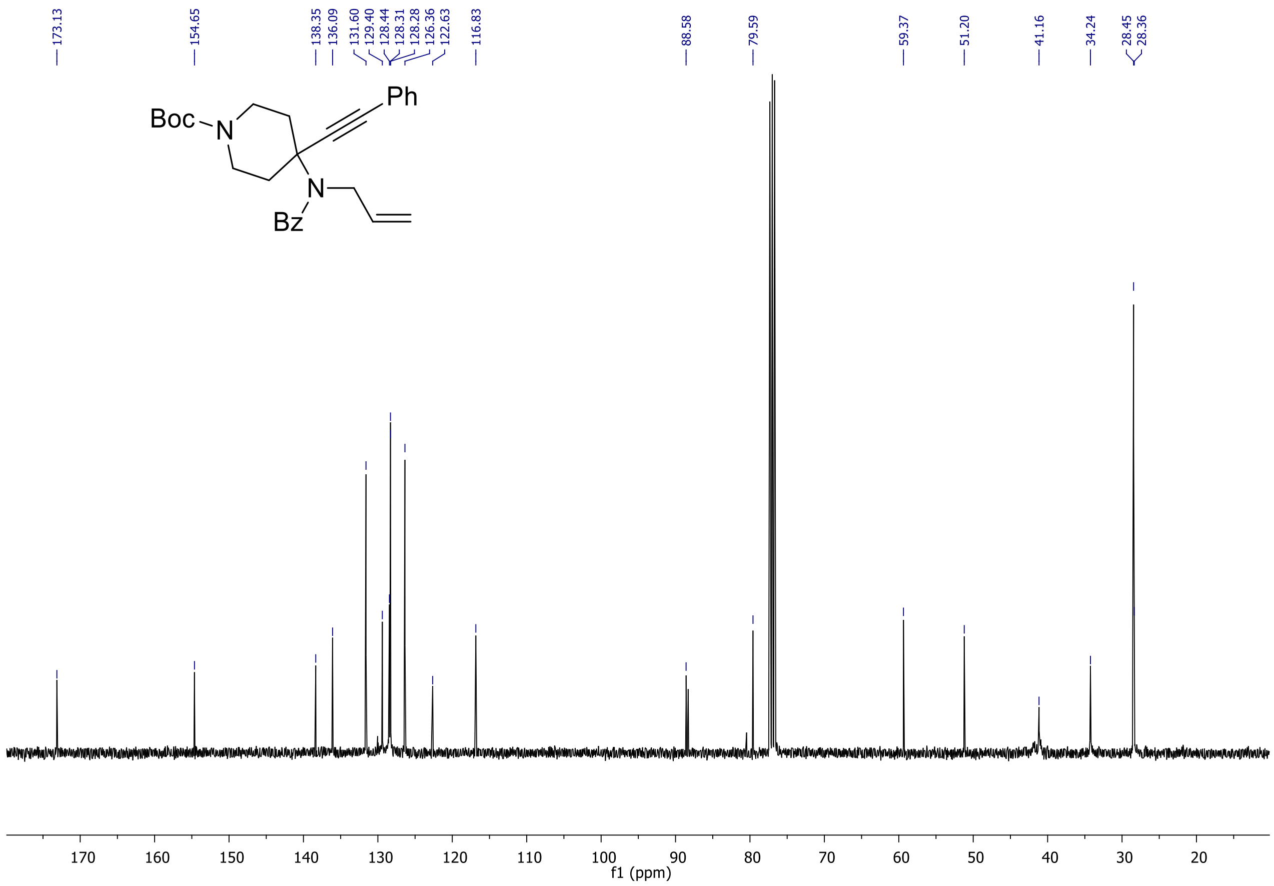

Figure S38. ^{13}C NMR spectrum of compound **30** (100 MHz, $CDCl_3$).

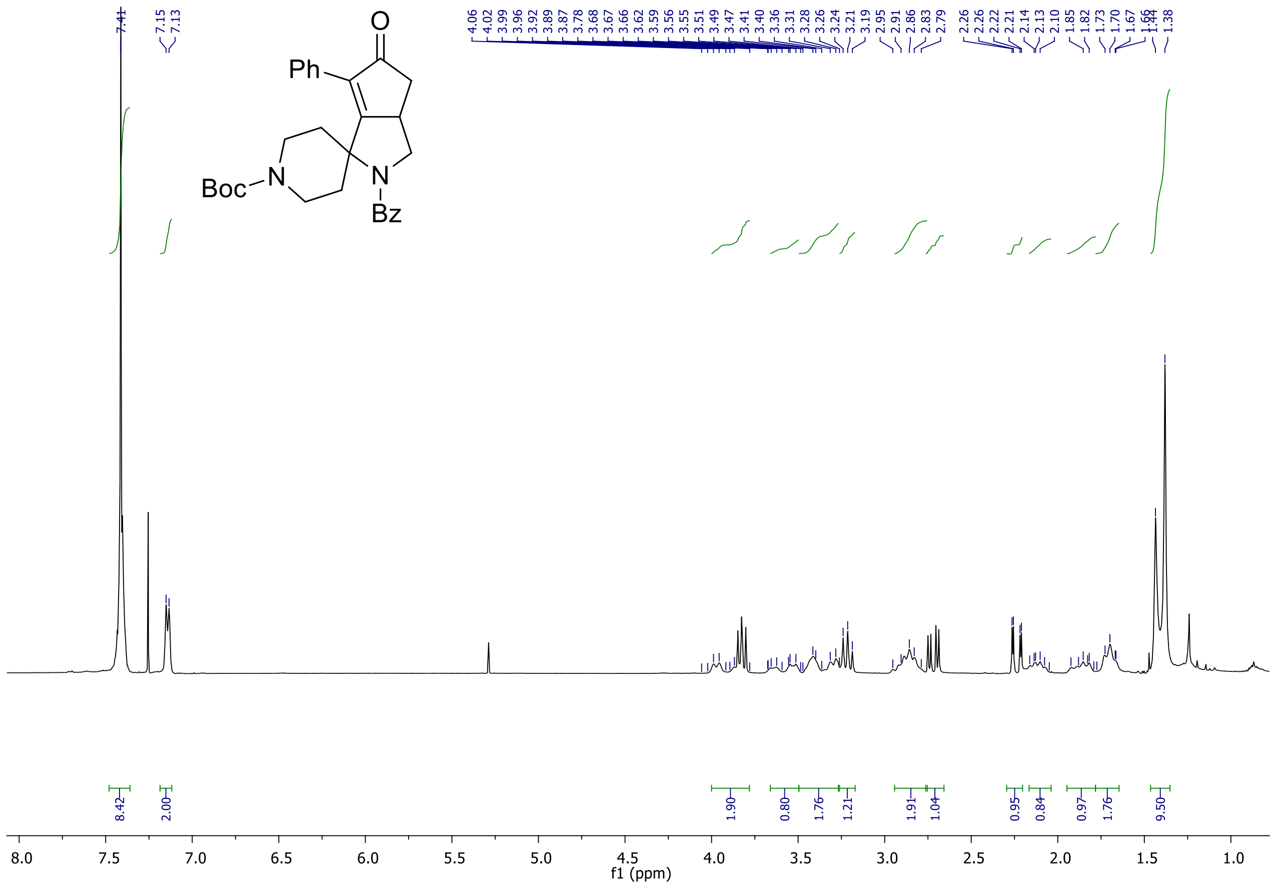

Figure S39. ^{1}H NMR spectrum of compound **31** (400 MHz, $CDCl_3$).

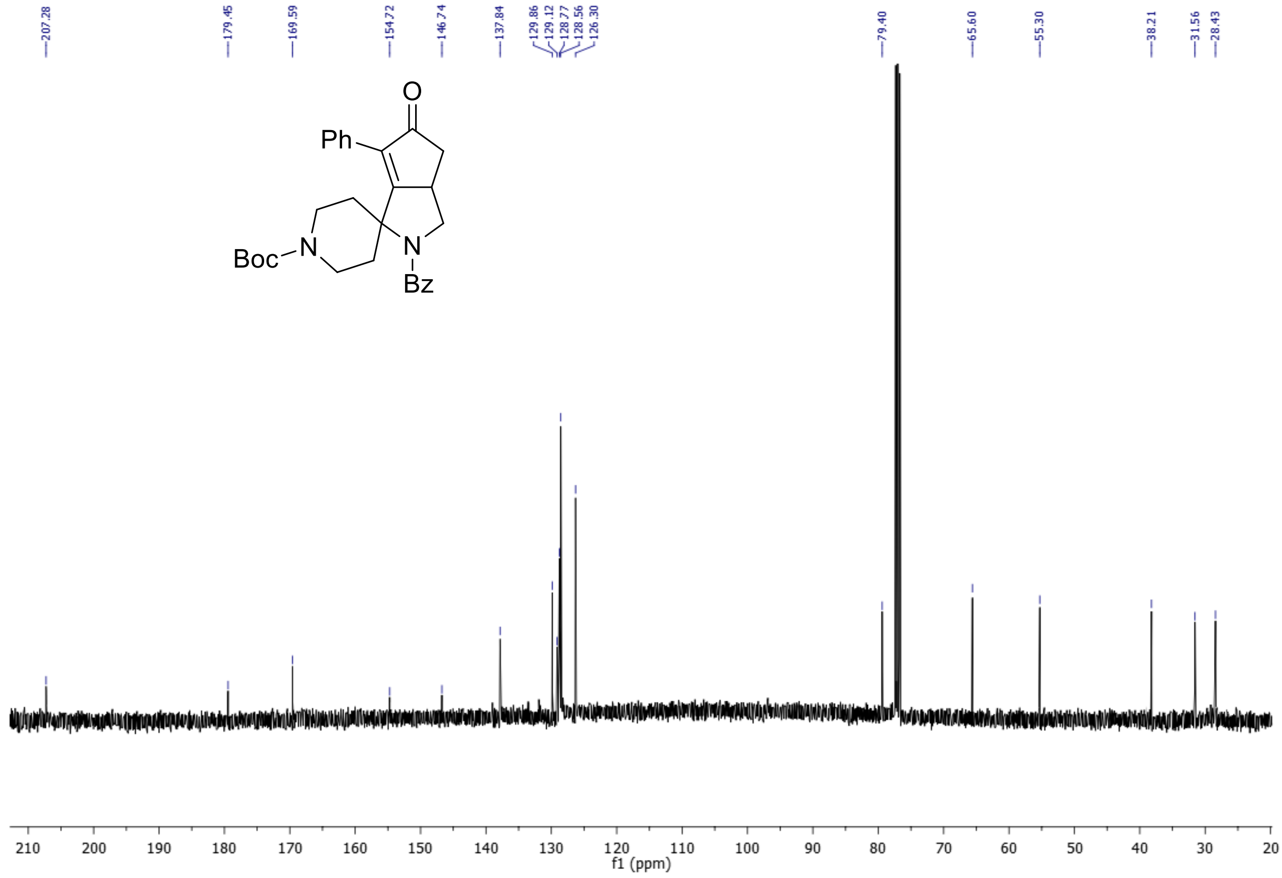

Figure S40. ^{13}C NMR spectrum of compound **31** (100 MHz, $CDCl_3$).

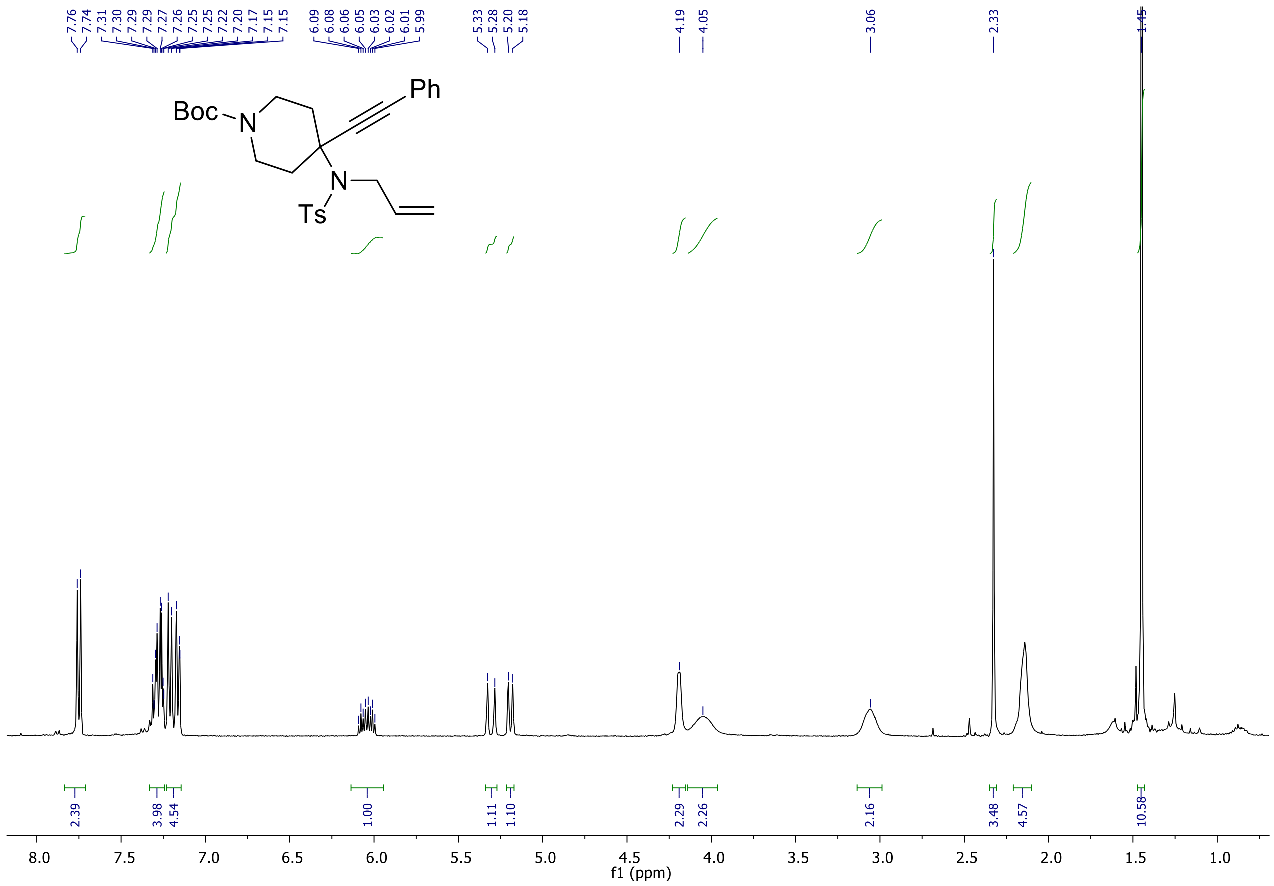

Figure S41. ^{1}H NMR spectrum of compound **32** (400 MHz, $CDCl_3$).

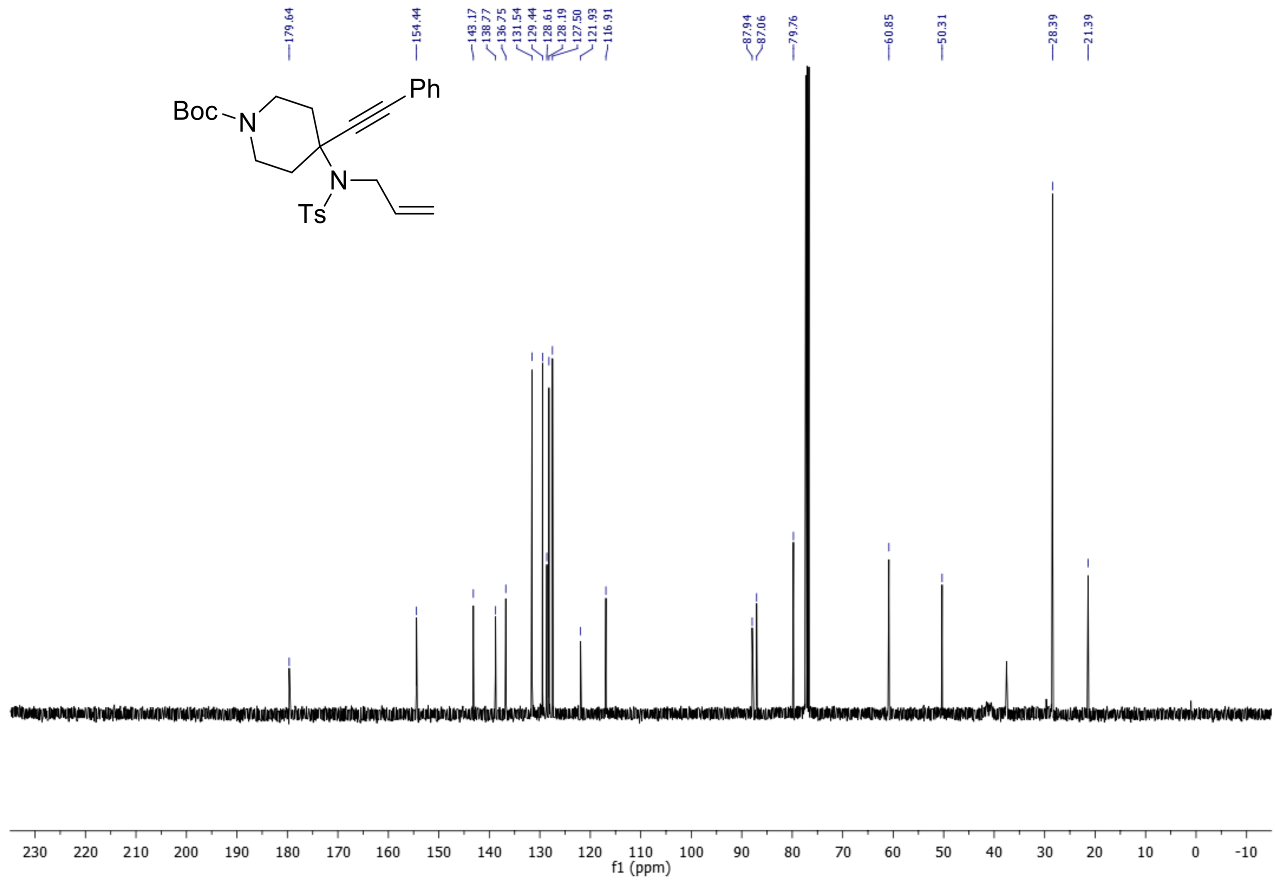

Figure S42. ^{13}C NMR spectrum of compound **32** (100 MHz, $CDCl_3$).

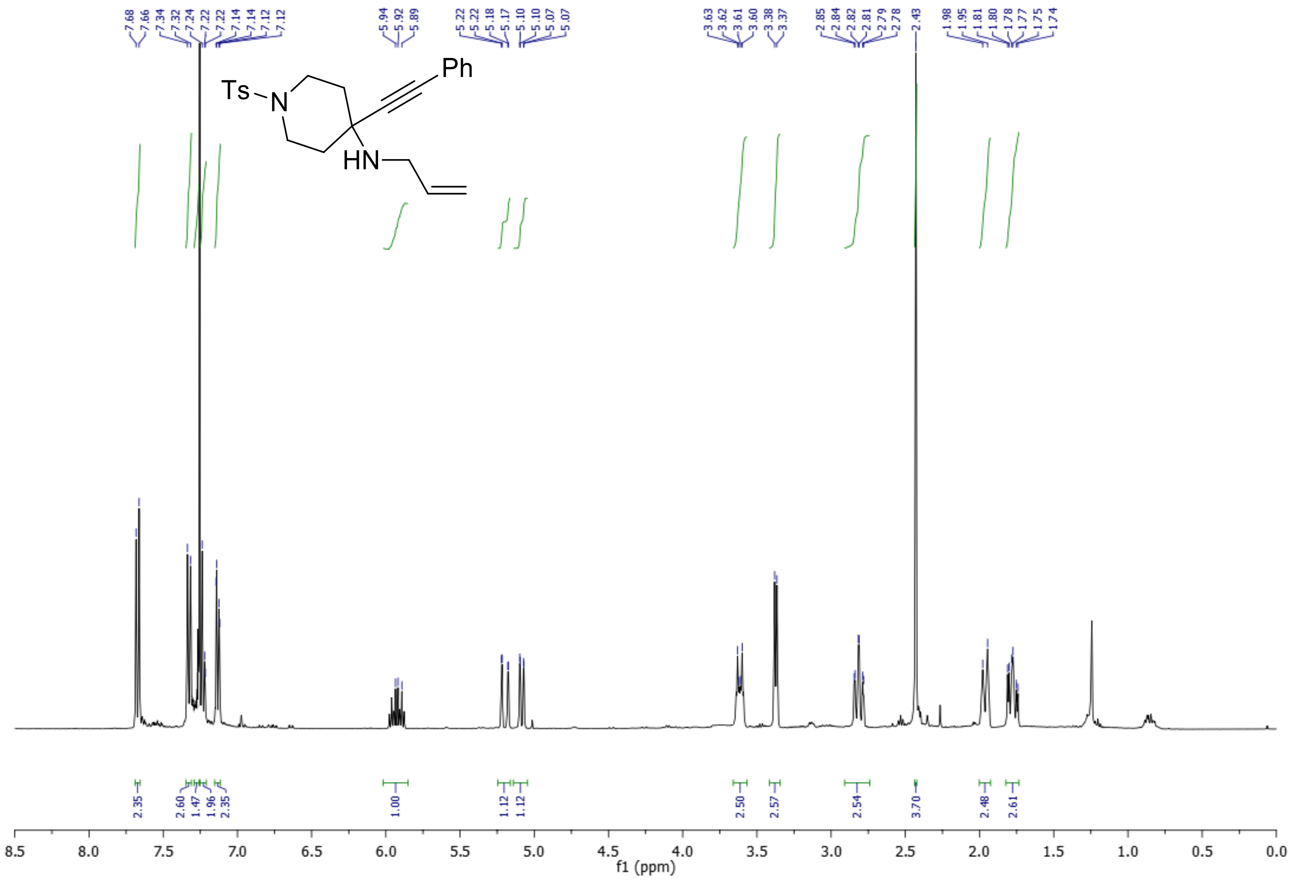

Figure S43. ^{1}H NMR spectrum of compound **34** (400 MHz, $CDCl_3$).

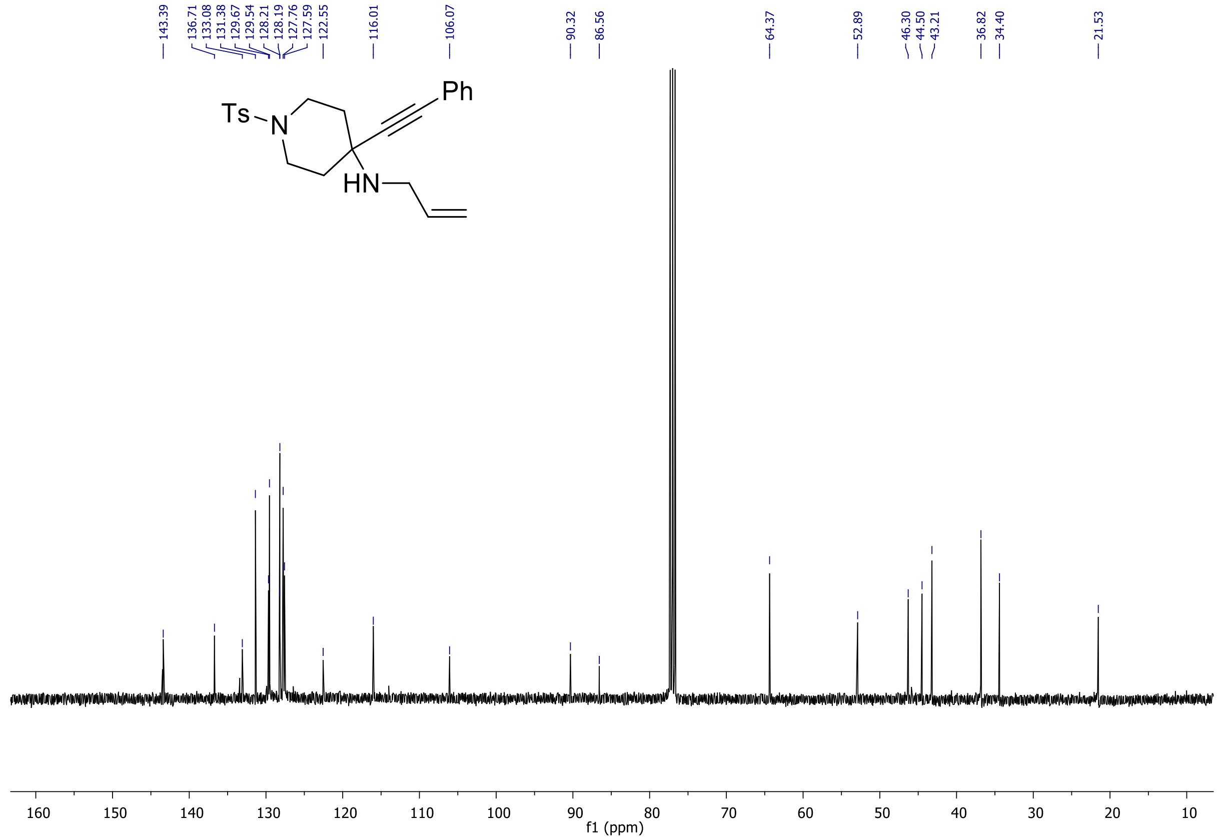

Figure S44. ^{13}C NMR spectrum of compound **34** (100 MHz, $CDCl_3$).

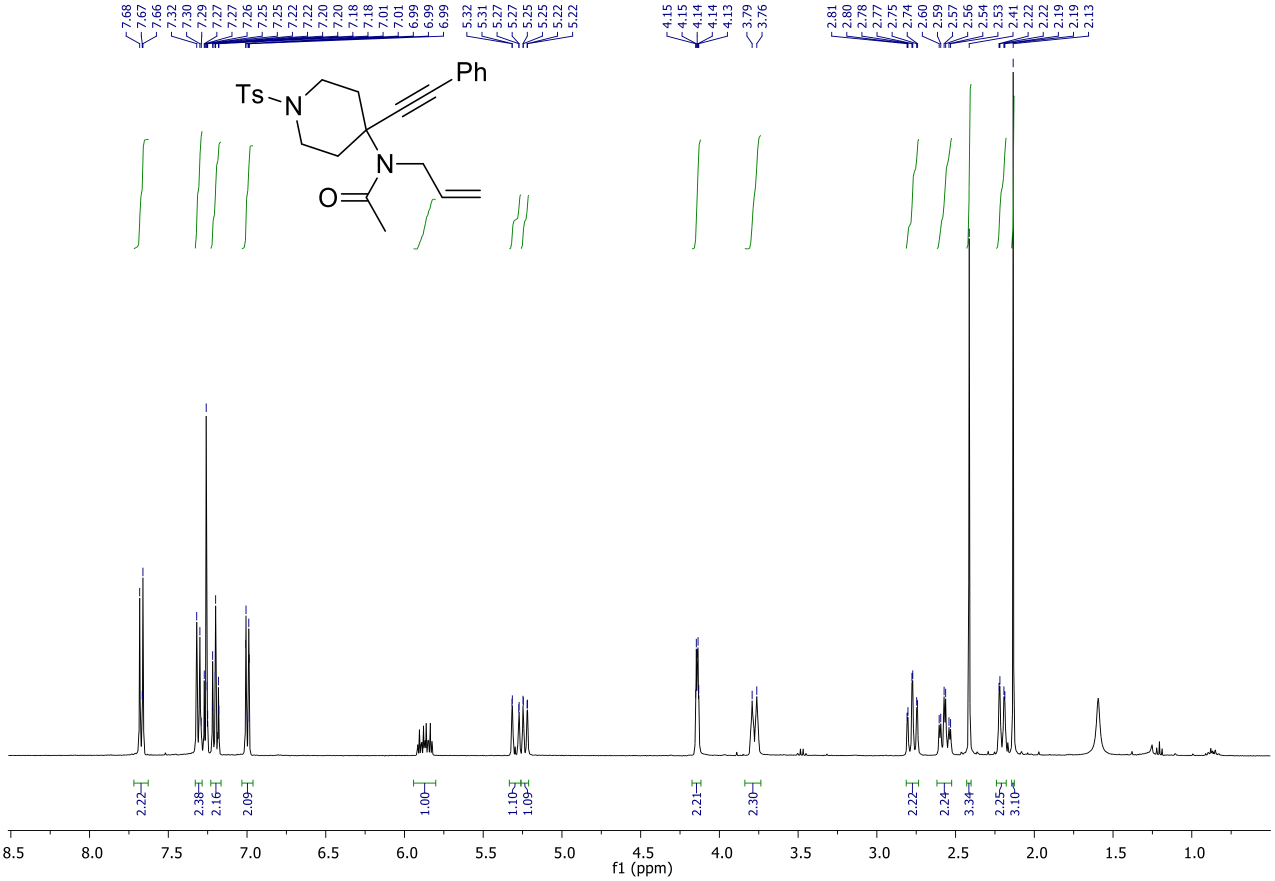

Figure S45. ^{1}H NMR spectrum of compound **35** (400 MHz, $CDCl_3$).

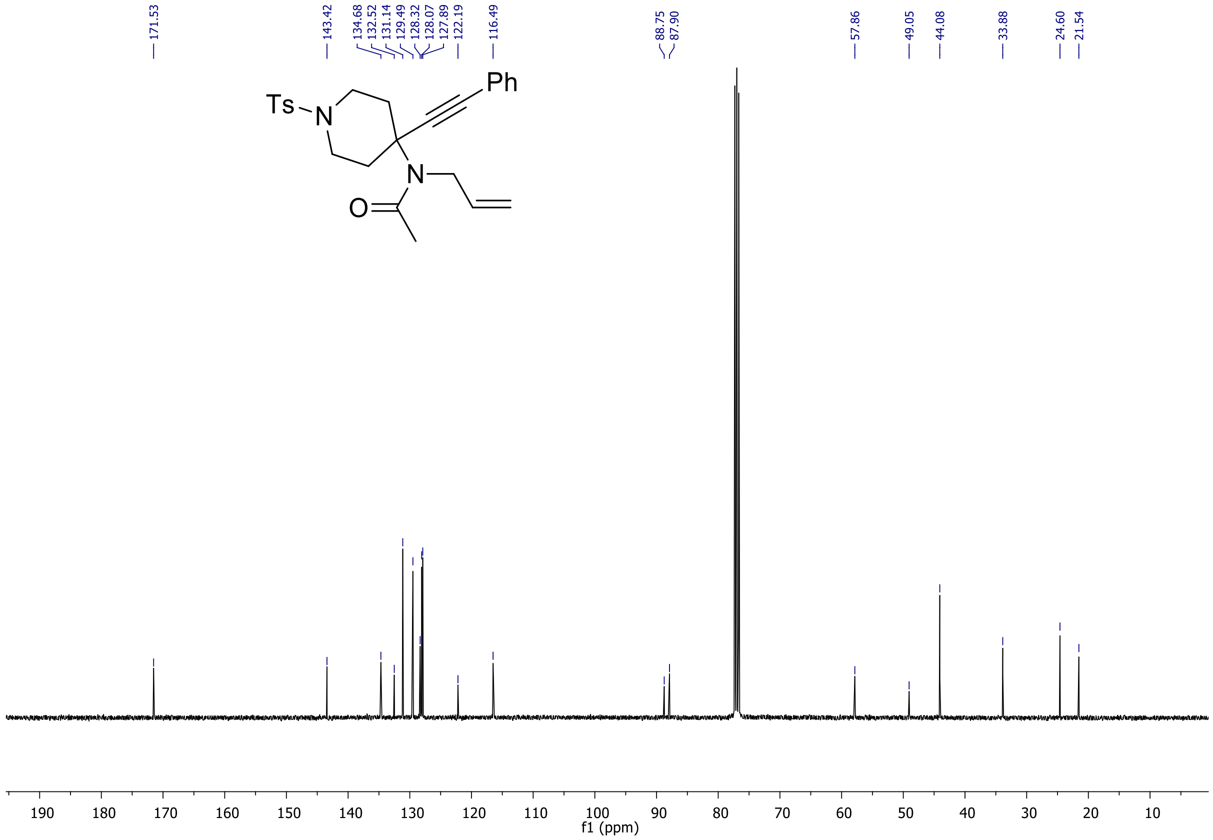

Figure S46. ^{13}C NMR spectrum of compound **35** (100 MHz, $CDCl_3$).

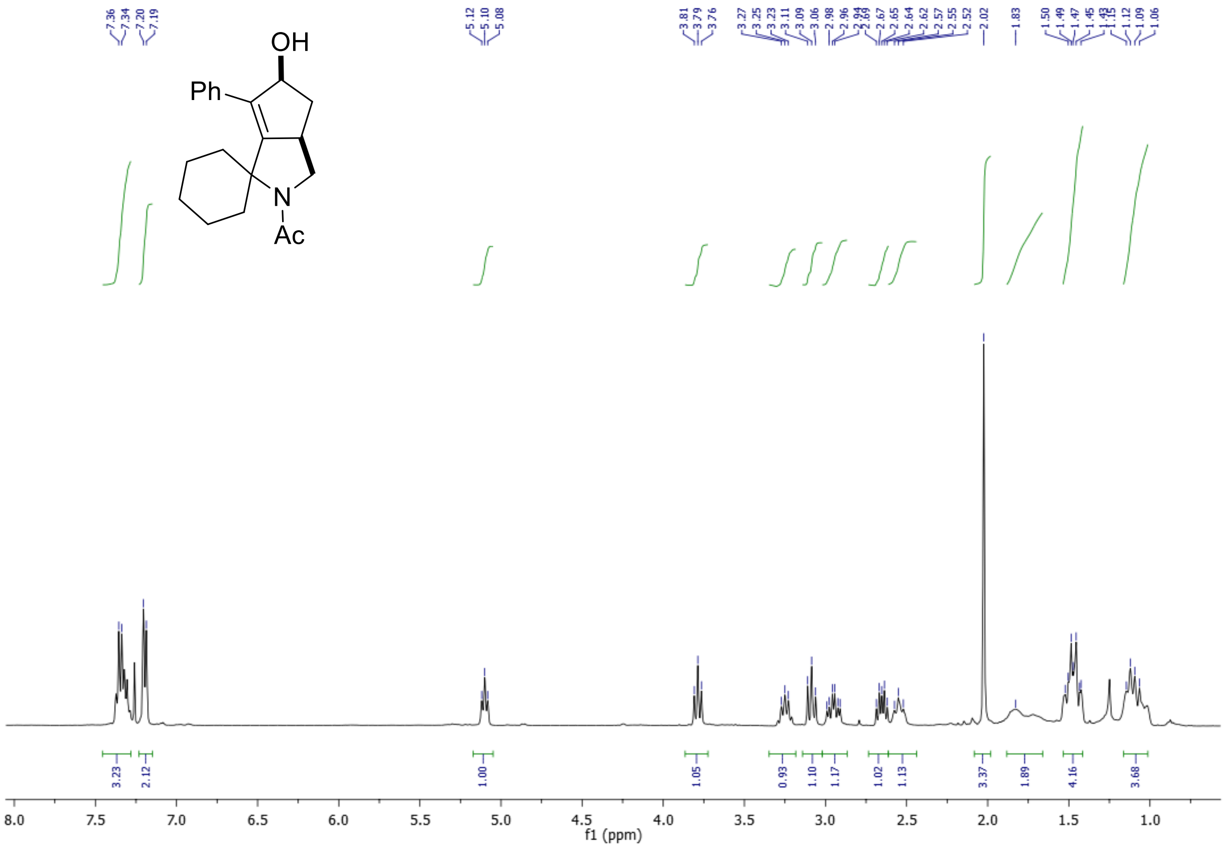

Figure S47. ^{1}H NMR spectrum of compound **36** (400 MHz, $CDCl_3$).

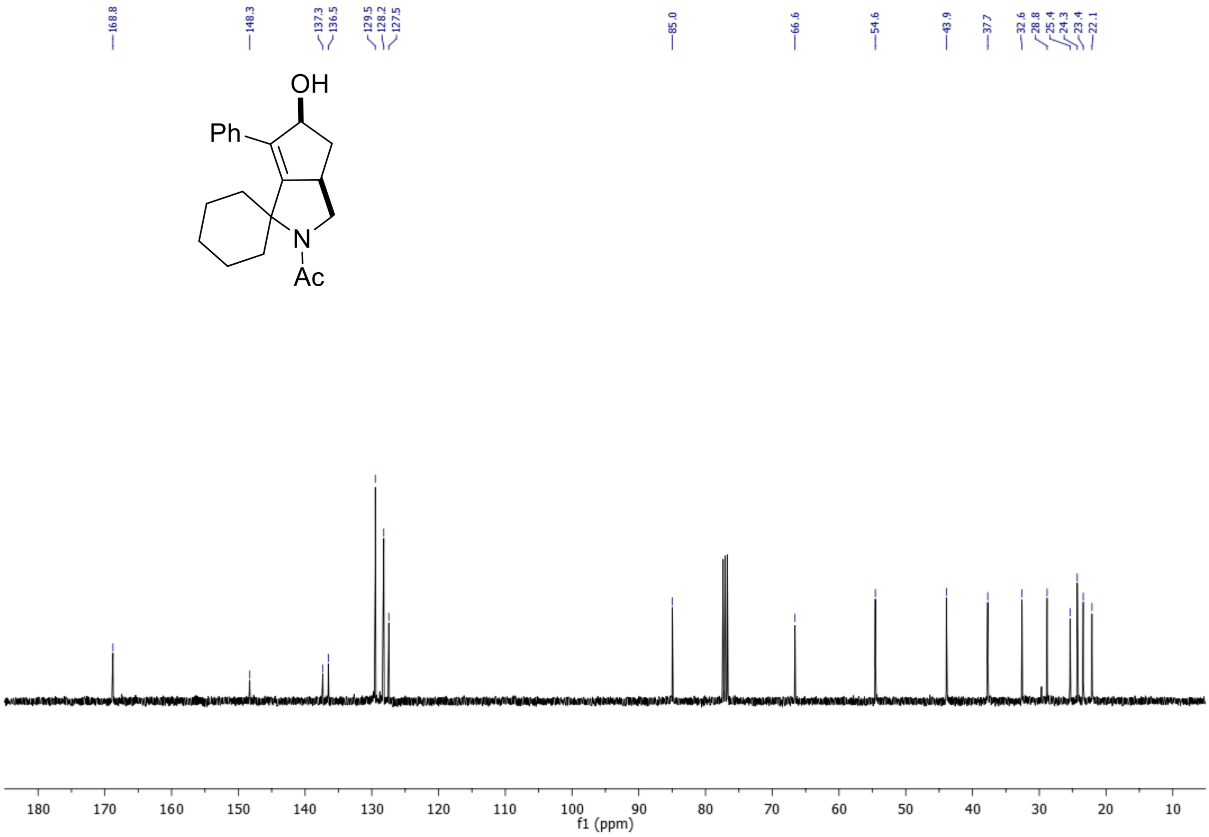

Figure S48. ^{13}C NMR spectrum of compound **36** (100 MHz, $CDCl_3$).

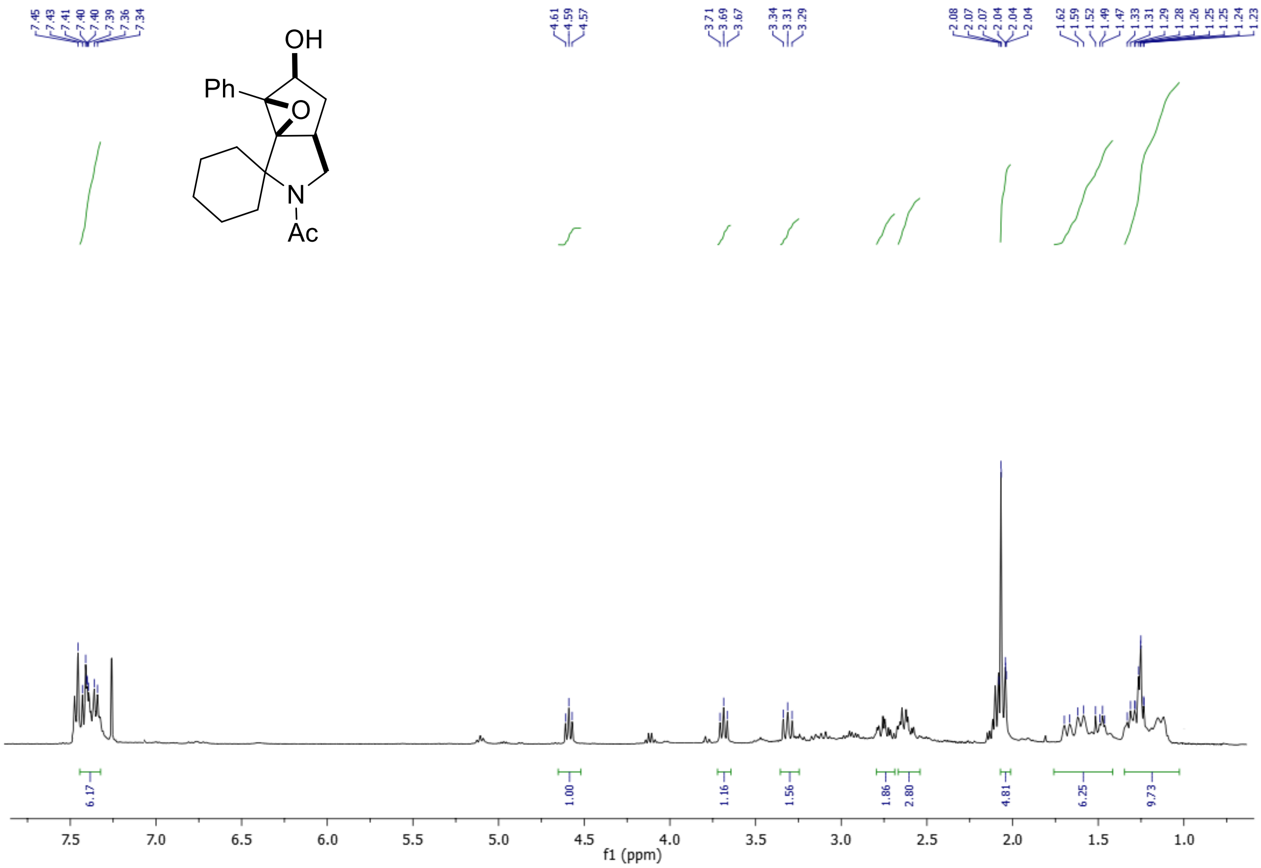

Figure S49. ^{1}H NMR spectrum of compound **37** (400 MHz, $CDCl_3$).

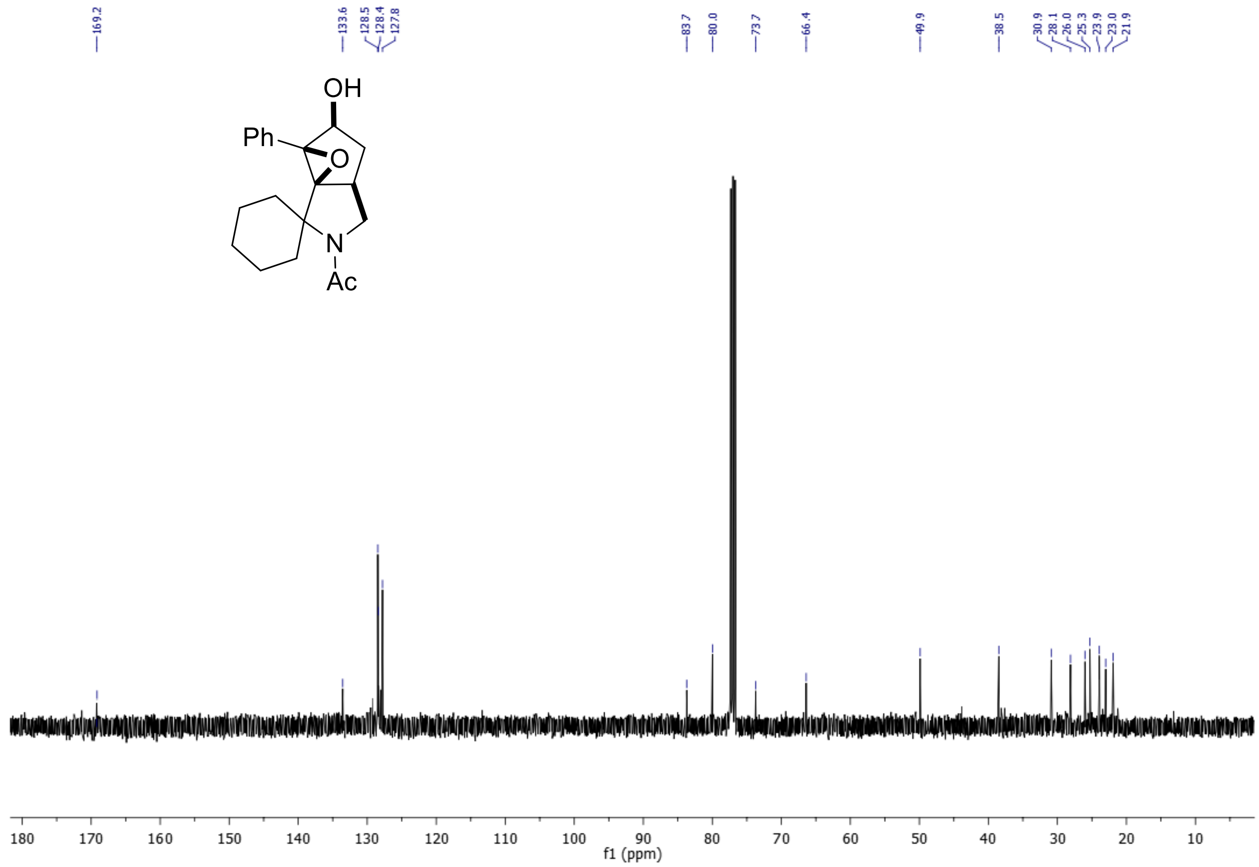

Figure S50. ^{13}C NMR spectrum of compound **37** (100 MHz, $CDCl_3$).

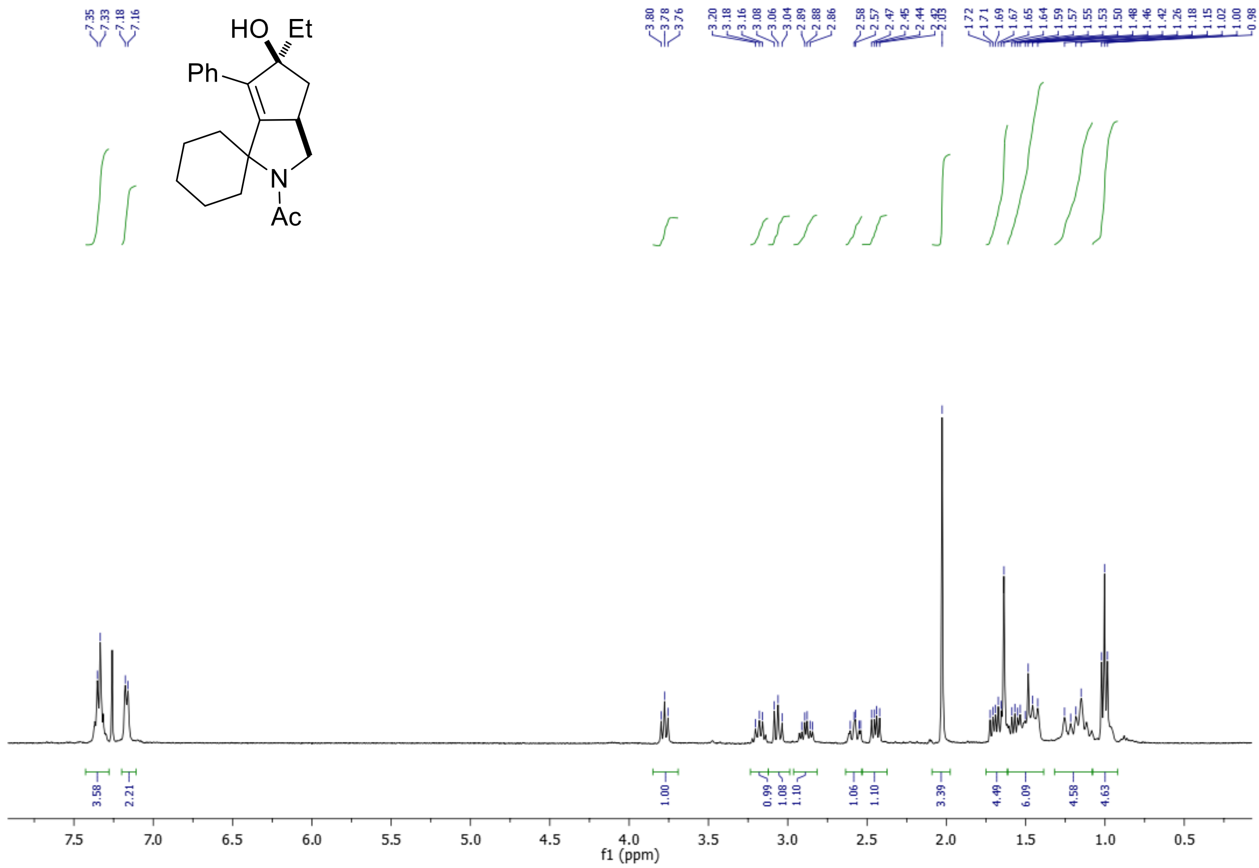

Figure S51. ^{1}H NMR spectrum of compound **38** (400 MHz, $CDCl_3$).

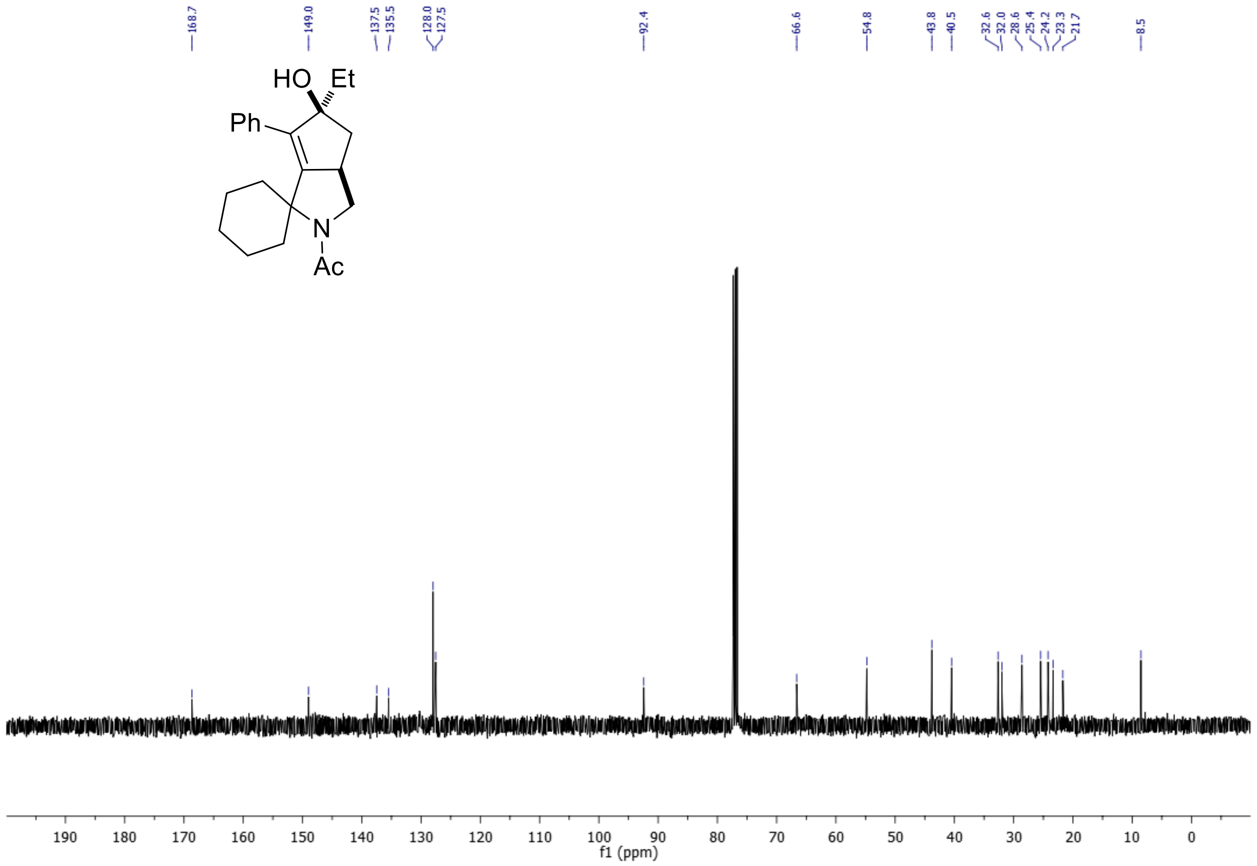

Figure S52. ^{13}C NMR spectrum of compound **38** (100 MHz, $CDCl_3$).

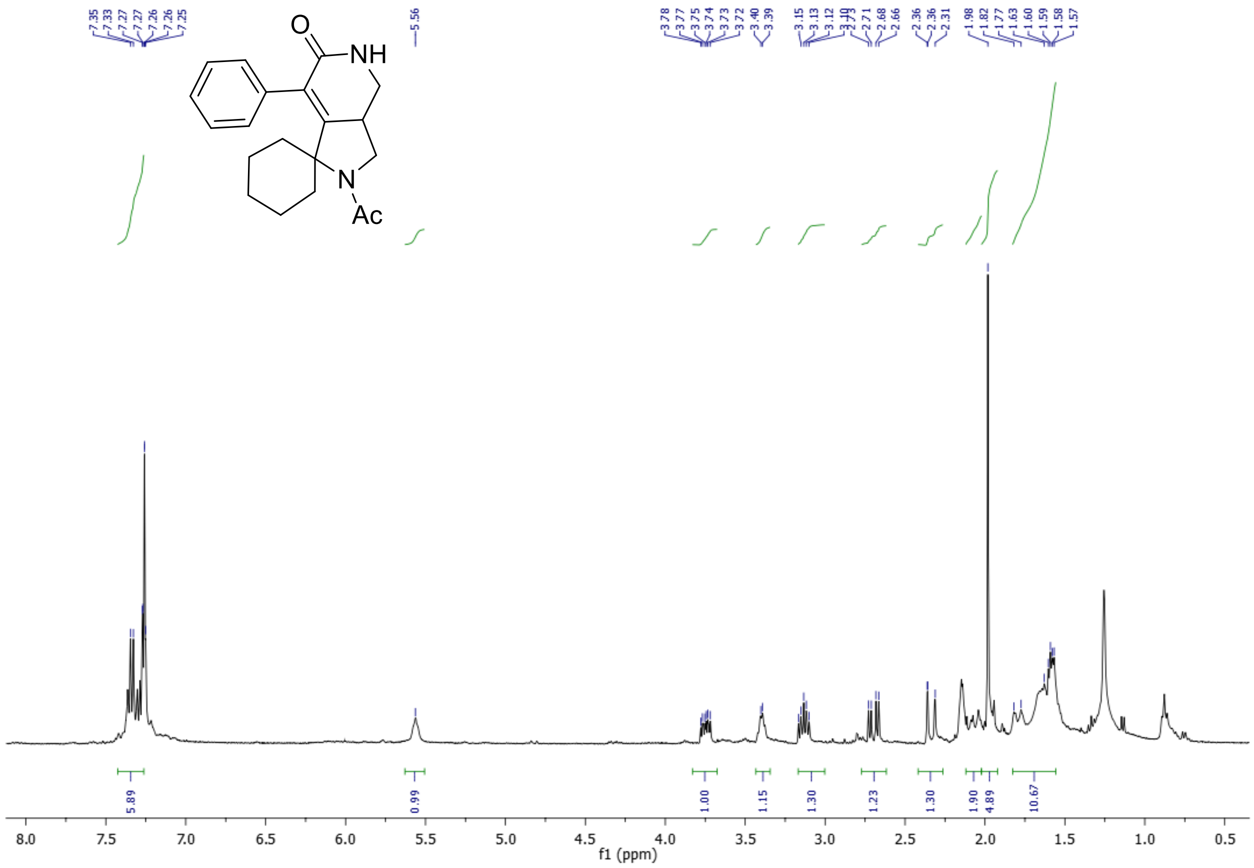

Figure S53. ^{1}H NMR spectrum of compound **39** (400 MHz, $CDCl_3$).

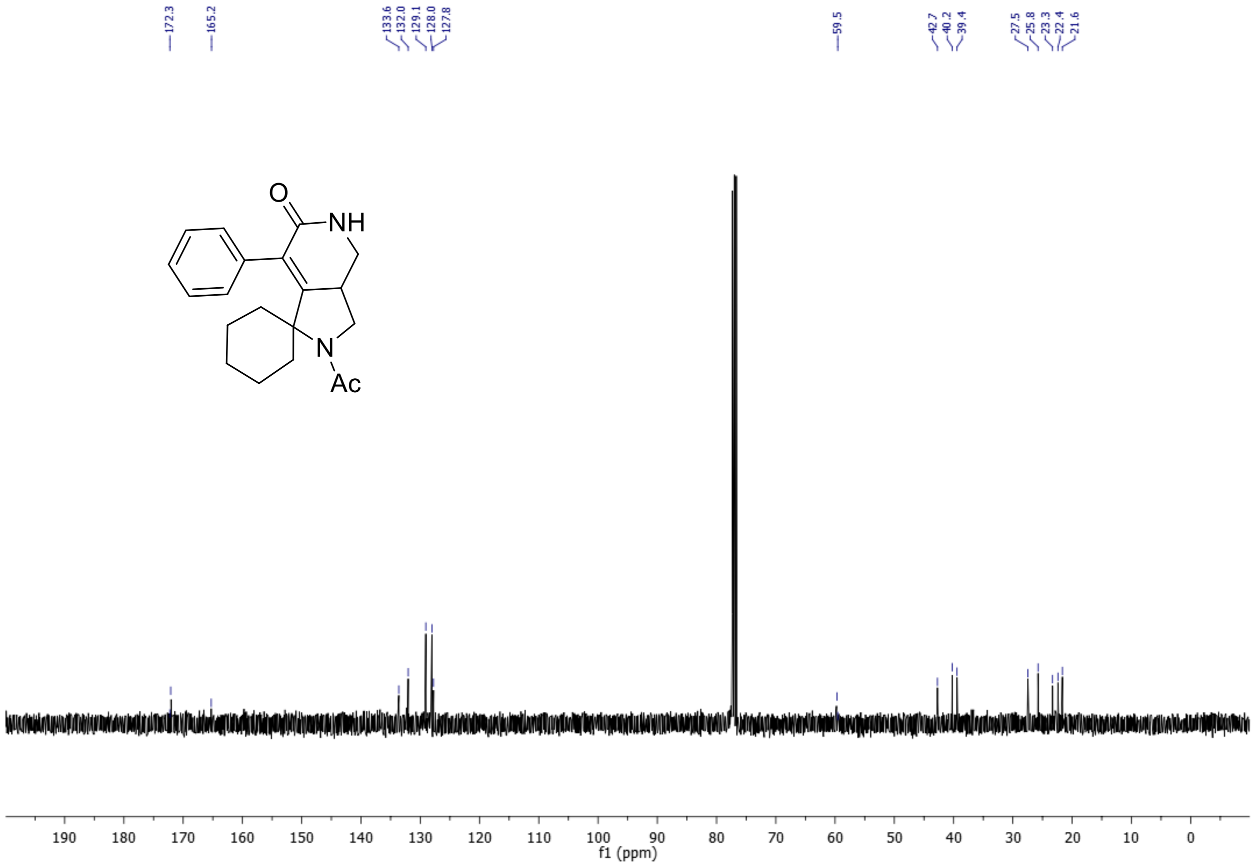

Figure S54. ^{13}C NMR spectrum of compound **39** (100 MHz, $CDCl_3$).

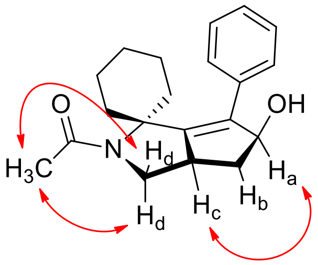

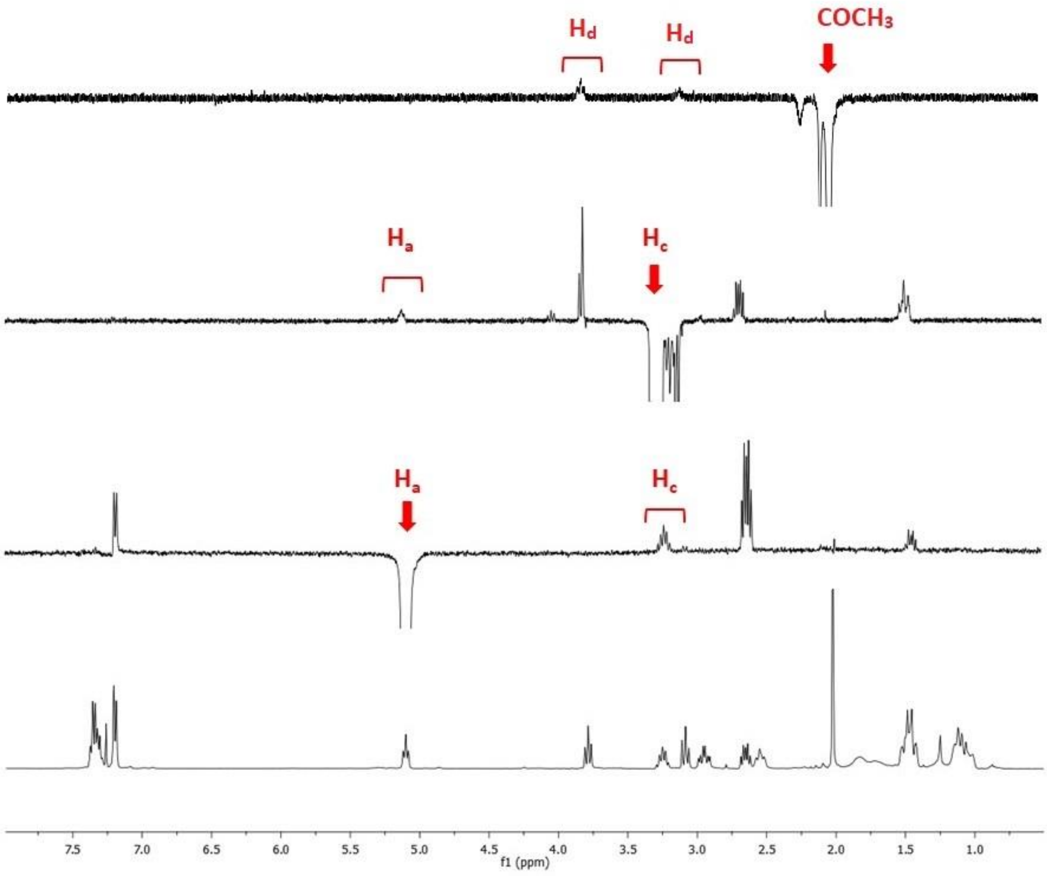

Figure S55. Selected NOE contacts from NOESY 1D spectra of compound **36** (400 MHz, $CDCl_3$).

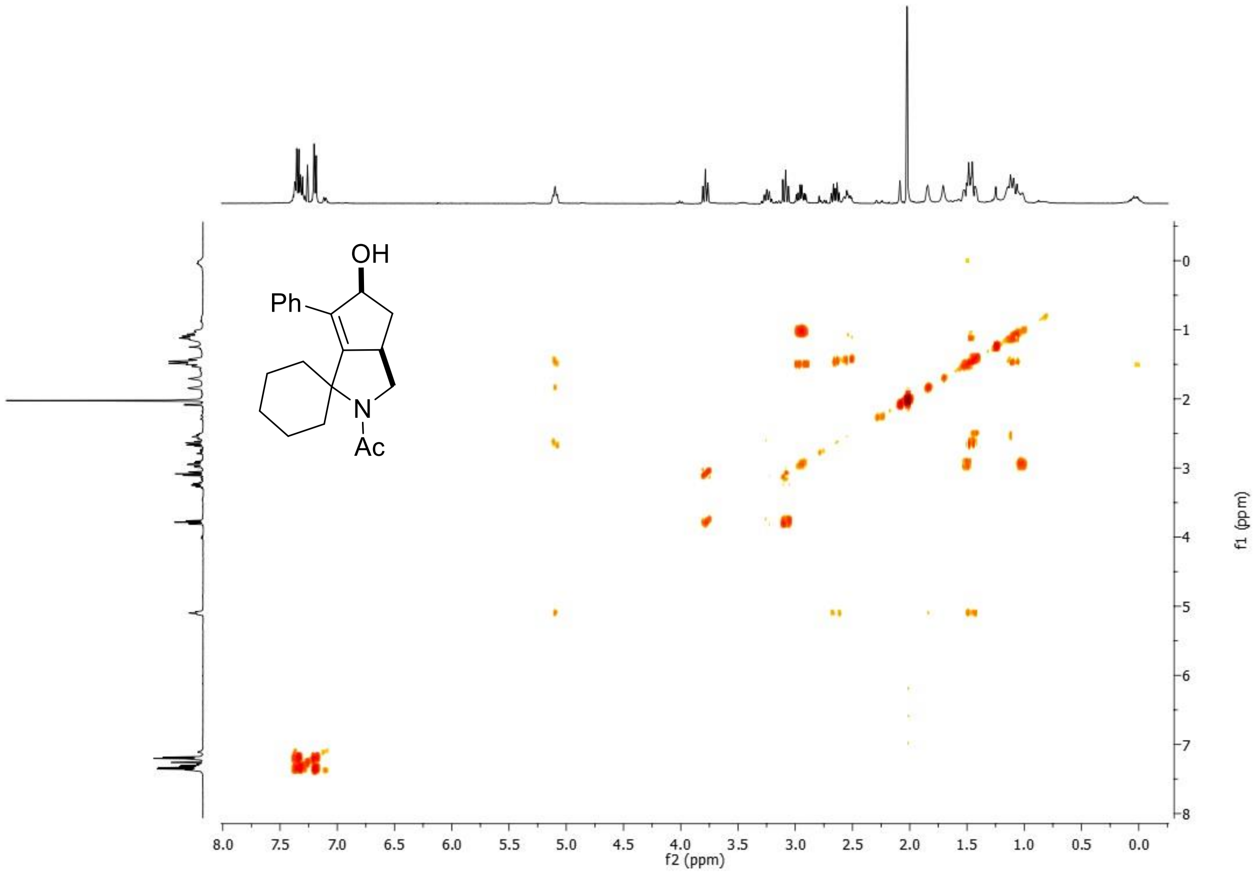

Figure S56. gCOSY spectrum of compound **36** (400 MHz, $CDCl_3$)

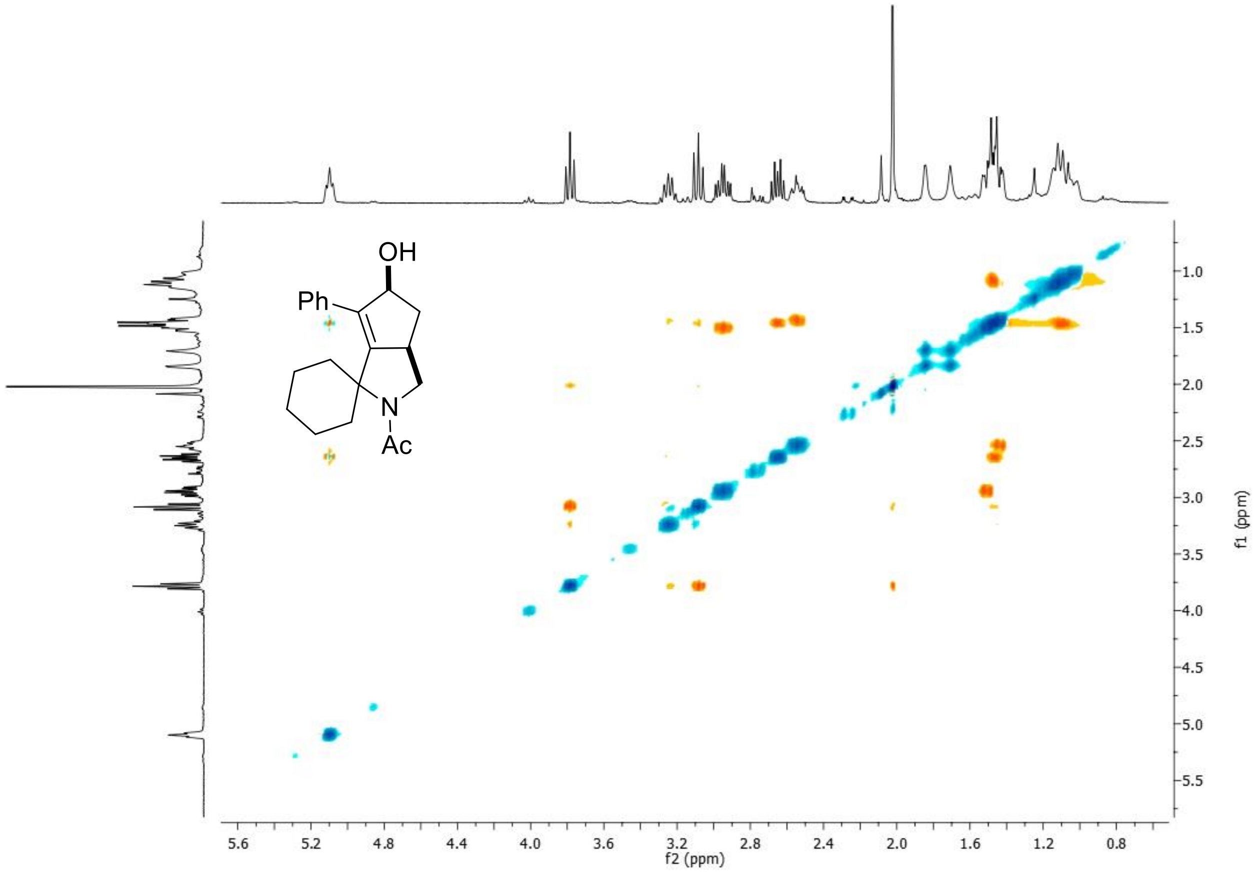

Figure S57. NOESY spectrum of compound **36** (400 MHz, $CDCl_3$).

Table S2. Cartesian coordinates of compound **36** as in the PDB structure:

HETATM	1	N	UNK	0001	-0.843	1.967	1.864	0.00	0.00	N
HETATM	2	C	UNK	0001	1.214	0.970	2.947	0.00	0.00	C
HETATM	3	C	UNK	0001	1.434	2.818	1.136	0.00	0.00	C
HETATM	4	C	UNK	0001	2.832	2.886	3.244	0.00	0.00	C
HETATM	5	C	UNK	0001	2.116	3.700	2.178	0.00	0.00	C
HETATM	6	C	UNK	0001	1.889	1.916	3.937	0.00	0.00	C
HETATM	7	C	UNK	0001	0.611	1.624	1.680	0.00	0.00	C
HETATM	8	C	UNK	0001	-1.717	1.175	0.994	0.00	0.00	C
HETATM	9	C	UNK	0001	0.478	0.611	0.538	0.00	0.00	C
HETATM	10	C	UNK	0001	-0.839	0.765	-0.156	0.00	0.00	C
HETATM	11	C	UNK	0001	1.156	-0.437	0.036	0.00	0.00	C
HETATM	12	C	UNK	0001	-1.065	-0.600	-0.777	0.00	0.00	C
HETATM	13	C	UNK	0001	0.351	-1.124	-1.042	0.00	0.00	C
HETATM	14	C	UNK	0001	2.482	-0.914	0.415	0.00	0.00	C
HETATM	15	C	UNK	0001	5.030	-1.822	1.147	0.00	0.00	C
HETATM	16	C	UNK	0001	2.628	-2.074	1.186	0.00	0.00	C
HETATM	17	C	UNK	0001	3.625	-0.213	0.015	0.00	0.00	C
HETATM	18	C	UNK	0001	4.894	-0.665	0.380	0.00	0.00	C
HETATM	19	C	UNK	0001	3.897	-2.526	1.551	0.00	0.00	C
HETATM	20	C	UNK	0001	-1.335	2.862	2.796	0.00	0.00	C
HETATM	21	C	UNK	0001	-2.838	2.966	2.908	0.00	0.00	C
HETATM	22	O	UNK	0001	0.348	-2.535	-0.931	0.00	0.00	O
HETATM	23	O	UNK	0001	-0.635	3.571	3.512	0.00	0.00	O
HETATM	24	H	UNK	0001	0.452	0.381	3.474	0.00	0.00	H
HETATM	25	H	UNK	0001	2.236	2.438	0.490	0.00	0.00	H
HETATM	26	H	UNK	0001	3.659	2.329	2.786	0.00	0.00	H
HETATM	27	H	UNK	0001	1.394	4.377	2.643	0.00	0.00	H
HETATM	28	H	UNK	0001	1.147	2.462	4.525	0.00	0.00	H
HETATM	29	H	UNK	0001	1.994	0.258	2.668	0.00	0.00	H
HETATM	30	H	UNK	0001	0.811	3.451	0.489	0.00	0.00	H
HETATM	31	H	UNK	0001	3.276	3.561	3.985	0.00	0.00	H
HETATM	32	H	UNK	0001	2.842	4.349	1.673	0.00	0.00	H
HETATM	33	H	UNK	0001	2.457	1.322	4.664	0.00	0.00	H
HETATM	34	H	UNK	0001	-2.579	1.754	0.651	0.00	0.00	H
HETATM	35	H	UNK	0001	-2.080	0.322	1.579	0.00	0.00	H
HETATM	36	H	UNK	0001	-0.767	1.557	-0.912	0.00	0.00	H
HETATM	37	H	UNK	0001	-1.647	-0.535	-1.703	0.00	0.00	H
HETATM	38	H	UNK	0001	-1.618	-1.272	-0.108	0.00	0.00	H
HETATM	39	H	UNK	0001	0.705	-0.836	-2.038	0.00	0.00	H
HETATM	40	H	UNK	0001	1.749	-2.624	1.519	0.00	0.00	H
HETATM	41	H	UNK	0001	3.537	0.689	-0.586	0.00	0.00	H
HETATM	42	H	UNK	0001	5.778	-0.114	0.068	0.00	0.00	H
HETATM	43	H	UNK	0001	4.000	-3.424	2.155	0.00	0.00	H
HETATM	44	H	UNK	0001	6.018	-2.171	1.434	0.00	0.00	H
HETATM	45	H	UNK	0001	1.268	-2.824	-1.063	0.00	0.00	H
HETATM	46	H	UNK	0001	-3.290	1.977	3.024	0.00	0.00	H
HETATM	47	H	UNK	0001	-3.244	3.475	2.030	0.00	0.00	H
HETATM	48	H	UNK	0001	-3.094	3.552	3.797	0.00	0.00	H
CONECT	1	7	8	20						
CONECT	2	6	7	24	29					
CONECT	3	5	7	25	30					
CONECT	4	5	6	26	31					
CONECT	5	3	4	27	32					
CONECT	6	2	4	28	33					
CONECT	7	1	2	3	9					
CONECT	8	1	10	34	35					
CONECT	9	7	10	11						
CONECT	10	8	9	12	36					
CONECT	11	9	13	14						
CONECT	12	10	13	37	38					

```
CONECT   13   11   12   22   39
CONECT   14   11   16   17
CONECT   15   18   19   44
CONECT   16   14   19   40
CONECT   17   14   18   41
CONECT   18   15   17   42
CONECT   19   15   16   43
CONECT   20    1   21   23
CONECT   21   20   46   47   48
CONECT   22   13   45
CONECT   23   20
CONECT   24    2
CONECT   25    3
CONECT   26    4
CONECT   27    5
CONECT   28    6
CONECT   29    2
CONECT   30    3
CONECT   31    4
CONECT   32    5
CONECT   33    6
CONECT   34    8
CONECT   35    8
CONECT   36   10
CONECT   37   12
CONECT   38   12
CONECT   39   13
CONECT   40   16
CONECT   41   17
CONECT   42   18
CONECT   43   19
CONECT   44   15
CONECT   45   22
CONECT   46   21
CONECT   47   21
CONECT   48   21
END
```

Table S3. SMILES codes of newly-synthesized compounds **3**–**39**.

C=CCNC1(C#CC2=CC=CC=C2)CCCCC1 [**3**]
C=CCN(C(C)=O)C1(C#CC2=CC=CC=C2)CCCCC1 [**4**]
O=C1CC(CN2C(C)=O)C(C32CCCCC3)=C1C4=CC=CC=C4 [**5**]
C=CCNC1(C#CC2=CC=CS2)CCCCC1 [**7**]
C=CCN(C(C)=O)C1(C#CC2=CC=CS2)CCCCC1 [**8**]
O=C1CC(CN2C(C)=O)C(C32CCCCC3)=C1C4=CSC=C4 [**9**]
C=CCNC1(CCCC1)C#CC2=CC=CC=C2 [**13**]
C=CCN(C(C)=O)C1(CCCC1)C#CC2=CC=CC=C2 [**14**]
O=C1CC2C(C3(CCCC3)N(C(C)=O)C2)=C1C4=CC=CC=C4 [**15**]
C=CCNC1(C#CC2=CC=CC=C2)CCC(C)CC1 [**17**]
C=CCN(C(C)=O)C1(C#CC2=CC=CC=C2)CCC(C)CC1 [**18**]
O=C1CC(CN2C(C)=O)C(C32CCC(C)CC3)=C1C4=CC=CC=C4 [**19**]
C=CCNC1(C#CC2=CC=CC=C2)CCN(C(OC(C)(C)C)=O)CC1 [**24**]
C=CCN(C(C)=O)C1(C#CC2=CC=CC=C2)CCN(C(OC(C)(C)C)=O)CC1 [**25**]
O=C1CC(CN2C(C)=O)C(C32CCN(C(OC(C)(C)C)=O)CC3)=C1C4=CC=CC=C4 [**26**]
C=CCNC1(C#CC2=CSC=C2)CCN(C(OC(C)(C)C)=O)CC1 [**27**]
C=CCN(C(C)=O)C1(C#CC2=CSC=C2)CCN(C(OC(C)(C)C)=O)CC1 [**28**]
O=C1CC(CN2C(C)=O)C(C32CCN(C(OC(C)(C)C)=O)CC3)=C1C4=CC=CS4 [**29**]
C=CCN(C(C1=CC=CC=C1)=O)C2(C#CC3=CC=CC=C3)CCN(C(OC(C)(C)C)=O)CC2 [**30**]
O=C1CC(C2=C1C3=CC=CC=C3)CN(C(C4=CC=CC=C4)=O)C52CCN(C(OC(C)(C)C)=O)CC5 [**31**]
C=CCN(S(C1=CC=C(C)C=C1)(=O)=O)C2(C#CC3=CC=CC=C3)CCN(C(OC(C)(C)C)=O)CC2 [**32**]
C=CCNC1(C#CC2=CC=CC=C2)CCN(S(C3=CC=C(C)C=C3)(=O)=O)CC1 [**34**]
C=CCN(C(C)=O)C1(C#CC2=CC=CC=C2)CCN(S(C3=CC=C(C)C=C3)(=O)=O)CC1 [**35**]
O[C@H]1C[C@@H]2C(C3(CCCCC3)N(C(C)=O)C2)=C1C4=CC=CC=C4 [**36**]
O[C@H]([C@@]1(O2)C3=CC=CC=C3)C[C@@H]4[C@]12C5(CCCCC5)N(C(C)=O)C4 [**37**]
O[C@@]1(CC)C[C@@H]2C(C3(CCCCC3)N(C(C)=O)C2)=C1C4=CC=CC=C4 [**38**]
O=C1NCC2C(C3(CCCCC3)N(C(C)=O)C2)=C1C4=CC=CC=C4 [**39**]

Table S4. PCA results table for the first four dimensions of compounds **3-39** (77% of data variance, as reported[1]; data for BB drugs are reported[2]).

MOLID	PS1	PS2	PS3	PS4
[**3**]	-2,7247880	0,7987790	1,0410250	-0,397687
[**4**]	-2,1512250	0,6595260	1,1669390	-0,147932
[**5**]	-1,6808870	0,6332040	0,8340450	-0,676776
[**7**]	-2,9709880	0,3519120	0,7944920	-0,236589
[**8**]	-2,3975970	0,2173330	0,9222180	-0,000860
[**9**]	-1,9306430	0,1893000	0,5383170	-0,550722
[**17**]	-2,5479180	0,7064170	1,1926450	-0,460735
[**18]**	-1,9741770	0,5708770	1,3239130	-0,220148
[**19**]	-1,5020360	0,5444760	0,9940120	-0,727077
[**25**]	-0,7124530	0,1906550	1,2067520	0,217829
[**26**]	-0,2363510	0,1736470	0,8973640	-0,277458
[**27**]	-1,5311490	-0,1346300	0,7684920	0,143850
[**28**]	-0,9585870	-0,2573510	0,9162160	0,347550
[**29**]	-0,4835390	-0,2606540	0,6533650	-0,146434
[**31**]	0,9470730	1,4749850	1,7179750	-0,316100
[**32**]	0,9486110	1,4695560	2,2017410	0,257289
[**34**]	-0,3362750	1,9151320	1,4244710	0,035965
[**35**]	0,2329640	1,7459930	1,5635560	0,251427
[**36**]	-1,5568340	0,3941490	0,5897720	-0,930972
[**37**]	-1,3610130	0,2987730	0,3101600	-1,110.214
[**38**]	-1,1675350	0,1929900	0,9774660	-0,869766
[**39**]	-1,2681120	0,4766400	0,4326810	-0,445033

[1] (a) Larsson, J.; Gottfries, J.; Muresan, S.; Backlund, A. *J. Nat. Prod.* **2007**, *70*, 789-794; Rosén, J.; Lövgren, A.; Kogej, T.; Muresan, S.; Gottfries, J.; Backlund, A. *J. Comput. Aided Mol. Des.* **2009**, *23*, 253-259.
[2] Lenci, E.; Menchi, G.; Guarna, A.; Trabocchi, A. *J. Org. Chem.* **2015**, *80*, 2182-2191

Table S5. PMI ratio calculated for compounds **3**–**39** (data for BB drugs are reported[2]).

**MOLID**	**I_1**	**I_2**	**I_3**	**I_3/I_1**	**I_2/I_1**
[**3**]	3111,9850	2645,5832	739,8855	0,2378	0,8501
[**4**]	3369,4838	2854,3947	903,6792	0,2682	0,8471
[**5**]	2712,0175	2296,7629	1289,0403	0,4753	0,8469
[**7**]	2785,7151	2352,4610	806,7296	0,2896	0,8445
[**8**]	3192,7712	2789,2532	893,6944	0,2799	0,8736
[**9**]	3195,0185	2333,8091	1174,8782	0,3677	0,7305
[**13**]	2474,8288	1758,8212	879,2849	0,3553	0,7107
[**14**]	2963,5174	2452,9160	818,0395	0,2760	0,8277
[**15**]	2680,3460	2191,9054	1150,3171	0,4292	0,8178
[**17**]	3193,2808	2438,0791	1112,6207	0,3484	0,7635
[**18**]	3577,7764	2787,9853	1297,0913	0,3625	0,7793
[**19**]	2897,6303	2400,5773	1563,5148	0,5396	0,8285
[**24**]	6012,3273	4230,0304	2286,6966	0,3803	0,7036
[**25**]	4924,1262	3965,6326	2023,6716	0,4110	0,8053
[**26**]	5719,7941	4443,4407	2561,8897	0,4479	0,7769
[**27**]	5893,7715	4112,2356	2325,6853	0,3946	0,6977
[**28**]	5022,0553	3969,5732	2070,2931	0,4122	0,7904
[**29**]	6434,8851	5139,6846	1838,7006	0,2857	0,7987
[**30**]	8276,6087	5162,8274	4212,7867	0,5090	0,6238
[**31**]	8820,6609	5567,5884	4355,1182	0,4937	0,6312
[**32**]	8628,6593	7451,6571	2774,6882	0,3216	0,8636
[**34**]	7974,4679	6117,3092	2322,1633	0,2912	0,7671
[**35**]	8420,6862	6410,3277	2732,9802	0,3246	0,7613
[**36**]	3227,5566	2313,0151	1189,6349	0,3686	0,7166
[**37**]	3129,0426	2244,2223	1381,8542	0,4416	0,7172
[**38**]	3526,9533	2551,4946	1525,1176	0,4324	0,7234
[**39**]	3305,9890	2255,9258	1382,8892	0,4183	0,6824
